# Identification of a prognostic chemoresistance-related gene signature associated with immune microenvironment in breast cancer

**DOI:** 10.1080/21655979.2021.1977768

**Published:** 2021-10-18

**Authors:** Mingzhou Liu, Qiaoyan Li, Ningmin Zhao

**Affiliations:** a Department of Pharmacy, Henan Provincial People’s Hospital, Zhengzhou University People’s Hospital, Zhengzhou, Henan, China; b Tissue Engineering Laboratory, Henan Eye Institute, Henan Eye Hospital, Henan Provincial People’s Hospital, Zhengzhou University People’s Hospital, Zhengzhou, China

**Keywords:** Chemoresistant breast cancer, prognostic gene signature, tumor microenvironment, weighted co-expression network analysis, chemoresistance-related genes

## Abstract

Breast cancer is the most common form of cancer among women globally, and chemoresistance is a major challenge to disease treatment that is associated with a poor prognosis. This study was formulated to identify a reliable prognostic biosignature capable of predicting the survival of patients with chemoresistant breast cancer (CRBC) and evaluating the associated tumor immune microenvironment. Through a series of protein-protein interaction and weighted correlation network analyses, genes that were significantly associated with breast cancer chemoresistance were identified. Moreover, univariate Cox regression and lasso-penalized Cox regression analyses were employed to generate a prognostic model, and the prognostic utility of this model was then assessed using time-dependent receiver operating characteristic (ROC) and Kaplan-Meier survival curves. Finally, The CIBERSORT and ESTIMATE algorithms were additionally leveraged to assess relationships between the tumor immune microenvironment and patient prognostic signatures. Overall, a multigenic prognostic biosignature capable of predicting CRBC patient risk was successfully developed based on bioinformatics analysis and in vitro experiments. This biosignature was able to stratify CRBC patients into high- and low-risk subgroups. ROC curves also revealed that this biosignature achieved high diagnostic efficiency, and multivariate regression analyses indicated that this risk signature was an independent risk factor linked to CRBC patient outcomes. In addition, this signature was associated with the infiltration of the tumor microenvironment by multiple immune cell types. In conclusion, the chemoresistance-associated prognostic gene signature developed herein was able to effectively evaluate the prognosis of CRBC patients and to reflect the overall composition of the tumor immune microenvironment.

## Introduction

Breast cancer is the most prevalent form of malignancy among women and the leading cause of female cancer-related mortality in the world [1]. Adjuvant treatments for breast cancer primarily consist of chemotherapy regimens composed of taxanes and anthracyclines [2], but the acquisition of chemoresistance and eventual disease recurrence or relapse are common, resulting in a poor patient prognosis [2–4]. Treatment options are currently unable to overcome such chemoresistance in most cases, and differentiating between low- and high-risk patients based upon traditional clinicopathological risk factors is often ineffective as a means of prognostic evaluation [5,6]. Novel approaches to stratifying chemoresistant breast cancer (CRBC) patients according to their risk levels are thus essential. The tumor microenvironment plays an integral role in shaping the onset and progression of breast cancer, and the presence of different immune cells within this tumor niche can influence the development ofchemoresistance [7,8]. As high-throughput sequencing and bioinformatics technologies have continued to advance, they have aided in the development of more reliable diagnostic and prognostic signatures capable of accurately evaluating specific patient populations [9,10]. While an individual gene may be of limited utility as a predictive biomarker, multi-gene signatures offer significant advantages in this context [11]. To date, however, there have been few studies evaluating multigenic prognostic biosignatures in CRBC patient populations. We thus hypothesized that chemoresistance-related genes can serve as a valuable prognostic biosignature for CRBC patient evaluation, in addition to serving as viable therapeutic targets. As such, we herein sought to design and validate a novel signature composed of chemoresistance-related genes capable of stratifying CRBC patients based upon their relative risk. We first identified and validated differentially expressed chemoresistance-related genes so as to develop a risk signature related to CRBC patient survival. Protein-protein interaction networks (PPINs) were further established, relevant biological functions and pathways were identified, and relevant candidate drugs were predicted based on this prognostic signature. Finally, The correlations between risk score values and immune cell infiltration were analyzed using the CIBERSORT and ESTIMATE algorithms. Overall, the multi-gene signature established herein exhibits substantial clinical prognostic utilityand can effectively characterize the tumor immune microenvironment in CRBC patients. Therefore, this signature may aid in the identification of those patients likely to benefit from immunotherapy and it thus has the potential to aid clinicians in the development of individualized treatment strategies for CRBC patients.

## Results

A growing body of evidence has shown that chemoresistance plays an important role in breast cancer development and progression, in addition to shaping patient treatment responses and overall prognosis. Therefore, we hypothesized that the bioinformatics-based identification of a chemoresistance-related prognostic gene signature may highlight new avenues toward understanding breast cancer chemoresistance and predicting the survival of patients undergoing taxane and anthracycline-based neoadjuvant chemotherapy. In the present study, we developed and validated a chemoresistance-related biosignature as a means of predicting CRBC patient prognosisand thereby aiding in the design of personalized treatments.

### Differentially expressed gene screening

**Figure 1.**
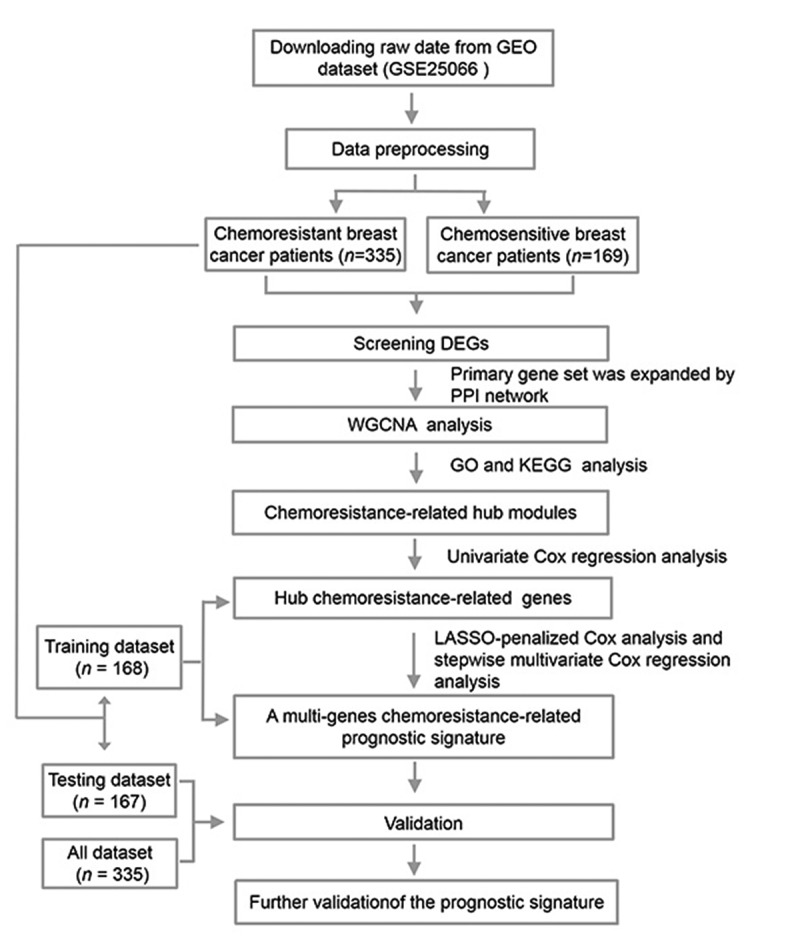
A flow chart outlining the development of multi-gene prognostic signatures pertaining to chemoresistant breast cancerpatient prognosis, including data collection, preprocessing, analysis, and validation

**Figure 2.**
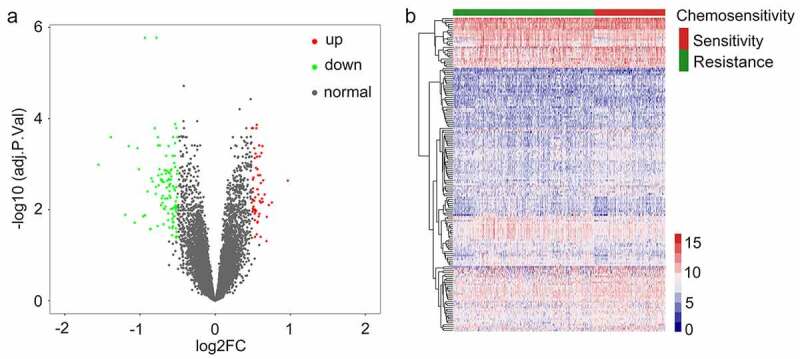
Identifying genes that exhibited differential expression. (a) A volcano plot showing the expression difference between chemosensitive and chemoresistant breast cancer patients. Red color is representative of expression that was up-regulated, and green color represents expression that was down-regulated. (b) The heatmap of the patients with chemosensitivity of breast cancer as compared to samples that were normal. A cutoff with absolute value of log2FC > 1 as well as an FDR < 0.05 was utilized for defining genes that exhibited differential expression

An overview of our study approach is provided in Figure 1. Briefly, a publically available dataset (GSE25066) was downloaded from the GEO database (https://www.ncbi.nlm.nih.gov/). This dataset was composed of transcriptomic data pertaining to 508 patients with breast cancer that were undergoing neoadjuvant taxane-anthracycline-based chemotherapy, of whom 169 were chemosensitive and achieved a complete response to treatment, whereas 339 were found to have CRBC and to have experienced stable or progressive disease during treatment. Patient clinical characteristics are detailed in Supplementary Table 1. The ‘limma’ R package was used to identified chemoresistance-related genes (CRGs) that were differentially expressed between these two groups of patients. In total, 163 CRGs were identified (110 upregulated and 53 downregulated) with a false discovery rate (FDR) threshold of < 0.05 and an absolute log2 fold change (FC) > 1. These identified CRGs are were arranged in volcano plots and heatmaps (Figure 2(a,b)), and are listed in Supplementary Table 2.

### Functional enrichment analyses of identified CRGs

**Figure 3.**
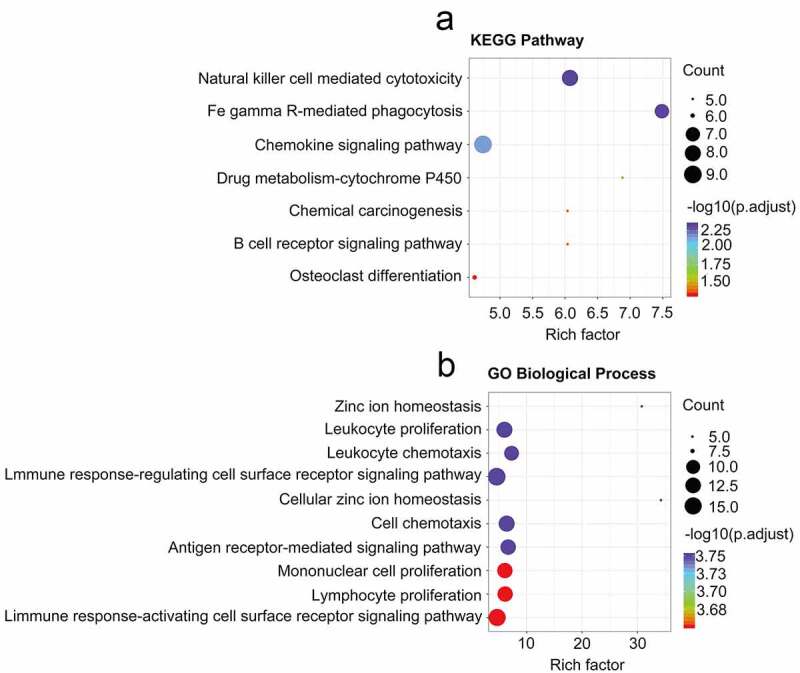
Analysis related to functional enrichment in the context of GO biologic processes as well as KEGG pathway enrichment of differentially expressed genes (DEGs). (a) DEG analyses via KEGG enrichment (every KEGG pathway that was significant). (b) DEG analyses based on GO terms enrichment (the top ten terms are described in every GO category). KEGG as well as GO analysis was conducted with the use of the online instrument known as DAVID, and the cutoff criteria with regards to FDR was less than 0.05. The color that is associated with each of the bubbles is representative of the FDR in relation to that specific term, with the color red being representative of significance that was greater. The proportion of genes that are enriched for every term is referred to via the rich factor

The DAVID tool was next used to conduct functional enrichment analyses of identified CRGs. The primary KEGG pathways in which these genes were enriched were identified (Figure 3(a)), and included the natural killer cell-mediated cytotoxicity, FcγR-mediated phagocytosis, chemokine, drug metabolism-cytochrome P450, Chemical carcinogenesis, B cell receptor, and osteoclast differentiation signaling pathways (Supplementary Table 3). GO terms in which these CRGs were enriched were also analyzed (Supplementary Table 4), and included the zinc ion homeostasis signaling pathway, the leukocyte proliferation signaling pathway, leukocyte chemotaxis, the immune response-regulating cell surface receptor signaling pathway, cellular zinc ion homeostasis, cell chemotaxis, antigen receptor-mediated signaling pathways, mononuclear cell proliferation, lymphocyte proliferation, and the immune response-activating cell surface receptor signaling pathways (Figure 3(b)).

### Protein-protein interaction-mediated establishment of a core gene co-expression network associated with CRBC

**Figure 4.**
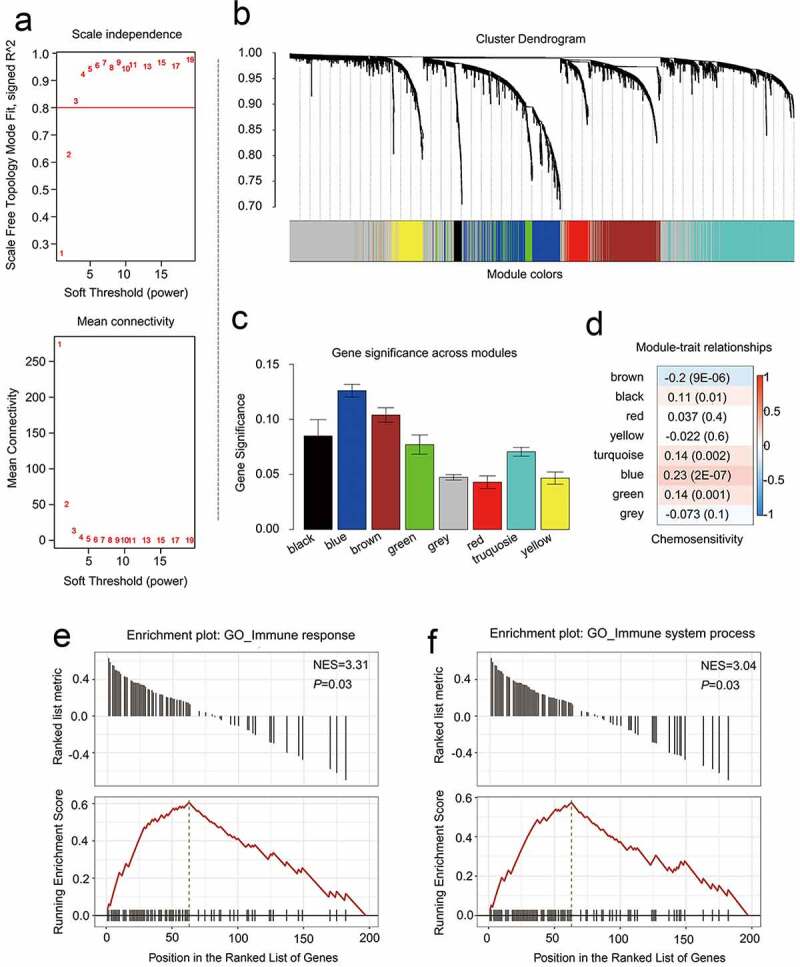
Identifying modules that are linked to the chemosensitivity status of cancer of the breast within the WGCNA. (a) The fit index that was scale-free for powers that involve soft-thresholding. Determination of the power involving soft-thresholding within the WGCNA was accomplished on the basis of a R^2^ that was scale-free (specifically R^2^ = 0.95). The panel at the top illustrates the association that exists between soft-threshold as well as scale-free R^2^. The panel at the bottom illustrates the association between mean connectivity as well as the soft-threshold. (b) A dendrogram depicting the genes that exhibited differential expression clustered on the basis of metrics that are different. Every branch within the figure is representative of one gene, and each color underneath is representative of one module of co-expression. (c) A heatmap that depicts the correlation that exists with regards to the gene module as well as clinical attributes. The brown module was composed of 338 DEGs while the blue module was composed of 360 DEGs. The coefficient of correlation within each cell is representative of the correlation that exists between the chemosensitive phenotype and the gene module, increasing in size from blue to red. The blue module demonstrated a positive correlation that was highest with regards to survival whereas the red module demonstrated the negative correlation that was the highest with regards to survival. (d) Distributive pattern of average gene-based significance as well as errors within the modules related to DFS in CRBC. On the basis of the hierarchical clustering with average linkage as well as the power involving soft-thresholding, the identification of eight modules was subsequently accomplished. To facilitate the determination of the significance associated with every module, gene significance (or GS) was ascertained for measuring the correlation that exists between chemosensitive phenotype and genes. GS was given the definition of a log^10^ conversion directed at the *p*-value in the context of the linear regression between clinical data and gene expression (i.e. GS = lgP). The module that is brown and blue exhibited a high degree of correlation with survival in patients who had CRBC. GSEA results showed that there was a positive enrichment of two immune related terms in the 201 genes associated with patient disease-free survival (e and f)

PPINs analyses have led researchers to conclude that the closely associated genes in these networks may represent shared predictive markers of chemosensitivity [12,13]. To apply such findings to the present study, the CRGs identified above were treated as a primary gene set, and neighboring proteins predicted to directly interact with them were then incorporated into an expanded interaction network, yielding a greatly expanded gene set containing 3075 genes. Next, 2605 candidate genes within this gene set were screened in the GSE25066 dataset using the WGCNA R package to construct a weighted co-expression network [14]. A cluster analysis approach was used to group 503 patients with similar gene expression patterns (other than 5 outliers) into modules, with a *β *= 3 power (scale-free *R*^2^ = 0.95) being selected for soft-thresholding to ensure a scale-free network (Figure 4(a)), In total, this led to the identification of 8 modules, of which two containing CRGs were retained for further analyses (Figure 4(b), C; Supplementary Table 5). Breast cancer chemoresistance-related genes were then further analyzed by assessing associations between particular modules and chemoresistance in breast cancer patients, with the modules that were most significantly associated with CRBC being of the greatest value for the prediction of patient prognosis and therapeutic responsiveness. We found that in these breast cancer patient cohorts, chemoresistance was significantly related to the brown (R = −0.2, *p* = 9E-06) and blue (R = 0.23, *p* = 2E-07) modules when conducting a module-feature relationship analysis (Figure 4(c)). These two modules also exhibited the greatest degree of gene significance as pertains to breast cancer chemoresistance (Figure 4(d)). As such, we selected these blue and brown modules for further analysis.

### Evaluation of the prognostic significance of individual genes within the blue and brown modules

To examine the functional roles of the blue and brown modules, 339 CRBC patients from this dataset (excluding four outlier patients) were randomized to yield a training cohort (*n* = 168) and a testing cohort (*n* = 167) according to a computer-generated random allocation sequence. CRBC-related genes were then identified by uploading a list of the 698 genes within the blue and brown modules and performing univariate Cox regression analyses examining the relationship between each of these genes and patient survival. This strategy led to the identification of 201 genes associated with patient disease-free survival (DFS), with these being considered to represent prognostic genes (*p* < 0.05) (Supplementary Table 6). Gene set enrichment analyses (GSEA) additionally examined the enrichment of these 201 genes, and revealed the upregulated genes to be primarily enriched in pathways including the ‘immune response’ and ‘immune system process’ pathways according to the normalized enrichment scores (NES) (Figure 4(e,f); Supplementary Table 7). These 201 genes were also more generally enriched in other processes associated with the immune system such as the chemotaxis of multiple different immune cell types and the regulation of associated responses (Supplementary Table 7). Together, these results suggested that the genes in these two significant chemoresistance-related modules were thus closely linked to enhanced immune functionality.

### Construction and validation of a multigenic biosignature

**Figure 5.**
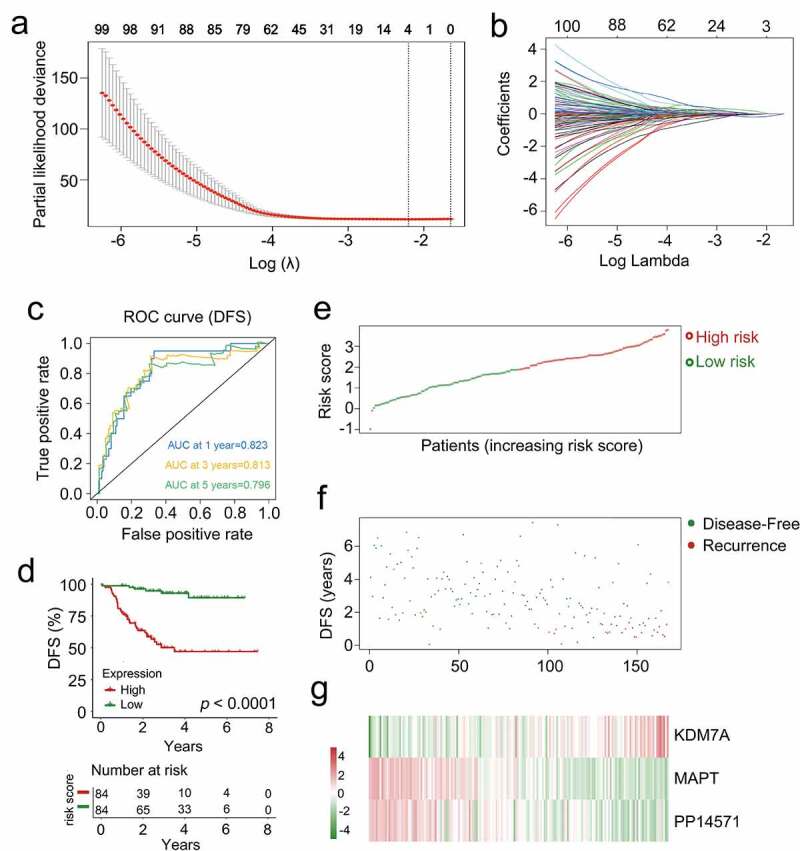
The multi-genes prognostic signature in relation to predicting survival of CRCB patients in the dataset for training. (a) Partial likelihood deviance versus log (λ) was drawn using LASSO-Cox regression model. (b) Coefficients of selected features are shown by lambda parameter. (c) Kaplan-Meier curves related to survival overall between high-risk patients and low-risk patients in the dataset for training. (d) ROC curve associated with the prediction of survival involving multi-genes prognostic signature within a period of five years considered as the training dataset defining point. (E, F, G) The distributive pattern of the three-gene risk scores, overall survival in patients as well as heatmap associated with the three-gene profiles of expression within the dataset for training

In order to identify a list of genes associated with CRBC patient prognosis, the 201 genes identified in univariate Cox regression analyses were subjected to a lasso-penalized Cox analysis to select appropriate parameters for constructing a risk signature (Figure 5(a,b)). Four of the 201 candidate genes (C4A, *KDM7A, MAPT*, and *PP14571*) retained their prognostic significance and may thus impact the prognosis of CRBC (Supplementary Table 8). A subsequent stepwise multivariate Cox regression analysis revealed that three of these genes were truly prognostic (*KDM7A, MAPT, PP14571*) and they were then utilized to guide prognostic model establishment. The resultant model was generated by summing together the products of expression levels for each of these genes multiplied by their relative weighting coefficients derived from the above multivariate Cox regression model as follows: risk score = 0.66179 × (expression level of *KDM7A*) + (−0.38035) × (expression level of *MAPT*) + (−0.16249) × (expression level of *PP14571*). All three of the genes composing this risk scoring model were thus predictors of CRBC patient DFS, with their weighting coefficients corresponding to their overall impact on DFS predictions such that higher levels of *KDM7A* expression and lower levels of *PP14571* and *MAPT* expression were associated with increased chemoresistance. The median risk score value (1.8840) was then used to separate patients into a low-risk and a high-risk cohort, after which the survival time, status, and gene expression levels of patients in these two groups in the training cohort were assessed (Figure 5). As expected, patients in the low-risk cohort exhibited better DFS values relative to patients in the high-risk cohort (*p* < 0.0001, Figure 5(c)). The AUC values for 1-, 3-, and 5-year DFS for these patients were 0.823, 0.813, and 0.796, respectively (Figure 5(d)). These data thus suggested that this signature was a highly effective tool for the prediction of patient DFS, emphasizing the value of multi-genic signatures when predicting the survival of individuals in this training cohort. Risk score distributions, overall survival outcome data, and prognostic gene expression profiles for the training cohort patients are shown in Figure 5(e,f,g) with patients being ranked based on risk score values. Patients with high risk scores exhibited increased mortality, higher levels of *KDM7A* expression, and lower levels of *PP14571* and *MAPT* expression relative to low-risk patients.

### Validation of the prognostic relevance of the multigenic CRBC biosignature

**Figure 6.**
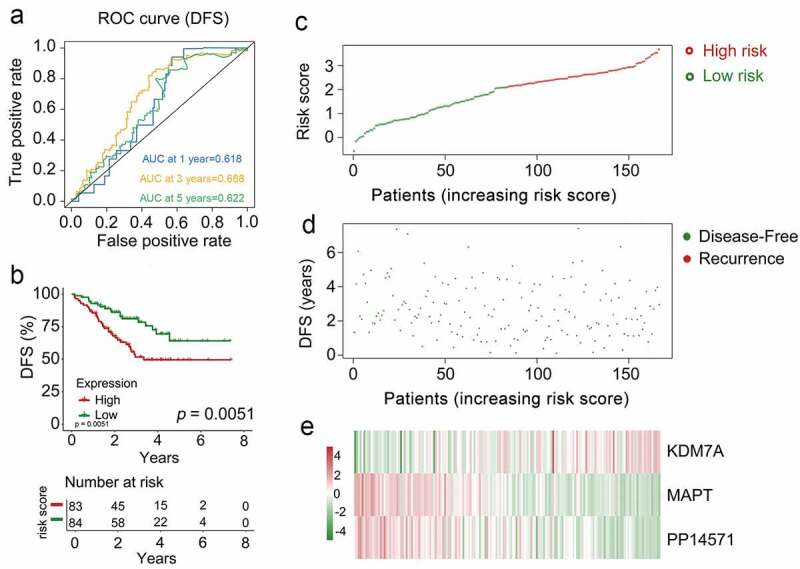
The multi-genes prognostic signature in the prediction of survival in patients who had CRCB within the training dataset. (a) Kaplan-Meier curves related to overall survival between high-risk patients and low-risk patients within the dataset for training. (b) ROC curve in relation to predicting survival via the multi-genes prognostic signature within a period of five years considered as the training dataset defining point. (C, D, E)The distributive pattern of the three-gene risk scores, overall survival in patients as well as heatmap of four-gene profiles of expression within the dataset for training

To confirm the prognostic relevance of our multi-gene biosignature in CRBC patients, the 167 patients in the validation cohort were stratified into high-risk (*n* = 62) and low-risk (*n* = 55) groups based upon the same cutoff used in the training dataset. A significant difference in survival outcomes was observed when comparing these high- and low-risk patients (*p* = 0.0051, Figure 6(a)) with AUC values of 0.618, 0.688, and 0.622 for 1-, 3-, and 5-year DFS, respectively (Figure 6(b)). Risk score, overall survival, and prognostic gene expression profiles for the patients in the validation dataset are shown in Figure 6(c,d,e) with patients being ranked based upon risk scores. As above, patients with high risk scores exhibited increased mortality, higher levels of *KDM7A* expression, and lower levels of *PP14571* and *MAPT* expression relative to low-risk patients.

### Evaluation of the independent prognostic value of the multigenic biosignature as a predictor of patient survival outcomes

**Figure 7.**
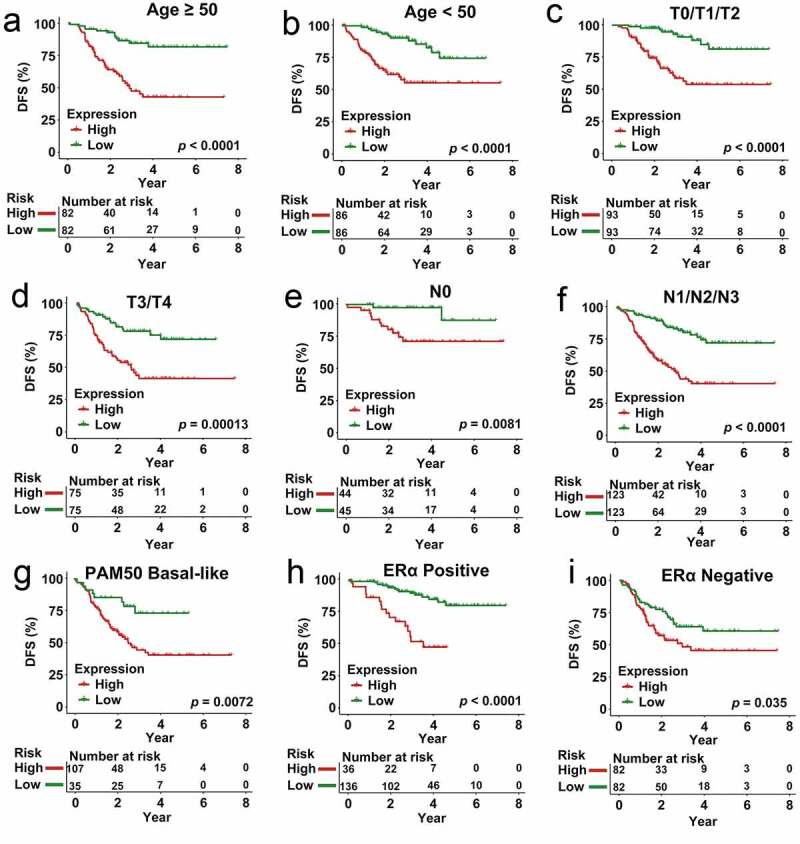
Analysis of survival in all patients who had CRCB stratified on the basis of patient age, staging, subtype of tumor along with the multi-genes prognostic signature. (a) The Kaplan-Meier curves with regards to the dataset that was younger (specifically, age < 50, where *n* = 86). (b) Kaplan-Meier curves in relation to the dataset that was older (specifically, age ≥ 50, where *n* = 148). (c) Kaplan-Meier curves with regards to the dataset related to early stage (specifically stage II/III, where *n* = 156). (d) Kaplan-Meier curves in relation to the dataset with regards to late stage (specifically, stage IV, where *n* = 78). (e) Kaplan-Meier curves in relation to the non-papillary dataset (that is, subtype of non-papillary, where *n* = 165). (f) Kaplan-Meier curves in relation to the papillary dataset (that is, subtype of papillary, where *n* = 69)

To establish whether our multigenic biosignature was an independent predictor of CRBC patient survival outcomes, we next stratified patients in the overall dataset according to their age, T stage, N stage, PAM50 subtype, and ERα subtype, after which Kaplan-Meier analyses were used to compare survival outcomes in each subgroup. In these analyses, individuals in the low-risk group exhibited better DFS relative to patients in the high-risk group in subgroups of patients < 50 years old (*p* < 0.0001), ≥ 50 years old (*p* < 0.0001), and patients with T0/T1/T2 (*p* < 0.0001), T3/T4 (*p* = 0.00013), N0 (*p* = 0.0081), N1/N2/N3 (*p* < 0.0001), ERα Negative (*p* < 0.0001), ERα Positive (*p* = 0.035), and PAM50 Basal-like (*p* = 0.0072) disease (Figure 7). These risk stratification analyses indicated that our prognostic multi-gene biosignature was able to predict CRBC patient outcomes in a manner independent of other patient clinicopathological variables.

### Analyses of the degree of immune cell infiltration in high- and low-risk CRBC patients

**Figure 8.**
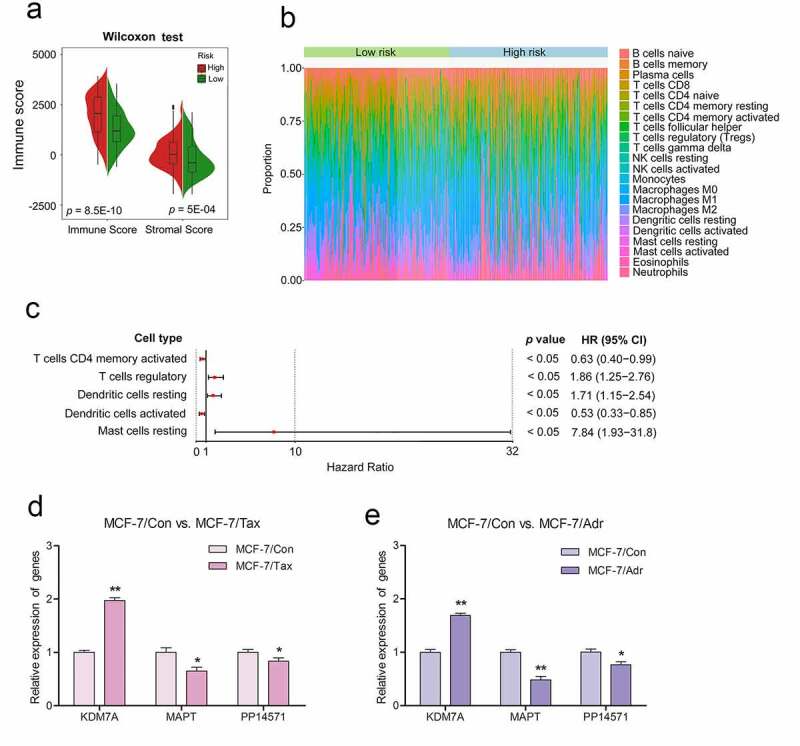
Differences observed when comparing infiltration by immune cells and 22 immune cell subtypes between high-risk CRBC patients and those of a low risk

The tumor microenvironment is a key determinant of breast cancer development, progression, and chemoresistance [15]. The ESTIMATE algorithm was next used to calculate non-tumor cell infiltration in the tumor microenvironment [7,16], with the immune scores of the low-risk and high-risk patient groups being compared. High-risk patients were found to exhibit a higher immune infiltration score relative to low-risk patients (*p* < 0.01, Figure 8(a)). To evaluate the relationship between the three-gene CRG biosignature developed above and the relative immune cell infiltration of the tumor microenvironment in CRBC patients, we next utilized the CIBERSOFT algorithm to compare high- and low-risk patient immune cell infiltration (Figure 8(b)). This analysis revealed significant differences in immune cell infiltration between groups, including differences in levels of infiltration by resting mast cells, resting dendritic cells, activated CD4 memory T cells, activated dendritic cells, and regulatory T cells (Tregs) (Supplementary Table 9). Subsequent univariate Cox regression analyses of these five immune cell types suggested that they were all significantly associated with patient DFS (*p* < 0.05). Of these, resting dendritic cells, resting mast cells, and Tregs were all associated with a greater risk (HR > 1), whereas resting dendritic cells and activated memory CD4 T cells were protective (HR < 1) (Figure 8(c)).

(A) Differences in scores related to immune cell infiltration between the high-risk group and the low-risk group. (B) Relative proportion of 22 immune cells infiltration in high-risk patients and low-risk patients. (C) Forest plot visualizing for immune cells. Hazard ratios (HR) and confidence intervals (CI) for HRs were calculated according to the Cox models. (D, E) RT-PCR was performed to quantify expression of the three key genes (*KDM7A, MAPT, and PP14571*) in the drug-resistant breast cancer cell lines (MCF-7/Adr and MCF-7/Tax) and their drug-sensitive cell line MCF-7. Bars display as the mean ± SD from three independent experiments. Drug-resistant cell lines compared with drug-sensitive cell line,Statistical significance at the level of null ≥ 0.05, *p < 0.05, **p < 0.01.

### In vitro result validation

The taxane drugs paclitaxel and anthracycline drugs adriamycin are widely used in chemotherapy regimens for the treatment of invasive breast cancer. For in vitro experiments, the drug-resistant MCF-7/Adr and MCF-7/Tax breast cancer cell lines were established respectively by gradually increasing the concentration of adriamycin and taxol (paclitaxel) in the culture medium relative to that used to culture the parental MCF-7 cell line. These cells were then used as drug-resistant models to further validate our prognostic signature. MTT assay results indicated that the The 50% inhibitory concentration (IC50) of paclitaxel for the parental MCF-7 cell line was 0.01 mg/L, while for the drug-resistant MCF-7/Tax cell line the IC50 was 5.27 mg/L. Similarly, the IC50 of adriamycin for the parental MCF-7 cell line was 0.31 mg/L, while that for the drug-resistant MCF-7/Adr cell line was 114.2 mg/L. Subsequently, real-time quantitative PCR (RT-PCR) was performed to quantify the expression of the three key genes (*KDM7A, MAPT*, and *PP14571*) involved in our prognostic signature in these cells. We found that *KDM7A* was upregulated by 1.97-fold, while *MAPT* and *PP14571* were downregulated by 0.65- and 0.83-fold, respectively, in the MCF-7/Tax cell line compared to the MCF-7 control cells. The same gene expression trend was observed in the MCF-7/Adr cell line,with *KDM7A* being upregulated by 1.69-fold, while *MAPT* and *PP14571* were downregulated by 0.48- and 0.77-fold, respectively (Figure 8(d,e)).

## Materials and methods

### Data collection and study design

The GSE25066 dataset and corresponding clinical information were downloaded from the NCBI Gene Expression Omnius (GEO) database (https://www.ncbi.nlm.nih.gov/) [17]. This dataset included 508 total breast cancer patients, of whom 339 (excluding four outlier patients) and 169 were chemoresistant and chemosensitive, respectively. Patients without complete clinical information including age, stage, subtype, and chemoresistance status were excluded from this analysis. Patient clinical characteristics are detailed in Supplementary Table 1, and an overall study flowchart is shown in Figure 1. No ethical oversight was required, as these data were downloaded from a public repository.

### Data preprocessing and CRG identification

Quality control, background correction, normalization, logarithmic conversion, and batch effect removal were performed for all samples with the ‘limma’ R package, after which clinical data were filtered and RNA-seq data were analyzed with the ‘limma’ R package [18]. This same package was also used to identify CRGs by identifying those genes that were differentially expressed between chemoresistant and chemosensitive patients with the following cutoff criteria: FDR < 0.05, absolute value of log2FC > 1 [19].

### Functional enrichment analyses of CRGs

Functional enrichment analyses for CRGs were conducted with the DAVID Bioinformatics Tool (v 6.7) which was used to evaluate Gene Ontology (GO) biological process (GOTERM-BP-FAT) and KEGG pathway enrichment, with the full human whole genome as the background. For GO analyses, significance criteria were *p* < 0.05 and an enrichment score > 1.0 [20], while for KEGG analyses the criteria were *p* < 0.05 and fold enrichment > 2.0 [21]. The Enrichment Map Cytoscape plugin (v 3.4.0) and the ‘goProfiles’ R package were used to visualize the enriched pathways [22].

### Protein-protein interaction network-based establishment and analysis of a co-expression network

An integrated approach was used to assess the functional importance of identified and related genes in the regulation of breast cancer chemoresistance. An initial subset of CRGs was used as a seed gene set. Binary PPINs were then used to expand this initial gene set, incorporating adjacent proteins for each gene signature from this network to develop a larger gene set composed of both CRGs and their putative interaction partners [23]. A weighted gene correlation network for these genes was then conducted based upon the GSE25066 dataset. Expression profiles of genes in this network were inputted into the WGCNA to identify co-expression modules [24]. An adjacency matrix was then established based upon a Pearson’s correlation analysis of all pairs of genes, with the resultant network being used to construct a scale-free co-expression network based on the soft-thresholding parameter *β* capable of enhancing strong correlations between genes and penalizing weak correlations [25]. The adjacency matrix was then used to generate a topological overlap matrix (TOM) measuring the network connectivity of a given gene, as measured by summing its adjacency with other genes within the network [26]. Average linkage hierarchical clustering approaches were then conducted based upon TOM dissimilarity measures with a minimum gene group size of 50 in order to assess genes with similar expression patterns. Gene modules were identified via two approaches. First, gene significance (GS) was defined as GS = lgP in linear regression analyses of gene expression and clinical traits. Module significance (MS) was then defined as the average GS for all genes within a given module [27]. Modules with the top MS values were selected as being most closely related to CRBC patient clinical traits. Modules that were closely correlated with these traits were then selected for downstream analyses.

### Construction of prognostic signature in the training cohort

The impact of individual genes on patient survival was assessed through a series of univariate Cox regression analyses. Survival-related CRGs were those with a *p *< 0.01 in these analyses. A LASSO-penalized Cox regression approach was then used to refine the list of genes identified in these initial analyses in order to develop a prognostic risk signature [28,29]. This analysis was performed with the ‘glmnet’ R package, and was designed to reduce regression coefficients for all variables toward zero, with those of irrelevant gene features being set to zero based upon the regulation weight value (λ). An optimal λ value was selected based upon the minimum cross-validation error in 10-fold cross-validation analyses. A multivariate Cox regression analysis was then performed to evaluate the contributions of individual genes as independent predictors of patient survival outcomes, with the best model ultimately being selected via a stepwise approach. Risk scores were calculated based upon coefficients weighted by the penalized Cox model in the training cohort as follows:

risk score$\;=\overset{n}{\underset{i=1}{\sum }}exp_{i}*\beta_{i}$

Where n was the number of prognostic genes,exp*_i_* was the expression value of genei, and β_i_ was the corresponding multivariate Cox regression coefficient [30,31]. An optimal risk score cutoff was selected with the ‘maxstatr’ R package, with patients being separated into high- and low-risk groups using this cutoff value. Kaplan-Meier curves were then used to assess relationships between risk scores and patient survival. Univariate and multivariate Cox regression analyses were additionally conducted to assess relationships between risk scores and clinical features. Biosignature accuracy and predictive utility were evaluated based upon the area under the ROC curve (AUC) as calculated with the ‘time ROC’ R package.

### Immune cell infiltration analyses

Associations between the tumor microenvironment and CRBC patient risk scores were examined, with the tumor microenvironment being made up of a range of stromal mesenchymal, endothelial, and immune cells together with a range of inflammatory and extracellular matrix molecules [15]. The ESTIMATE R algorithm was utilized to calculate immune scores for samples and to compare levels of immune infiltration between low- and high-risk groups via Wilcoxon tests [32].

The CIBERSORT tool (https://cibersort.stanford.edu/) can be used to gauge approximate cell frequencies within a mixed population of cells based upon gene expression data. For this study, a CIBERSORT analysis was used to assess the relative frequencies of 22 different immune cell types in patient tumor samples [33,34]. For the present analysis, 1000 permutations were used, and samples with a *p *< 0.05 were retained for subsequent analyses. Differences in immune cell infiltration were compared between low- and high-risk patients using Mann-Whitney U tests [32–34].

### Cell culture and treatment

The human breast cancer cell line MCF-7 was obtained from Shanghai Cell Bank of Chinese Academy of Sciences (Shanghai, China) and the paclitaxel-resistant MCF-7/Tax cell line and the adriamycin-resistant MCF-7/Adr cell line were established in vitro by increasing the concentration of paclitaxel (Abcam, ab120143, USA) or adriamycin (Abcam, ab120629, USA) in a stepwise manner using the drug-sensitive parental cell line (MCF-7/Con) as previously reported [35,36]. In order to exclude the impact of the direct effects of these drugs on our study results, MCF-7/Adr and MCF-7/Tax cells were cultured in drug-free medium for at least two weeks before further experiments. Untreated parental MCF-7/Con cells were used as a control. All cells were maintained in RPMI-1640 medium (Gibco, USA), supplemented with 10% heat-inactivated fetal bovine serum, 100 units/mL penicillin, and 100 μg/mL streptomycin in a humidified atmosphere at 37°C under 5% CO_2_.

### Chemosensitivity assay

The level of resistance of the MCF-7/Adr and MCF-7/Tax cells to each drug, with MCF-7/Con cells as a control, was assessed by measureing cellular viability with an MTT assay (Sigma, USA). Briefly, cells in the exponential growth phase were seeded at a density of 5 × 10^4^ cells per well of a 96-well plate in 100 μL for 24 h. The medium was then removed and fresh medium containing different paclitaxel or adriamycin concentrations was added for 72 h. Culture medium was then replaced with 180 μL RPMI-1640 and 20 μL MTT (5 mg MTT/mL) for 4 h, after which supernatants were discarded and formazan crystals were solubilized using 180 μL of DMSO for 10 min while shaking. Absorbance was then assessed at 490 nm on a microplate reader (Bio-Rad 550, USA). The IC50 was estimated with GraphPad Prism 5 for each cell line and each drug.

### Real-time quantitative PCR

Total RNA from the cell lines was extracted using the RNeasy Mini Kit (QIAGEN, Hilden, Germany) according to the provided instructions. All RT-PCR experiments were performed via the SYBR Green method in a CFX-96 Bio-Rad Real-Time PCR (Bio-Rad, USA) instrument. Reactions weres prepared at a final volume of 20 µL using a SYBR Green Mix. The Ct values for each gene were normalized to GAPDH as an internal control, and the 2^−ΔΔCT^ method was used to determine relative gene expression levels. Each reaction was performed in triplicate, and two-way ANOVAs were used to identify differentially expressed genes among these various treatments. The primers used for these reactions are presented in Supplementary Table 10.

### Statistical analysis

Kaplan-Meier curves and two-sided log-rank tests were employed to compare patient survival outcomes using the ‘survival’ R package. The prognostic relevance of the developed multi-gene biosignature was assessed via a multivariate Cox regression approach and through stratified analyses in order to demonstrate that it exhibited prognostic value independent of patient age, stage, subtype, or chemosensitivity status. For these analyses, survival was treated as a dependent variable, with these other factors and the multi-gene signature-derived risk score being treated as independent variables. Hazard ratios (HRs) and 95% confidence intervals (CIs) were calculated. The ‘survivalROC’ R package was used for time-dependent ROC analyses of patient 1-, 3-, and 5-year survival outcomes [37]. AUC values were calculated using these ROC curves. Differences in variables between groups were compared using two-sided Student’s t-tests. R (v.3.5.0) was used for all statistical analyses.

## Discussion

Breast cancer remains a leading cause of mortality among women globally [38], and it is most frequently treated via chemotherapy, even in women that undergo surgery [3]. Chemoresistance remains a primary obstacle to breast cancer treatment, often resulting in disease recurrence and death in affected individuals [2]. As such, we herein sought to design and validate a novel biosignature capable of evaluating CRBC patient prognosis.

To that end, we analyzed an extant CRBC-related dataset, and we then combined PPINs and WGCNA approaches to identify eight co-expression modules, in turn leading to the identification of 698 potentially prognostic CRGs of which three were ultimately used to develop a risk score model. Previously identified single-gene prognostic biomarkers have been found to be insufficiently reliable for use in clinical contexts, potentially because they fail to account for gene-gene interactions. As such, we expanded our primary gene set based upon a binary PPINs sub-class in accordance with prior studies [39], leading to the incorporation of adjacent genes with the goal of improving the prognostic utility of our resultant risk scoring model. We then examined the association between the resultant risk scores and patient outcomes, revealing that high-risk CRBC patients exhibited significant differences in immune cell infiltration, chemotherapeutic efficacy, and multiple signaling pathways relative to low-risk patients.

*KDM7A, MAPT*, and *PP14571* were identified as independent prognostic CRGs that were used to construct our risk model biosignature. All three have previously been linked to breast cancer patient prognosis. *KDM7A* encodes a dual histone demethylase capable of promoting Bcl-2 upregulation and thereby suppressing apoptotic cell death in breast cancer [40]. Estrogen receptor signaling has also been shown to influence the expression of microtubule-associated protein tau (*MAPT*) such that the ER inhibitor fulvestrant can promote *MAPT* downregulation and thereby increase breast cancer cell sensitivity to taxane-based chemotherapy [41]. *PP14571*, also referred to as *AC110619.1*, is a long noncoding RNA that is capable of competitively binding microRNAs and thereby indirectly altering the expression of important genes associated with breast cancer progression [42].For in vitro experiments, paclitaxel- and adriamycin-resistant MCF-7/Tax cell lines were established using the chemosensitive MCF-7 parental cell line, and were used as chemoresistant breast cancer cell model systems with basic drug resistance characteristics. We also found that the expression of *KDM7A* was upregulated in chemoresistant cells (MCF-7/Adr and MCF-7/Tax), while *MAPT* and *PP14571* were downregulated compared with the chemosensitive breast cancer control cell group (MCF-7/Con). The multi-gene risk biosignature established herein also exhibited a high degree of accuracy and predictive value in subsequent analyses. Previous studies have highlighted the value of analyzing the immune cell infiltration of the tumor microenvironment when conducting prognostic assessments of breast cancer patients. Ye et al. screened key prognostic genes in the breast cancer tumor microenvironment by analyzing the immune and stromal scores of tumor samples and also constructed a tumor microenvironment-related prognostic model [43]. Chemoresistance is a major barrier to breast cancer patient treatment that is often associated with disease recurrence and death [7,8,44]. To date, the ESTIMATE and CIBERSORT algorithms have only been rarely utilized to examine CRBC-related immune cell infiltration profiles. As such, we herein analyzed the degree of infiltration by 22 different immune cell populations, revealing that the expression levels of the three CRGs composing our risk score model were associated with CRBC patient immune infiltration scores. This, in turn, suggested that the prognostic value of this scoring model was related to differences in immune responsivity in high- and low-risk CRBC patients. The tumor microenvironment has also been previously linked to the acquisition of chemoresistance in breast cancer [8]. Using the CIBERSORT algorithm, we detected significant differences in the levels of resting mast cells, resting dendritic cells, activated CD4 memory T cells, activated dendritic cells, and Tregs when comparing low- and high-risk CRBC patient samples.

The results of this study are subject to a number of limitations. First, as our results are solely derived from in silico analyses, further in vitro and in vivo validation of the underlying molecular mechanisms will be essential. Second, this was a retrospective study, and future prospective large-scale analyses of CRBC patients will be critical to confirm the prognostic relevance of our multigenic risk biosignature.

In summary, we herein developed an effective prognostic multigenic CRG biosignature capable of predicting CRBC patient outcomes. These differences in survival between high- and low-risk patients were attributable to differences in immune cell infiltration and interactions among a variety of signaling pathways. Overall, this study provides a foundation for future research regarding the pathogenesis and treatment of CRBC.

## Conclusions

In conclusion, we successfully established and conducted the internal validation of a novel prognostic biosignature associated with CRBC patient survival outcomes. The use of this model has the potential to aid in the more accurate risk stratification of CRBC patients and may offer insight into the overall intensity of immune responses within the CRBC-associated tumor microenvironment.

## Supplementary Material

Supplemental Material

## Highlights

- This study was formulated to identify a reliable prognostic biosignature capable of improving prognosis prediction in chemoresistant breast cancer patients
- Multi-gene biosignature was able to predict chemoresistant breast cancer patient outcomes in a manner independent of other patient clinicopathological variables.
- The prognostic biosignature was associated with the infiltration of the tumor microenvironment by multiple immune cell types.
