# Supplementary material for: Identification of a prognostic chemoresistance-related gene signature associated with immune microenvironment in breast cancer
Source: Bioengineered. 2021 Oct 18;12(1):8419–34. doi: 10.1080/21655979.2021.1977768 (PMC8806919; doi:10.1080/21655979.2021.1977768)
Supplement: Supplemental Material [file KBIE_A_1977768_SM3462.zip › supplementary/Supplementary Table 1.doc]

**Supplementary Table 1 Information of datasets used in this study**

| **Parameter** | **Subtype** | **Patients** |
| --- | --- | --- |
| Age (year) | < 50 | 264 |
|  | ≥ 50 | 244 |
| Chemosensitivity | Resistance | 339 |
|  | Sensitive | 169 |
| ERα | Negative | 217 |
|  | Positive | 291 |
| PAM50 | Basal-like | 189 |
|  | HER2+ | 37 |
|  | Luminal A | 160 |
|  | Luminal B | 78 |
|  | Normal | 44 |
| Her 2 | Negative | 485 |
|  | Positive | 6 |
|  | Unknown | 17 |
| T Stage | T0 | 3 |
|  | T1 | 30 |
|  | T2 | 255 |
|  | T3 | 145 |
|  | T4 | 75 |
| N Stage | N0 | 157 |
|  | N1 | 244 |
|  | N2 | 66 |
|  | N3 | 41 |

###### ERα, estrogen receptor alpha; PR, progesterone receptor; HER2, human epithelial growth factor receptor 2; PAM50, prediction analysis of microarray **50**
