# Supplementary material for: Identification of a prognostic chemoresistance-related gene signature associated with immune microenvironment in breast cancer
Source: Bioengineered. 2021 Oct 18;12(1):8419–34. doi: 10.1080/21655979.2021.1977768 (PMC8806919; doi:10.1080/21655979.2021.1977768)
Supplement: Supplemental Material [file KBIE_A_1977768_SM3462.zip › supplementary/Supplementary Table 10.doc]

**Supplementary Table 10 Primer sequences used for real-time PCR in the cell experiment.**

| **Genes** | **Forward / Reverse** | **Primer (5’ to 3’)** | **Amplified fragment size (bp)** | **Tm (℃)** | **Cycles** |
| --- | --- | --- | --- | --- | --- |
| Human KDM7A | Forward | GTCTTCCCAAGTGCCGATG | 101 | 58.5 | 40 |
|  | Reverse | GGGACCATAATAGGGACATCAAATC |  | 59.1 | 40 |
| Human MAPT | Forward | GACAAAAAAGCCAAGACATCCAC | 203 | 59.0 | 40 |
|  | Reverse | ATCTCCTTTGCTCCAGAACTGC |  | 60.6 | 40 |
| Human PP14571 | Forward | AGGAACCTTGAGGACGACGC | 246 | 61.4 | 40 |
|  | Reverse | TCCATTCATGCTGTTGTGCG |  | 60.9 | 40 |
| Human GAPDH | Forward | GGAAGCTTGTCATCAATGGAAATC | 168 | 62.4 | 40 |
|  | Reverse | TGATGACCCTTTTGGCTCCC |  | 62.0 | 40 |
