# Supplementary material for: Identification of a prognostic chemoresistance-related gene signature associated with immune microenvironment in breast cancer
Source: Bioengineered. 2021 Oct 18;12(1):8419–34. doi: 10.1080/21655979.2021.1977768 (PMC8806919; doi:10.1080/21655979.2021.1977768)
Supplement: Supplemental Material [file KBIE_A_1977768_SM3462.zip › supplementary/Supplementary Table 2.doc]

**Supplementary Table2 Differently expressed genes between chemoresistant and non-resistant breast cancer patients.**

| TJP3 | ANO1 | SCUBE2 | CCL8 |
| --- | --- | --- | --- |
| AREG | CDC42BPB | CCR3 | SERPINB4 |
| RABEP1 | MNAT1 | PGR | CLDN3 |
| CD53 | BARD1 | KLRC1 | TRPM4 |
| COTL1 | ACADSB | LYZ | GPR171 |
| CERS4 | MYO6 | SERPINA3 | ALDH3B2 |
| ASTN2 | IGFBP4 | FOXA1 | LAMP3 |
| THEMIS2 | TRBC1 | RAC2 | SCGB1D2 |
| TFF1 | PTPRC | DIXDC1 | KRT15 |
| FAM174B | EFHC1 | SLC4A8 | TMC5 |
| CITED1 | SIAH2 | PLEKHS1 | PIP |
| GREB1 | CLEC4A | SLC16A6 | GSTT2 |
| IRX5 | DNALI1 | AZGP1 | NCF1 |
| NUMA1 | COQ4 | TRAT1 | S100A9 |
| ELOVL2 | PLK2 | RSAD2 | MIR6872 |
| EVI2B | CUEDC1 | CORO1A | CD72 |
| SLC39A6 | GZMB | ARRB2 | TSPAN1 |
| TOX3 | GUSBP3 | GSTM3 | KCNK15 |
| XBP1 | ST3GAL1 | BIN2 | IGF2BP2 |
| LCK | GFRA1 | IL32 | IGFBP2 |
| S100A4 | BCL2A1 | NPL | FCGR3B |
| SERPINA5 | S100A8 | GATA3 | SCGB2A2 |
| SRGN | CLSTN2 | CD52 | MAOB |
| CYTIP | GSTZ1 | PP14571 | ADIRF |
| KRT19 | NAT1 | DAK | PDZK1 |
| IRS1 | LAMA3 | GSTT1 | WIF1 |
| CD151 | FCGR3A | CTSW | DUSP4 |
| ARHGAP25 | ATP13A2 | C6orf211 | CNN1 |
| CD3D | EPN3 | SIX1 | NPY1R |
| TRAC | PEG10 | TBC1D9 | KRT14 |
| ZNF446 | CA12 | APOD | VTCN1 |
| AGR2 | ZNF580 | C1orf21 | MNDA |
| INHBB | TBX3 | BSCL2 | SERHL |
| CD69 | C7orf63 | GYS2 | SLC44A4 |
| DACH1 | ARNT2 | STC2 | AQP9 |
| LILRB2 | MS4A1 | WNT4 | CXCL8 |
| TFF3 | MAPT | DNAJC12 | MGP |
| VAV3 | SELL | LRMP | C8orf44-SGK3 |
| GRP | CCL5 | IFT74 | NKG7 |
| MLPH | BAMBI | STAP1 | CTGF |
| MAPK3 | SYT17 | TDRD12 |  |
