# Supplementary material for: Identification of a prognostic chemoresistance-related gene signature associated with immune microenvironment in breast cancer
Source: Bioengineered. 2021 Oct 18;12(1):8419–34. doi: 10.1080/21655979.2021.1977768 (PMC8806919; doi:10.1080/21655979.2021.1977768)
Supplement: Supplemental Material [file KBIE_A_1977768_SM3462.zip › supplementary/Supplementary Table 3.doc]

**Supplementary Table 3 KEGG pathway analysis based on 163 differentially expressed chemoresistance related genes.**

| **ID** | **Description** | **GeneRatio** | ***p-*value** | **p.adjust** | **Count** |
| --- | --- | --- | --- | --- | --- |
| hsa04666 | Fc gamma R-mediated phagocytosis | 7/80 | 3.76E-05 | 0.004592 | 7 |
| hsa04650 | Natural killer cell mediated cytotoxicity | 8/80 | 4.66E-05 | 0.004592 | 8 |
| hsa04062 | Chemokine signaling pathway | 9/80 | 0.000108 | 0.00706 | 9 |
| hsa00982 | Drug metabolism - cytochrome P450 | 5/80 | 0.000748 | 0.03682 | 5 |
| hsa04662 | B cell receptor signaling pathway | 5/80 | 0.001348 | 0.044272 | 5 |
| hsa05204 | Chemical carcinogenesis | 5/80 | 0.001348 | 0.044272 | 5 |
| hsa04380 | Osteoclast differentiation | 6/80 | 0.001749 | 0.049213 | 6 |
