# Supplementary material for: Identification of a prognostic chemoresistance-related gene signature associated with immune microenvironment in breast cancer
Source: Bioengineered. 2021 Oct 18;12(1):8419–34. doi: 10.1080/21655979.2021.1977768 (PMC8806919; doi:10.1080/21655979.2021.1977768)
Supplement: Supplemental Material [file KBIE_A_1977768_SM3462.zip › supplementary/Supplementary Table 4.doc]

**Supplementary Table 4 Results of GO analysis based on 163 differentially expressed chemoresistance related genes.**

| **ID** | **Description** | **Gene Ratio** | ***p*-value** | **p.adjust** | **Count** |
| --- | --- | --- | --- | --- | --- |
| GO:0060326 | cell chemotaxis | 13/144 | 1.33E-07 | 0.000177 | 13 |
| GO:0006882 | cellular zinc ion homeostasis | 5/144 | 2.47E-07 | 0.000177 | 5 |
| GO:0050851 | antigen receptor-mediated signaling pathway | 12/144 | 2.64E-07 | 0.000177 | 12 |
| GO:0070661 | leukocyte proliferation | 13/144 | 2.82E-07 | 0.000177 | 13 |
| GO:0030595 | leukocyte chemotaxis | 11/144 | 3.53E-07 | 0.000177 | 11 |
| GO:0002768 | immune response-regulating cell surface receptor signaling pathway | 16/144 | 4.32E-07 | 0.000177 | 16 |
| GO:0055069 | zinc ion homeostasis | 5/144 | 4.41E-07 | 0.000177 | 5 |
| GO:0046651 | lymphocyte proliferation | 12/144 | 6.60E-07 | 0.000224 | 12 |
| GO:0032943 | mononuclear cell proliferation | 12/144 | 7.20E-07 | 0.000224 | 12 |
| GO:0002429 | immune response-activating cell surface receptor signaling pathway | 15/144 | 7.93E-07 | 0.000224 | 15 |
| GO:0050900 | leukocyte migration | 16/144 | 1.14E-06 | 0.000291 | 16 |
| GO:0007159 | leukocyte cell-cell adhesion | 13/144 | 2.51E-06 | 0.00059 | 13 |
| GO:0042110 | T cell activation | 15/144 | 2.90E-06 | 0.000612 | 15 |
| GO:0045785 | positive regulation of cell adhesion | 14/144 | 3.04E-06 | 0.000612 | 14 |
| GO:0071621 | granulocyte chemotaxis | 7/144 | 5.06E-06 | 0.000952 | 7 |
| GO:0070663 | regulation of leukocyte proliferation | 10/144 | 9.15E-06 | 0.001613 | 10 |
| GO:0042119 | neutrophil activation | 15/144 | 1.21E-05 | 0.002006 | 15 |
| GO:0042098 | T cell proliferation | 9/144 | 1.33E-05 | 0.00208 | 9 |
| GO:0097530 | granulocyte migration | 7/144 | 1.61E-05 | 0.002386 | 7 |
| GO:0072503 | cellular divalent inorganic cation homeostasis | 13/144 | 1.71E-05 | 0.002411 | 13 |
| GO:0060444 | branching involved in mammary gland duct morphogenesis | 4/144 | 2.62E-05 | 0.003514 | 4 |
| GO:0072507 | divalent inorganic cation homeostasis | 13/144 | 2.88E-05 | 0.003554 | 13 |
| GO:0050857 | positive regulation of antigen receptor-mediated signaling pathway | 4/144 | 3.15E-05 | 0.003554 | 4 |
| GO:0050870 | positive regulation of T cell activation | 9/144 | 3.23E-05 | 0.003554 | 9 |
| GO:0050670 | regulation of lymphocyte proliferation | 9/144 | 3.36E-05 | 0.003554 | 9 |
| GO:0032944 | regulation of mononuclear cell proliferation | 9/144 | 3.50E-05 | 0.003554 | 9 |
| GO:0002791 | regulation of peptide secretion | 14/144 | 3.57E-05 | 0.003554 | 14 |
| GO:0022409 | positive regulation of cell-cell adhesion | 10/144 | 3.62E-05 | 0.003554 | 10 |
| GO:0002696 | positive regulation of leukocyte activation | 11/144 | 3.66E-05 | 0.003554 | 11 |
| GO:0048608 | reproductive structure development | 13/144 | 4.06E-05 | 0.003817 | 13 |
| GO:0061458 | reproductive system development | 13/144 | 4.37E-05 | 0.00397 | 13 |
| GO:0051251 | positive regulation of lymphocyte activation | 10/144 | 4.57E-05 | 0.004024 | 10 |
| GO:0050867 | positive regulation of cell activation | 11/144 | 5.49E-05 | 0.004689 | 11 |
| GO:1903039 | positive regulation of leukocyte cell-cell adhesion | 9/144 | 5.92E-05 | 0.004905 | 9 |
| GO:0006968 | cellular defense response | 5/144 | 6.59E-05 | 0.005155 | 5 |
| GO:0050853 | B cell receptor signaling pathway | 5/144 | 6.59E-05 | 0.005155 | 5 |
| GO:0050671 | positive regulation of lymphocyte proliferation | 7/144 | 6.78E-05 | 0.005155 | 7 |
| GO:0097529 | myeloid leukocyte migration | 8/144 | 7.03E-05 | 0.005155 | 8 |
| GO:0032946 | positive regulation of mononuclear cell proliferation | 7/144 | 7.13E-05 | 0.005155 | 7 |
| GO:0031581 | hemidesmosome assembly | 3/144 | 8.01E-05 | 0.005643 | 3 |
| GO:0010038 | response to metal ion | 11/144 | 8.50E-05 | 0.005834 | 11 |
| GO:0042102 | positive regulation of T cell proliferation | 6/144 | 8.69E-05 | 0.005834 | 6 |
| GO:0060603 | mammary gland duct morphogenesis | 4/144 | 9.33E-05 | 0.006116 | 4 |
| GO:0051249 | regulation of lymphocyte activation | 12/144 | 9.76E-05 | 0.006233 | 12 |
| GO:0006875 | cellular metal ion homeostasis | 13/144 | 0.000103 | 0.006233 | 13 |
| GO:0050852 | T cell receptor signaling pathway | 8/144 | 0.000105 | 0.006233 | 8 |
| GO:0070665 | positive regulation of leukocyte proliferation | 7/144 | 0.000105 | 0.006233 | 7 |
| GO:0070486 | leukocyte aggregation | 3/144 | 0.000106 | 0.006233 | 3 |
| GO:0030593 | neutrophil chemotaxis | 5/144 | 0.00012 | 0.006895 | 5 |
| GO:0002685 | regulation of leukocyte migration | 8/144 | 0.000131 | 0.007282 | 8 |
| GO:0001909 | leukocyte mediated cytotoxicity | 6/144 | 0.000132 | 0.007282 | 6 |
| GO:0043312 | neutrophil degranulation | 13/144 | 0.00015 | 0.008122 | 13 |
| GO:0002283 | neutrophil activation involved in immune response | 13/144 | 0.000159 | 0.008467 | 13 |
| GO:0002694 | regulation of leukocyte activation | 13/144 | 0.000172 | 0.009003 | 13 |
| GO:0048754 | branching morphogenesis of an epithelial tube | 7/144 | 0.000179 | 0.009188 | 7 |
| GO:0050863 | regulation of T cell activation | 10/144 | 0.000192 | 0.009257 | 10 |
| GO:0070670 | response to interleukin-4 | 4/144 | 0.000193 | 0.009257 | 4 |
| GO:0002446 | neutrophil mediated immunity | 13/144 | 0.000198 | 0.009257 | 13 |
| GO:0009636 | response to toxic substance | 13/144 | 0.000198 | 0.009257 | 13 |
| GO:0061180 | mammary gland epithelium development | 5/144 | 0.000203 | 0.009257 | 5 |
| GO:0042129 | regulation of T cell proliferation | 7/144 | 0.000203 | 0.009257 | 7 |
| GO:0002688 | regulation of leukocyte chemotaxis | 6/144 | 0.000204 | 0.009257 | 6 |
| GO:0002070 | epithelial cell maturation | 3/144 | 0.000264 | 0.011807 | 3 |
| GO:0001906 | cell killing | 7/144 | 0.000269 | 0.011859 | 7 |
| GO:1990266 | neutrophil migration | 5/144 | 0.000284 | 0.012327 | 5 |
| GO:0043627 | response to estrogen | 5/144 | 0.000303 | 0.01295 | 5 |
| GO:0022407 | regulation of cell-cell adhesion | 11/144 | 0.000312 | 0.01307 | 11 |
| GO:0046660 | female sex differentiation | 6/144 | 0.000318 | 0.01307 | 6 |
| GO:0050708 | regulation of protein secretion | 12/144 | 0.00032 | 0.01307 | 12 |
| GO:0022612 | gland morphogenesis | 6/144 | 0.000349 | 0.014064 | 6 |
| GO:2001233 | regulation of apoptotic signaling pathway | 11/144 | 0.00037 | 0.014703 | 11 |
| GO:0001911 | negative regulation of leukocyte mediated cytotoxicity | 3/144 | 0.00038 | 0.014812 | 3 |
| GO:0060443 | mammary gland morphogenesis | 4/144 | 0.000387 | 0.014812 | 4 |
| GO:0002793 | positive regulation of peptide secretion | 9/144 | 0.000389 | 0.014812 | 9 |
| GO:0071622 | regulation of granulocyte chemotaxis | 4/144 | 0.000424 | 0.01592 | 4 |
| GO:0002687 | positive regulation of leukocyte migration | 6/144 | 0.000438 | 0.016238 | 6 |
| GO:0045061 | thymic T cell selection | 3/144 | 0.000449 | 0.016423 | 3 |
| GO:0002690 | positive regulation of leukocyte chemotaxis | 5/144 | 0.000491 | 0.017755 | 5 |
| GO:0051897 | positive regulation of protein kinase B signaling | 7/144 | 0.000503 | 0.017944 | 7 |
| GO:0060231 | mesenchymal to epithelial transition | 3/144 | 0.000525 | 0.018487 | 3 |
| GO:0046916 | cellular transition metal ion homeostasis | 5/144 | 0.00055 | 0.019128 | 5 |
| GO:0061138 | morphogenesis of a branching epithelium | 7/144 | 0.000596 | 0.020485 | 7 |
| GO:0030217 | T cell differentiation | 8/144 | 0.000617 | 0.020957 | 8 |
| GO:0032496 | response to lipopolysaccharide | 9/144 | 0.000645 | 0.021387 | 9 |
| GO:1903037 | regulation of leukocyte cell-cell adhesion | 9/144 | 0.000645 | 0.021387 | 9 |
| GO:0031342 | negative regulation of cell killing | 3/144 | 0.0007 | 0.022438 | 3 |
| GO:1901685 | glutathione derivative metabolic process | 3/144 | 0.0007 | 0.022438 | 3 |
| GO:1901687 | glutathione derivative biosynthetic process | 3/144 | 0.0007 | 0.022438 | 3 |
| GO:0046879 | hormone secretion | 9/144 | 0.000744 | 0.023448 | 9 |
| GO:0002703 | regulation of leukocyte mediated immunity | 7/144 | 0.000749 | 0.023448 | 7 |
| GO:0010758 | regulation of macrophage chemotaxis | 3/144 | 0.000801 | 0.024806 | 3 |
| GO:0002706 | regulation of lymphocyte mediated immunity | 6/144 | 0.000811 | 0.024864 | 6 |
| GO:0002700 | regulation of production of molecular mediator of immune response | 6/144 | 0.000876 | 0.02626 | 6 |
| GO:0030879 | mammary gland development | 6/144 | 0.000876 | 0.02626 | 6 |
| GO:0002237 | response to molecule of bacterial origin | 9/144 | 0.000896 | 0.026577 | 9 |
| GO:0006874 | cellular calcium ion homeostasis | 10/144 | 0.000918 | 0.026686 | 10 |
| GO:0002702 | positive regulation of production of molecular mediator of immune response | 5/144 | 0.000923 | 0.026686 | 5 |
| GO:0001763 | morphogenesis of a branching structure | 7/144 | 0.000931 | 0.026686 | 7 |
| GO:0009914 | hormone transport | 9/144 | 0.000937 | 0.026686 | 9 |
| GO:1904950 | negative regulation of establishment of protein localization | 7/144 | 0.000959 | 0.027046 | 7 |
| GO:1901214 | regulation of neuron death | 9/144 | 0.00098 | 0.027361 | 9 |
| GO:0043281 | regulation of cysteine-type endopeptidase activity involved in apoptotic process | 7/144 | 0.00105 | 0.029007 | 7 |
| GO:0007044 | cell-substrate junction assembly | 5/144 | 0.001065 | 0.029137 | 5 |
| GO:0070838 | divalent metal ion transport | 11/144 | 0.001119 | 0.03032 | 11 |
| GO:0090183 | regulation of kidney development | 4/144 | 0.001137 | 0.030483 | 4 |
| GO:0030278 | regulation of ossification | 7/144 | 0.001146 | 0.030483 | 7 |
| GO:0055074 | calcium ion homeostasis | 10/144 | 0.001187 | 0.031269 | 10 |
| GO:0072511 | divalent inorganic cation transport | 11/144 | 0.0012 | 0.031325 | 11 |
| GO:0030098 | lymphocyte differentiation | 9/144 | 0.001274 | 0.032756 | 9 |
| GO:0046545 | development of primary female sexual characteristics | 5/144 | 0.001278 | 0.032756 | 5 |
| GO:0042267 | natural killer cell mediated cytotoxicity | 4/144 | 0.001294 | 0.032858 | 4 |
| GO:0052547 | regulation of peptidase activity | 10/144 | 0.001435 | 0.035883 | 10 |
| GO:0060292 | long-term synaptic depression | 3/144 | 0.001438 | 0.035883 | 3 |
| GO:0002709 | regulation of T cell mediated immunity | 4/144 | 0.001465 | 0.036221 | 4 |
| GO:0043491 | protein kinase B signaling | 8/144 | 0.001478 | 0.036221 | 8 |
| GO:0007548 | sex differentiation | 8/144 | 0.001513 | 0.036763 | 8 |
| GO:0055076 | transition metal ion homeostasis | 5/144 | 0.001588 | 0.038255 | 5 |
| GO:0002228 | natural killer cell mediated immunity | 4/144 | 0.001652 | 0.038805 | 4 |
| GO:0045600 | positive regulation of fat cell differentiation | 4/144 | 0.001652 | 0.038805 | 4 |
| GO:0050854 | regulation of antigen receptor-mediated signaling pathway | 4/144 | 0.001652 | 0.038805 | 4 |
| GO:0001894 | tissue homeostasis | 7/144 | 0.001739 | 0.03989 | 7 |
| GO:0001910 | regulation of leukocyte mediated cytotoxicity | 4/144 | 0.001751 | 0.03989 | 4 |
| GO:0060135 | maternal process involved in female pregnancy | 4/144 | 0.001751 | 0.03989 | 4 |
| GO:0051047 | positive regulation of secretion | 10/144 | 0.001755 | 0.03989 | 10 |
| GO:0002724 | regulation of T cell cytokine production | 3/144 | 0.001939 | 0.043548 | 3 |
| GO:0006816 | calcium ion transport | 10/144 | 0.001952 | 0.043548 | 10 |
| GO:0060675 | ureteric bud morphogenesis | 4/144 | 0.001962 | 0.043548 | 4 |
| GO:0070997 | neuron death | 9/144 | 0.001995 | 0.043938 | 9 |
| GO:0072171 | mesonephric tubule morphogenesis | 4/144 | 0.002074 | 0.045314 | 4 |
| GO:0045444 | fat cell differentiation | 7/144 | 0.002141 | 0.046063 | 7 |
| GO:2000116 | regulation of cysteine-type endopeptidase activity | 7/144 | 0.002141 | 0.046063 | 7 |
| GO:0045137 | development of primary sexual characteristics | 7/144 | 0.002195 | 0.046675 | 7 |
| GO:0048732 | gland development | 10/144 | 0.002205 | 0.046675 | 10 |
| GO:0051235 | maintenance of location | 8/144 | 0.002219 | 0.046675 | 8 |
| GO:0033077 | T cell differentiation in thymus | 4/144 | 0.00231 | 0.047521 | 4 |
| GO:0016055 | Wnt signaling pathway | 11/144 | 0.00232 | 0.047521 | 11 |
| GO:0050673 | epithelial cell proliferation | 10/144 | 0.002321 | 0.047521 | 10 |
| GO:0071353 | cellular response to interleukin-4 | 3/144 | 0.002326 | 0.047521 | 3 |
| GO:0198738 | cell-cell signaling by wnt | 11/144 | 0.002392 | 0.04852 | 11 |
