# Supplementary material for: Identification of a prognostic chemoresistance-related gene signature associated with immune microenvironment in breast cancer
Source: Bioengineered. 2021 Oct 18;12(1):8419–34. doi: 10.1080/21655979.2021.1977768 (PMC8806919; doi:10.1080/21655979.2021.1977768)
Supplement: Supplemental Material [file KBIE_A_1977768_SM3462.zip › supplementary/Supplementary Table 5.doc]

**Supplementary Table 5 The number of genes in each module**

| **Module** | **Number of genes** |
| --- | --- |
| Black | 40 |
| Blue | 360 |
| Brown | 338 |
| Green | 141 |
| Grey | 898 |
| Red | 130 |
| Turquoise | 541 |
| Yellow | 157 |
