# Supplementary material for: Identification of a prognostic chemoresistance-related gene signature associated with immune microenvironment in breast cancer
Source: Bioengineered. 2021 Oct 18;12(1):8419–34. doi: 10.1080/21655979.2021.1977768 (PMC8806919; doi:10.1080/21655979.2021.1977768)
Supplement: Supplemental Material [file KBIE_A_1977768_SM3462.zip › supplementary/Supplementary Table 6.doc]

**Supplementary Table 6 Association between the expression profiles of each gene in most signifificant modules and patients’ survival in the training dataset was evaluated by univariate Cox regression analysis**

| **Gene** | **HR** | **Low 95%CI** | **High 95%CI** | ***p*-value** |
| --- | --- | --- | --- | --- |
| ABAT | 3.640 | 1.828 | 7.245 | 2.35E-04 |
| ACADSB | 1.942 | 1.044 | 3.612 | 3.62E-02 |
| ACTG2 | 0.819 | 0.449 | 1.494 | 5.15E-01 |
| ACTR2 | 0.557 | 0.302 | 1.029 | 6.18E-02 |
| ACTR3 | 0.398 | 0.210 | 0.754 | 4.70E-03 |
| ADAM8 | 0.677 | 0.369 | 1.242 | 2.08E-01 |
| ADCY1 | 2.591 | 1.351 | 4.969 | 4.18E-03 |
| ADCY3 | 0.585 | 0.317 | 1.081 | 8.70E-02 |
| ADCY6 | 2.193 | 1.169 | 4.113 | 1.44E-02 |
| ADCY7 | 0.346 | 0.180 | 0.665 | 1.47E-03 |
| ADCY9 | 2.205 | 1.175 | 4.137 | 1.38E-02 |
| ADIRF | 2.466 | 1.300 | 4.677 | 5.72E-03 |
| ADRB2 | 0.762 | 0.417 | 1.391 | 3.76E-01 |
| AGO2 | 0.609 | 0.332 | 1.118 | 1.10E-01 |
| AGR2 | 2.209 | 1.177 | 4.144 | 1.36E-02 |
| AGTR1 | 1.852 | 0.997 | 3.439 | 5.10E-02 |
| ALDH1A3 | 0.642 | 0.348 | 1.183 | 1.55E-01 |
| ALDH2 | 1.330 | 0.728 | 2.429 | 3.53E-01 |
| ALDH3A2 | 1.835 | 0.994 | 3.389 | 5.24E-02 |
| ALDH3B2 | 2.066 | 1.109 | 3.849 | 2.23E-02 |
| ALOX5 | 0.780 | 0.428 | 1.421 | 4.17E-01 |
| ANO1 | 2.174 | 1.160 | 4.073 | 1.53E-02 |
| ANXA1 | 0.386 | 0.203 | 0.732 | 3.57E-03 |
| AP2B1 | 1.695 | 0.913 | 3.147 | 9.44E-02 |
| APBB1IP | 1.188 | 0.652 | 2.163 | 5.74E-01 |
| APOE | 1.153 | 0.633 | 2.101 | 6.41E-01 |
| APP | 0.599 | 0.327 | 1.100 | 9.83E-02 |
| AQR | 1.438 | 0.787 | 2.627 | 2.38E-01 |
| AR | 2.876 | 1.497 | 5.525 | 1.51E-03 |
| ARAP1 | 0.628 | 0.341 | 1.158 | 1.37E-01 |
| ARAP2 | 0.793 | 0.435 | 1.445 | 4.49E-01 |
| AREG | 1.699 | 0.921 | 3.135 | 9.00E-02 |
| ARHGAP15 | 0.966 | 0.531 | 1.758 | 9.11E-01 |
| ARHGAP17 | 0.663 | 0.361 | 1.215 | 1.84E-01 |
| ARHGAP22 | 0.505 | 0.272 | 0.939 | 3.09E-02 |
| ARHGAP25 | 0.866 | 0.476 | 1.577 | 6.39E-01 |
| ARHGAP32 | 2.738 | 1.425 | 5.260 | 2.51E-03 |
| ARHGAP35 | 2.142 | 1.142 | 4.017 | 1.76E-02 |
| ARHGAP4 | 0.867 | 0.476 | 1.579 | 6.40E-01 |
| ARHGDIB | 0.890 | 0.489 | 1.618 | 7.02E-01 |
| ARHGEF18 | 1.018 | 0.560 | 1.852 | 9.53E-01 |
| ARHGEF4 | 0.718 | 0.393 | 1.313 | 2.82E-01 |
| ARHGEF40 | 0.519 | 0.277 | 0.972 | 4.05E-02 |
| ARHGEF6 | 1.313 | 0.719 | 2.398 | 3.75E-01 |
| ARHGEF9 | 0.960 | 0.528 | 1.746 | 8.93E-01 |
| ARNT2 | 1.555 | 0.848 | 2.853 | 1.54E-01 |
| ARPC1B | 0.589 | 0.319 | 1.087 | 9.03E-02 |
| ARPC2 | 0.369 | 0.194 | 0.701 | 2.33E-03 |
| ARPC5 | 0.797 | 0.437 | 1.452 | 4.59E-01 |
| ARRB1 | 2.376 | 1.254 | 4.500 | 7.94E-03 |
| ARRB2 | 0.927 | 0.510 | 1.687 | 8.05E-01 |
| ART3 | 0.599 | 0.326 | 1.100 | 9.84E-02 |
| ASB13 | 1.762 | 0.949 | 3.272 | 7.28E-02 |
| ASB8 | 2.067 | 1.110 | 3.851 | 2.21E-02 |
| ASTN2 | 2.392 | 1.261 | 4.535 | 7.55E-03 |
| ATF3 | 0.785 | 0.430 | 1.433 | 4.31E-01 |
| ATF4 | 0.408 | 0.217 | 0.768 | 5.51E-03 |
| ATM | 0.901 | 0.494 | 1.643 | 7.33E-01 |
| ATP11A | 0.600 | 0.325 | 1.106 | 1.02E-01 |
| ATP11B | 0.523 | 0.282 | 0.971 | 4.02E-02 |
| ATP8A1 | 0.600 | 0.326 | 1.102 | 9.93E-02 |
| AZGP1 | 1.546 | 0.842 | 2.836 | 1.60E-01 |
| B2M | 0.576 | 0.312 | 1.064 | 7.79E-02 |
| BACH1 | 0.902 | 0.496 | 1.642 | 7.36E-01 |
| BCL2 | 1.518 | 0.828 | 2.785 | 1.77E-01 |
| BCL2A1 | 0.621 | 0.338 | 1.141 | 1.25E-01 |
| BID | 0.614 | 0.334 | 1.127 | 1.15E-01 |
| BIN1 | 0.891 | 0.490 | 1.622 | 7.07E-01 |
| BIN2 | 0.326 | 0.170 | 0.628 | 8.05E-04 |
| BLM | 0.350 | 0.182 | 0.673 | 1.64E-03 |
| BLVRA | 1.988 | 1.069 | 3.695 | 2.99E-02 |
| BMPR1B | 2.164 | 1.155 | 4.055 | 1.60E-02 |
| BTK | 0.570 | 0.309 | 1.052 | 7.21E-02 |
| C1GALT1 | 0.334 | 0.171 | 0.651 | 1.29E-03 |
| C1orf21 | 1.338 | 0.732 | 2.445 | 3.44E-01 |
| C3AR1 | 0.654 | 0.356 | 1.200 | 1.71E-01 |
| C4A | 2.906 | 1.512 | 5.587 | 1.38E-03 |
| C6orf211 | 2.803 | 1.457 | 5.395 | 2.03E-03 |
| C7orf63 | 1.761 | 0.954 | 3.251 | 7.03E-02 |
| CA12 | 3.683 | 1.850 | 7.332 | 2.06E-04 |
| CACNG4 | 1.590 | 0.867 | 2.918 | 1.34E-01 |
| CALU | 0.514 | 0.276 | 0.956 | 3.56E-02 |
| CANT1 | 1.774 | 0.961 | 3.276 | 6.69E-02 |
| CAP1 | 0.409 | 0.216 | 0.776 | 6.18E-03 |
| CAPZA1 | 0.406 | 0.214 | 0.770 | 5.73E-03 |
| CASP8 | 0.951 | 0.523 | 1.730 | 8.70E-01 |
| CBFB | 0.656 | 0.358 | 1.203 | 1.73E-01 |
| CBX4 | 1.405 | 0.768 | 2.571 | 2.69E-01 |
| CCL13 | 0.973 | 0.535 | 1.771 | 9.28E-01 |
| CCL19 | 0.893 | 0.490 | 1.625 | 7.10E-01 |
| CCL20 | 0.458 | 0.242 | 0.867 | 1.64E-02 |
| CCL21 | 1.053 | 0.579 | 1.916 | 8.65E-01 |
| CCL3 | 0.839 | 0.460 | 1.527 | 5.65E-01 |
| CCL4 | 0.830 | 0.456 | 1.512 | 5.44E-01 |
| CCL5 | 0.625 | 0.341 | 1.147 | 1.29E-01 |
| CCND1 | 2.111 | 1.127 | 3.954 | 1.97E-02 |
| CCND2 | 0.730 | 0.399 | 1.334 | 3.06E-01 |
| CCND3 | 0.867 | 0.476 | 1.578 | 6.40E-01 |
| CCNH | 1.244 | 0.682 | 2.266 | 4.76E-01 |
| CCP110 | 0.922 | 0.506 | 1.679 | 7.89E-01 |
| CCR1 | 0.391 | 0.206 | 0.742 | 4.09E-03 |
| CCR2 | 1.289 | 0.703 | 2.364 | 4.12E-01 |
| CCR5 | 0.747 | 0.410 | 1.362 | 3.41E-01 |
| CCR7 | 0.716 | 0.392 | 1.307 | 2.76E-01 |
| CCT2 | 1.288 | 0.707 | 2.347 | 4.08E-01 |
| CD19 | 0.839 | 0.461 | 1.527 | 5.66E-01 |
| CD1C | 0.728 | 0.399 | 1.332 | 3.03E-01 |
| CD1D | 0.800 | 0.439 | 1.459 | 4.67E-01 |
| CD2 | 0.686 | 0.375 | 1.254 | 2.20E-01 |
| CD247 | 0.508 | 0.273 | 0.944 | 3.22E-02 |
| CD28 | 0.721 | 0.395 | 1.317 | 2.87E-01 |
| CD33 | 1.669 | 0.899 | 3.099 | 1.05E-01 |
| CD36 | 1.180 | 0.648 | 2.148 | 5.88E-01 |
| CD3D | 0.731 | 0.401 | 1.335 | 3.08E-01 |
| CD3E | 0.593 | 0.323 | 1.089 | 9.23E-02 |
| CD3G | 1.023 | 0.562 | 1.861 | 9.40E-01 |
| CD4 | 0.920 | 0.505 | 1.675 | 7.84E-01 |
| CD47 | 0.500 | 0.268 | 0.933 | 2.93E-02 |
| CD52 | 0.705 | 0.386 | 1.288 | 2.55E-01 |
| CD53 | 0.453 | 0.243 | 0.846 | 1.29E-02 |
| CD68 | 0.925 | 0.509 | 1.683 | 7.99E-01 |
| CD69 | 0.624 | 0.340 | 1.145 | 1.28E-01 |
| CD72 | 0.956 | 0.525 | 1.739 | 8.82E-01 |
| CD79A | 1.168 | 0.639 | 2.132 | 6.14E-01 |
| CD79B | 0.396 | 0.209 | 0.751 | 4.53E-03 |
| CD86 | 0.530 | 0.286 | 0.981 | 4.32E-02 |
| CD8A | 0.842 | 0.463 | 1.533 | 5.74E-01 |
| CD8B | 0.693 | 0.379 | 1.269 | 2.35E-01 |
| CDC42BPB | 1.355 | 0.741 | 2.476 | 3.24E-01 |
| CEACAM6 | 1.421 | 0.777 | 2.599 | 2.54E-01 |
| CEBPB | 0.359 | 0.187 | 0.690 | 2.12E-03 |
| CECR1 | 1.129 | 0.620 | 2.057 | 6.91E-01 |
| CEP290 | 1.360 | 0.745 | 2.484 | 3.17E-01 |
| CEP57 | 0.628 | 0.343 | 1.152 | 1.33E-01 |
| CERS4 | 2.101 | 1.127 | 3.917 | 1.95E-02 |
| CGA | 1.514 | 0.825 | 2.775 | 1.80E-01 |
| CHI3L1 | 0.305 | 0.156 | 0.597 | 5.29E-04 |
| CHRD | 1.604 | 0.870 | 2.958 | 1.30E-01 |
| CHRM3 | 0.901 | 0.494 | 1.640 | 7.32E-01 |
| CITED1 | 1.391 | 0.762 | 2.541 | 2.83E-01 |
| CLDN1 | 0.906 | 0.497 | 1.650 | 7.47E-01 |
| CLDN3 | 1.916 | 1.032 | 3.559 | 3.95E-02 |
| CLDN4 | 1.047 | 0.575 | 1.904 | 8.82E-01 |
| CLDN7 | 0.892 | 0.490 | 1.624 | 7.09E-01 |
| CLEC4A | 0.750 | 0.411 | 1.370 | 3.49E-01 |
| CLSTN2 | 1.650 | 0.895 | 3.042 | 1.08E-01 |
| CLUAP1 | 1.307 | 0.716 | 2.386 | 3.84E-01 |
| CNN2 | 0.539 | 0.290 | 1.001 | 5.03E-02 |
| CNTRL | 0.664 | 0.362 | 1.218 | 1.86E-01 |
| COPS8 | 1.719 | 0.932 | 3.173 | 8.30E-02 |
| COQ6 | 1.738 | 0.943 | 3.205 | 7.65E-02 |
| COQ7 | 2.458 | 1.297 | 4.658 | 5.81E-03 |
| CORO1A | 0.582 | 0.316 | 1.074 | 8.33E-02 |
| COTL1 | 0.345 | 0.180 | 0.664 | 1.42E-03 |
| CP | 0.579 | 0.314 | 1.068 | 8.04E-02 |
| CSF1R | 0.706 | 0.386 | 1.291 | 2.58E-01 |
| CSK | 0.555 | 0.301 | 1.025 | 5.98E-02 |
| CSNK1A1 | 1.335 | 0.731 | 2.437 | 3.48E-01 |
| CSNK1E | 1.042 | 0.572 | 1.896 | 8.94E-01 |
| CST3 | 2.301 | 1.226 | 4.317 | 9.44E-03 |
| CSTB | 0.428 | 0.226 | 0.810 | 9.11E-03 |
| CTLA4 | 0.937 | 0.515 | 1.705 | 8.31E-01 |
| CTNNA1 | 1.134 | 0.623 | 2.066 | 6.80E-01 |
| CTNND1 | 0.956 | 0.524 | 1.744 | 8.83E-01 |
| CTSC | 1.069 | 0.587 | 1.949 | 8.27E-01 |
| CTSH | 1.240 | 0.679 | 2.265 | 4.83E-01 |
| CTSS | 0.610 | 0.332 | 1.121 | 1.11E-01 |
| CTSW | 1.012 | 0.556 | 1.840 | 9.70E-01 |
| CTTN | 0.805 | 0.441 | 1.470 | 4.81E-01 |
| CUEDC1 | 1.711 | 0.930 | 3.147 | 8.43E-02 |
| CX3CL1 | 0.736 | 0.403 | 1.343 | 3.18E-01 |
| CXCL1 | 0.257 | 0.129 | 0.512 | 1.12E-04 |
| CXCL13 | 0.943 | 0.518 | 1.715 | 8.47E-01 |
| CXCL2 | 0.471 | 0.251 | 0.884 | 1.90E-02 |
| CXCL5 | 0.840 | 0.461 | 1.532 | 5.69E-01 |
| CXCL8 | 0.553 | 0.300 | 1.022 | 5.86E-02 |
| CXCL9 | 1.386 | 0.756 | 2.543 | 2.91E-01 |
| CXCR3 | 1.365 | 0.745 | 2.502 | 3.14E-01 |
| CXCR4 | 0.371 | 0.195 | 0.704 | 2.41E-03 |
| CXCR5 | 1.089 | 0.598 | 1.984 | 7.79E-01 |
| CXCR6 | 1.540 | 0.835 | 2.839 | 1.67E-01 |
| CYBA | 0.702 | 0.384 | 1.282 | 2.49E-01 |
| CYBB | 0.653 | 0.357 | 1.196 | 1.68E-01 |
| CYFIP2 | 1.087 | 0.596 | 1.981 | 7.86E-01 |
| CYTIP | 0.760 | 0.417 | 1.385 | 3.69E-01 |
| DACH1 | 1.743 | 0.945 | 3.216 | 7.54E-02 |
| DAG1 | 1.022 | 0.562 | 1.860 | 9.43E-01 |
| DAPK1 | 0.968 | 0.532 | 1.762 | 9.16E-01 |
| DAPP1 | 0.483 | 0.259 | 0.900 | 2.20E-02 |
| DCTN1 | 0.791 | 0.433 | 1.445 | 4.46E-01 |
| DCTN4 | 2.026 | 1.089 | 3.767 | 2.57E-02 |
| DFFA | 0.489 | 0.261 | 0.915 | 2.54E-02 |
| DIXDC1 | 2.376 | 1.254 | 4.503 | 7.95E-03 |
| DNAAF1 | 0.858 | 0.471 | 1.562 | 6.15E-01 |
| DNAJC12 | 3.675 | 1.848 | 7.307 | 2.06E-04 |
| DNALI1 | 3.206 | 1.643 | 6.255 | 6.34E-04 |
| DOCK2 | 0.759 | 0.416 | 1.383 | 3.67E-01 |
| DOK2 | 0.587 | 0.318 | 1.083 | 8.82E-02 |
| DTX4 | 0.465 | 0.248 | 0.872 | 1.70E-02 |
| DUSP4 | 1.870 | 1.007 | 3.475 | 4.75E-02 |
| DUSP5 | 1.738 | 0.942 | 3.208 | 7.71E-02 |
| DUSP7 | 0.853 | 0.469 | 1.553 | 6.03E-01 |
| DYNC1I1 | 1.231 | 0.674 | 2.249 | 4.99E-01 |
| DYNC2LI1 | 1.917 | 1.036 | 3.548 | 3.82E-02 |
| DYNLRB1 | 1.533 | 0.837 | 2.805 | 1.66E-01 |
| ECM1 | 1.415 | 0.775 | 2.585 | 2.58E-01 |
| EDN1 | 0.717 | 0.391 | 1.315 | 2.82E-01 |
| EFHC1 | 1.453 | 0.794 | 2.658 | 2.26E-01 |
| EFNA1 | 0.703 | 0.383 | 1.289 | 2.54E-01 |
| EGFR | 0.438 | 0.234 | 0.822 | 1.01E-02 |
| ELMO1 | 0.808 | 0.443 | 1.471 | 4.85E-01 |
| ELOVL2 | 1.936 | 1.042 | 3.598 | 3.67E-02 |
| ENPP1 | 2.560 | 1.350 | 4.857 | 4.00E-03 |
| EPB41L1 | 0.866 | 0.476 | 1.577 | 6.39E-01 |
| EPCAM | 0.648 | 0.353 | 1.188 | 1.61E-01 |
| EPN3 | 2.412 | 1.272 | 4.573 | 6.98E-03 |
| ERBB2 | 2.009 | 1.081 | 3.734 | 2.74E-02 |
| ERBB3 | 1.783 | 0.960 | 3.312 | 6.70E-02 |
| ERBB4 | 1.748 | 0.948 | 3.226 | 7.38E-02 |
| ERP44 | 0.583 | 0.316 | 1.075 | 8.42E-02 |
| ESR1 | 4.206 | 2.067 | 8.558 | 7.37E-05 |
| ESRP1 | 0.894 | 0.491 | 1.626 | 7.13E-01 |
| ETS2 | 0.816 | 0.448 | 1.487 | 5.07E-01 |
| EVI2A | 0.584 | 0.318 | 1.074 | 8.37E-02 |
| EVI2B | 0.659 | 0.360 | 1.206 | 1.77E-01 |
| EYA2 | 0.416 | 0.219 | 0.788 | 7.12E-03 |
| FABP5 | 0.487 | 0.262 | 0.905 | 2.29E-02 |
| FAH | 2.018 | 1.085 | 3.753 | 2.66E-02 |
| FAM114A2 | 1.225 | 0.672 | 2.231 | 5.08E-01 |
| FAM13B | 1.346 | 0.737 | 2.460 | 3.34E-01 |
| FAM174B | 2.333 | 1.241 | 4.384 | 8.51E-03 |
| FANCE | 1.026 | 0.564 | 1.865 | 9.34E-01 |
| FBXL5 | 1.692 | 0.917 | 3.120 | 9.22E-02 |
| FBXL7 | 1.271 | 0.698 | 2.314 | 4.34E-01 |
| FBXW4 | 1.666 | 0.904 | 3.071 | 1.02E-01 |
| FCER1G | 0.593 | 0.321 | 1.093 | 9.40E-02 |
| FCGR1A | 1.194 | 0.656 | 2.175 | 5.62E-01 |
| FCGR2A | 1.427 | 0.778 | 2.616 | 2.50E-01 |
| FCGR2B | 0.530 | 0.287 | 0.979 | 4.26E-02 |
| FGD1 | 0.823 | 0.452 | 1.498 | 5.23E-01 |
| FGFR3 | 2.500 | 1.317 | 4.744 | 5.07E-03 |
| FGR | 0.764 | 0.419 | 1.395 | 3.81E-01 |
| FKBP1A | 0.557 | 0.302 | 1.027 | 6.09E-02 |
| FKBP4 | 1.517 | 0.827 | 2.783 | 1.78E-01 |
| FNBP1 | 0.546 | 0.296 | 1.008 | 5.29E-02 |
| FNBP1L | 0.845 | 0.464 | 1.540 | 5.83E-01 |
| FOLR1 | 0.691 | 0.378 | 1.264 | 2.30E-01 |
| FOLR2 | 1.091 | 0.600 | 1.984 | 7.76E-01 |
| FOXA1 | 2.918 | 1.516 | 5.617 | 1.35E-03 |
| FOXO1 | 0.793 | 0.435 | 1.445 | 4.49E-01 |
| FPR3 | 1.105 | 0.607 | 2.014 | 7.43E-01 |
| FSCN1 | 0.326 | 0.167 | 0.636 | 1.00E-03 |
| FTL | 0.632 | 0.345 | 1.161 | 1.39E-01 |
| FYB | 0.523 | 0.282 | 0.973 | 4.05E-02 |
| FYN | 0.773 | 0.424 | 1.409 | 4.00E-01 |
| FZD7 | 0.605 | 0.329 | 1.111 | 1.05E-01 |
| GABBR1 | 1.207 | 0.661 | 2.205 | 5.41E-01 |
| GABBR2 | 0.678 | 0.370 | 1.243 | 2.09E-01 |
| GAK | 1.335 | 0.732 | 2.437 | 3.46E-01 |
| GAL | 0.632 | 0.344 | 1.159 | 1.38E-01 |
| GATA3 | 3.241 | 1.660 | 6.329 | 5.72E-04 |
| GBP2 | 0.914 | 0.503 | 1.663 | 7.69E-01 |
| GDI2 | 0.662 | 0.361 | 1.214 | 1.83E-01 |
| GFRA1 | 2.350 | 1.241 | 4.451 | 8.72E-03 |
| GIPC1 | 0.580 | 0.313 | 1.077 | 8.48E-02 |
| GIT2 | 0.762 | 0.417 | 1.392 | 3.77E-01 |
| GJA1 | 1.597 | 0.871 | 2.928 | 1.30E-01 |
| GLI3 | 1.519 | 0.829 | 2.784 | 1.77E-01 |
| GLIPR1 | 0.603 | 0.328 | 1.109 | 1.04E-01 |
| GMIP | 0.971 | 0.534 | 1.765 | 9.22E-01 |
| GNA11 | 1.058 | 0.581 | 1.925 | 8.53E-01 |
| GNAI1 | 0.673 | 0.367 | 1.235 | 2.01E-01 |
| GNAI2 | 0.724 | 0.395 | 1.327 | 2.96E-01 |
| GNLY | 0.932 | 0.513 | 1.696 | 8.18E-01 |
| GOLM1 | 1.199 | 0.658 | 2.186 | 5.53E-01 |
| GP2 | 1.835 | 0.988 | 3.407 | 5.45E-02 |
| GPC1 | 1.636 | 0.890 | 3.007 | 1.13E-01 |
| GPR171 | 1.080 | 0.593 | 1.966 | 8.02E-01 |
| GPR18 | 0.828 | 0.455 | 1.509 | 5.38E-01 |
| GPR183 | 0.570 | 0.309 | 1.053 | 7.26E-02 |
| GPR65 | 0.507 | 0.273 | 0.943 | 3.19E-02 |
| GPS1 | 1.373 | 0.752 | 2.508 | 3.02E-01 |
| GPSM3 | 0.580 | 0.315 | 1.071 | 8.17E-02 |
| GRAP2 | 0.969 | 0.532 | 1.764 | 9.18E-01 |
| GREB1 | 3.465 | 1.774 | 6.768 | 2.74E-04 |
| GRK5 | 0.770 | 0.422 | 1.405 | 3.94E-01 |
| GSTM3 | 1.923 | 1.035 | 3.572 | 3.86E-02 |
| GTF2F1 | 1.563 | 0.852 | 2.867 | 1.49E-01 |
| GUSB | 1.544 | 0.842 | 2.832 | 1.60E-01 |
| GUSBP3 | 1.903 | 1.025 | 3.534 | 4.16E-02 |
| GYG1 | 0.672 | 0.364 | 1.238 | 2.02E-01 |
| GZMB | 0.613 | 0.333 | 1.126 | 1.15E-01 |
| H2AFV | 1.702 | 0.923 | 3.140 | 8.86E-02 |
| HCK | 1.064 | 0.584 | 1.938 | 8.40E-01 |
| HEBP1 | 1.764 | 0.956 | 3.256 | 6.94E-02 |
| HEBP2 | 0.707 | 0.387 | 1.291 | 2.59E-01 |
| HGD | 1.804 | 0.978 | 3.329 | 5.90E-02 |
| HIF1A | 0.601 | 0.326 | 1.109 | 1.03E-01 |
| HLA.A | 0.637 | 0.347 | 1.170 | 1.46E-01 |
| HLA.B | 0.590 | 0.320 | 1.089 | 9.16E-02 |
| HLA.C | 0.737 | 0.403 | 1.347 | 3.21E-01 |
| HLA.DPA1 | 0.914 | 0.503 | 1.663 | 7.69E-01 |
| HLA.DPB1 | 1.123 | 0.616 | 2.045 | 7.06E-01 |
| HLA.DQA1 | 0.968 | 0.532 | 1.761 | 9.15E-01 |
| HLA.DQB1 | 0.900 | 0.495 | 1.638 | 7.31E-01 |
| HLA.DQB2 | 1.178 | 0.645 | 2.154 | 5.94E-01 |
| HLA.DRA | 0.997 | 0.548 | 1.814 | 9.91E-01 |
| HLA.DRB1 | 0.882 | 0.484 | 1.605 | 6.80E-01 |
| HLA.E | 0.602 | 0.327 | 1.111 | 1.05E-01 |
| HLA.F | 0.661 | 0.360 | 1.212 | 1.81E-01 |
| HLA.G | 0.839 | 0.461 | 1.528 | 5.66E-01 |
| HMGB1 | 0.868 | 0.477 | 1.579 | 6.43E-01 |
| HMHA1 | 1.007 | 0.552 | 1.837 | 9.81E-01 |
| HPSE | 0.744 | 0.408 | 1.359 | 3.36E-01 |
| HRAS | 1.129 | 0.620 | 2.056 | 6.90E-01 |
| HSP90AA1 | 0.763 | 0.418 | 1.393 | 3.78E-01 |
| HSP90AB1 | 1.035 | 0.569 | 1.883 | 9.11E-01 |
| HSPA2 | 1.515 | 0.826 | 2.778 | 1.80E-01 |
| ICAM1 | 0.580 | 0.315 | 1.071 | 8.16E-02 |
| IDO1 | 0.565 | 0.306 | 1.043 | 6.80E-02 |
| IER3 | 2.037 | 1.095 | 3.790 | 2.46E-02 |
| IFIT2 | 0.543 | 0.292 | 1.008 | 5.31E-02 |
| IFITM2 | 0.930 | 0.511 | 1.693 | 8.12E-01 |
| IFT122 | 2.619 | 1.380 | 4.968 | 3.21E-03 |
| IFT140 | 1.645 | 0.892 | 3.032 | 1.11E-01 |
| IFT46 | 3.731 | 1.877 | 7.418 | 1.73E-04 |
| IFT81 | 0.984 | 0.541 | 1.791 | 9.59E-01 |
| IFT88 | 1.654 | 0.900 | 3.040 | 1.05E-01 |
| IGF1R | 1.965 | 1.057 | 3.654 | 3.28E-02 |
| IGF2BP2 | 0.500 | 0.269 | 0.930 | 2.86E-02 |
| IGFBP2 | 1.514 | 0.826 | 2.776 | 1.80E-01 |
| IGFBP4 | 2.293 | 1.222 | 4.301 | 9.73E-03 |
| IGLL5 | 0.687 | 0.376 | 1.256 | 2.22E-01 |
| IKBKB | 1.239 | 0.680 | 2.257 | 4.84E-01 |
| IL12RB2 | 1.172 | 0.644 | 2.135 | 6.03E-01 |
| IL2RA | 0.879 | 0.483 | 1.600 | 6.74E-01 |
| IL2RB | 0.724 | 0.396 | 1.323 | 2.93E-01 |
| IL2RG | 0.755 | 0.414 | 1.377 | 3.60E-01 |
| IL32 | 0.924 | 0.508 | 1.680 | 7.96E-01 |
| IL4R | 0.555 | 0.301 | 1.026 | 6.04E-02 |
| IL6R | 1.121 | 0.616 | 2.041 | 7.09E-01 |
| IL7R | 0.427 | 0.227 | 0.802 | 8.18E-03 |
| INPP5D | 0.480 | 0.256 | 0.900 | 2.21E-02 |
| IRAK1 | 0.475 | 0.253 | 0.889 | 2.01E-02 |
| IRF1 | 0.642 | 0.350 | 1.177 | 1.52E-01 |
| IRF2 | 1.046 | 0.574 | 1.904 | 8.84E-01 |
| IRF6 | 1.584 | 0.864 | 2.906 | 1.37E-01 |
| IRF8 | 0.876 | 0.481 | 1.595 | 6.65E-01 |
| IRS1 | 1.906 | 1.026 | 3.541 | 4.14E-02 |
| IRX5 | 2.161 | 1.161 | 4.025 | 1.51E-02 |
| ISG20 | 0.661 | 0.361 | 1.212 | 1.81E-01 |
| ITGA3 | 1.793 | 0.971 | 3.311 | 6.20E-02 |
| ITGA6 | 0.839 | 0.461 | 1.529 | 5.67E-01 |
| ITGAL | 0.781 | 0.429 | 1.425 | 4.21E-01 |
| ITGAM | 0.878 | 0.482 | 1.597 | 6.69E-01 |
| ITGAX | 0.726 | 0.396 | 1.330 | 3.00E-01 |
| ITGB2 | 0.908 | 0.499 | 1.653 | 7.52E-01 |
| ITK | 0.726 | 0.397 | 1.328 | 2.99E-01 |
| JAK1 | 0.855 | 0.470 | 1.557 | 6.09E-01 |
| JUP | 0.495 | 0.264 | 0.927 | 2.80E-02 |
| KAT2B | 0.927 | 0.510 | 1.687 | 8.04E-01 |
| KCNAB2 | 0.832 | 0.457 | 1.516 | 5.48E-01 |
| KCNK15 | 1.792 | 0.971 | 3.308 | 6.21E-02 |
| KDM7A | 0.269 | 0.135 | 0.535 | 1.82E-04 |
| KHSRP | 0.646 | 0.352 | 1.185 | 1.58E-01 |
| KIF3A | 0.941 | 0.516 | 1.715 | 8.42E-01 |
| KIF3B | 1.382 | 0.757 | 2.523 | 2.93E-01 |
| KIF3C | 0.648 | 0.354 | 1.185 | 1.59E-01 |
| KIT | 0.788 | 0.432 | 1.437 | 4.38E-01 |
| KITLG | 1.730 | 0.937 | 3.192 | 7.96E-02 |
| KLRC1 | 0.853 | 0.468 | 1.553 | 6.02E-01 |
| KLRD1 | 1.173 | 0.642 | 2.143 | 6.04E-01 |
| KRT16 | 0.357 | 0.186 | 0.686 | 1.98E-03 |
| KRT18 | 2.019 | 1.084 | 3.761 | 2.69E-02 |
| KRT19 | 1.446 | 0.790 | 2.645 | 2.32E-01 |
| KRT23 | 0.408 | 0.215 | 0.774 | 6.06E-03 |
| KRT5 | 0.563 | 0.303 | 1.047 | 6.96E-02 |
| KRT6A | 0.452 | 0.241 | 0.847 | 1.32E-02 |
| KRT6B | 0.283 | 0.142 | 0.562 | 3.12E-04 |
| KRT8 | 1.055 | 0.579 | 1.922 | 8.61E-01 |
| KRT81 | 0.950 | 0.522 | 1.728 | 8.67E-01 |
| KRT86 | 1.033 | 0.568 | 1.878 | 9.17E-01 |
| LAIR1 | 0.921 | 0.507 | 1.676 | 7.88E-01 |
| LAMA3 | 1.052 | 0.578 | 1.914 | 8.69E-01 |
| LAMB2 | 1.573 | 0.858 | 2.885 | 1.43E-01 |
| LAMP3 | 0.355 | 0.184 | 0.682 | 1.90E-03 |
| LAPTM5 | 0.493 | 0.265 | 0.918 | 2.59E-02 |
| LAT | 0.828 | 0.455 | 1.508 | 5.38E-01 |
| LCK | 0.709 | 0.388 | 1.295 | 2.63E-01 |
| LCN2 | 0.297 | 0.152 | 0.580 | 3.74E-04 |
| LCP1 | 0.772 | 0.424 | 1.408 | 3.99E-01 |
| LCP2 | 0.521 | 0.281 | 0.969 | 3.93E-02 |
| LIG3 | 1.890 | 1.019 | 3.505 | 4.34E-02 |
| LILRB2 | 1.189 | 0.653 | 2.166 | 5.71E-01 |
| LIPA | 1.799 | 0.969 | 3.339 | 6.28E-02 |
| LMO2 | 0.353 | 0.183 | 0.680 | 1.84E-03 |
| LPAR6 | 1.162 | 0.638 | 2.116 | 6.23E-01 |
| LPXN | 0.547 | 0.296 | 1.011 | 5.42E-02 |
| LRMP | 1.091 | 0.599 | 1.986 | 7.77E-01 |
| LRP2 | 2.289 | 1.209 | 4.334 | 1.10E-02 |
| LRP6 | 0.626 | 0.340 | 1.154 | 1.34E-01 |
| LTBP1 | 0.354 | 0.182 | 0.691 | 2.30E-03 |
| LY6D | 0.637 | 0.347 | 1.169 | 1.45E-01 |
| LY96 | 0.644 | 0.351 | 1.181 | 1.55E-01 |
| LYN | 0.443 | 0.236 | 0.832 | 1.13E-02 |
| LYPD3 | 1.472 | 0.802 | 2.699 | 2.12E-01 |
| LYZ | 0.677 | 0.370 | 1.238 | 2.05E-01 |
| MAGED2 | 2.615 | 1.378 | 4.963 | 3.27E-03 |
| MAN2B1 | 0.958 | 0.527 | 1.743 | 8.89E-01 |
| MAP3K14 | 0.773 | 0.423 | 1.412 | 4.02E-01 |
| MAP4K1 | 1.233 | 0.675 | 2.252 | 4.95E-01 |
| MAPT | 3.282 | 1.680 | 6.411 | 5.03E-04 |
| MATN3 | 1.433 | 0.784 | 2.617 | 2.42E-01 |
| MBTPS1 | 0.870 | 0.477 | 1.587 | 6.50E-01 |
| MCF2L | 1.791 | 0.974 | 3.293 | 6.08E-02 |
| MCL1 | 0.519 | 0.279 | 0.964 | 3.78E-02 |
| MET | 0.403 | 0.212 | 0.764 | 5.40E-03 |
| MFGE8 | 0.716 | 0.392 | 1.308 | 2.77E-01 |
| MIA3 | 1.168 | 0.639 | 2.133 | 6.14E-01 |
| MIR6872 | 2.298 | 1.224 | 4.316 | 9.66E-03 |
| MKNK2 | 1.224 | 0.672 | 2.230 | 5.08E-01 |
| MLEC | 3.179 | 1.630 | 6.199 | 6.88E-04 |
| MLH1 | 2.397 | 1.266 | 4.539 | 7.28E-03 |
| MLPH | 2.555 | 1.344 | 4.857 | 4.22E-03 |
| MMP12 | 0.627 | 0.340 | 1.156 | 1.35E-01 |
| MMP19 | 0.665 | 0.363 | 1.220 | 1.88E-01 |
| MMP7 | 0.427 | 0.227 | 0.803 | 8.23E-03 |
| MMP9 | 0.690 | 0.376 | 1.265 | 2.30E-01 |
| MS4A1 | 0.867 | 0.476 | 1.580 | 6.42E-01 |
| MSLN | 0.510 | 0.275 | 0.948 | 3.32E-02 |
| MUC1 | 1.032 | 0.567 | 1.881 | 9.17E-01 |
| MUC16 | 0.802 | 0.441 | 1.462 | 4.72E-01 |
| MX2 | 0.446 | 0.238 | 0.837 | 1.19E-02 |
| MYB | 1.556 | 0.848 | 2.855 | 1.54E-01 |
| MYD88 | 0.534 | 0.289 | 0.987 | 4.52E-02 |
| MYLIP | 1.658 | 0.903 | 3.043 | 1.03E-01 |
| MYO6 | 1.526 | 0.833 | 2.793 | 1.71E-01 |
| MZT2A | 2.899 | 1.507 | 5.577 | 1.43E-03 |
| NAPA | 2.040 | 1.095 | 3.804 | 2.48E-02 |
| NAT1 | 2.257 | 1.203 | 4.235 | 1.12E-02 |
| NCF1 | 0.885 | 0.487 | 1.610 | 6.90E-01 |
| NCF4 | 0.759 | 0.416 | 1.386 | 3.70E-01 |
| NCK1 | 0.700 | 0.383 | 1.281 | 2.48E-01 |
| NCK2 | 0.677 | 0.370 | 1.239 | 2.06E-01 |
| NCKAP1L | 0.784 | 0.430 | 1.429 | 4.27E-01 |
| NECAP2 | 1.000 | 0.550 | 1.820 | 9.99E-01 |
| NEDD4 | 2.046 | 1.100 | 3.803 | 2.37E-02 |
| NEDD4L | 2.197 | 1.179 | 4.094 | 1.32E-02 |
| NELFCD | 1.192 | 0.655 | 2.171 | 5.65E-01 |
| NET1 | 0.731 | 0.400 | 1.336 | 3.09E-01 |
| NFATC1 | 0.653 | 0.356 | 1.197 | 1.68E-01 |
| NFKB1 | 1.174 | 0.644 | 2.139 | 6.00E-01 |
| NKG7 | 0.728 | 0.399 | 1.330 | 3.02E-01 |
| NME7 | 1.257 | 0.690 | 2.290 | 4.54E-01 |
| NOTCH1 | 0.407 | 0.214 | 0.772 | 5.90E-03 |
| NPC2 | 0.753 | 0.413 | 1.376 | 3.57E-01 |
| NPL | 0.912 | 0.501 | 1.658 | 7.62E-01 |
| NPY1R | 2.083 | 1.112 | 3.902 | 2.20E-02 |
| NR3C1 | 0.582 | 0.316 | 1.073 | 8.29E-02 |
| NRIP1 | 2.667 | 1.405 | 5.062 | 2.70E-03 |
| NUMA1 | 2.296 | 1.230 | 4.288 | 9.08E-03 |
| OCLN | 0.977 | 0.537 | 1.778 | 9.40E-01 |
| OLR1 | 0.667 | 0.363 | 1.224 | 1.91E-01 |
| P2RY10 | 0.685 | 0.373 | 1.256 | 2.21E-01 |
| P2RY14 | 0.362 | 0.188 | 0.694 | 2.24E-03 |
| P2RY2 | 0.990 | 0.543 | 1.802 | 9.73E-01 |
| PADI2 | 0.548 | 0.297 | 1.011 | 5.43E-02 |
| PAK4 | 1.806 | 0.977 | 3.338 | 5.93E-02 |
| PAPD7 | 0.461 | 0.243 | 0.872 | 1.73E-02 |
| PAPSS1 | 0.723 | 0.396 | 1.321 | 2.92E-01 |
| PARN | 0.795 | 0.436 | 1.452 | 4.56E-01 |
| PAXIP1 | 1.474 | 0.803 | 2.703 | 2.10E-01 |
| PCM1 | 1.319 | 0.724 | 2.405 | 3.66E-01 |
| PCYOX1L | 1.353 | 0.741 | 2.471 | 3.25E-01 |
| PDCD1LG2 | 1.323 | 0.724 | 2.417 | 3.62E-01 |
| PDIA6 | 0.801 | 0.439 | 1.459 | 4.67E-01 |
| PDXK | 0.508 | 0.274 | 0.944 | 3.22E-02 |
| PDZK1 | 2.725 | 1.419 | 5.231 | 2.59E-03 |
| PEBP1 | 1.738 | 0.941 | 3.208 | 7.73E-02 |
| PECAM1 | 0.671 | 0.366 | 1.231 | 1.98E-01 |
| PGM1 | 0.675 | 0.369 | 1.235 | 2.02E-01 |
| PGR | 1.338 | 0.733 | 2.443 | 3.43E-01 |
| PICALM | 0.821 | 0.451 | 1.495 | 5.19E-01 |
| PIK3CD | 0.606 | 0.330 | 1.114 | 1.07E-01 |
| PIK3CG | 0.917 | 0.503 | 1.670 | 7.77E-01 |
| PIP | 1.518 | 0.830 | 2.776 | 1.75E-01 |
| PJA2 | 1.591 | 0.867 | 2.922 | 1.34E-01 |
| PLA2G4A | 0.682 | 0.373 | 1.248 | 2.14E-01 |
| PLAC8 | 0.476 | 0.256 | 0.886 | 1.93E-02 |
| PLAUR | 0.398 | 0.210 | 0.756 | 4.85E-03 |
| PLCB4 | 0.860 | 0.472 | 1.566 | 6.22E-01 |
| PLCG2 | 0.524 | 0.283 | 0.969 | 3.93E-02 |
| PLD1 | 0.636 | 0.347 | 1.167 | 1.44E-01 |
| PLK2 | 1.482 | 0.808 | 2.718 | 2.03E-01 |
| PNOC | 0.835 | 0.459 | 1.521 | 5.56E-01 |
| POLD2 | 1.006 | 0.553 | 1.829 | 9.86E-01 |
| POLD4 | 1.605 | 0.873 | 2.951 | 1.28E-01 |
| POLR1C | 0.662 | 0.361 | 1.214 | 1.83E-01 |
| POLR1E | 0.656 | 0.358 | 1.203 | 1.73E-01 |
| POLR2H | 0.611 | 0.331 | 1.126 | 1.14E-01 |
| POLR2I | 0.681 | 0.371 | 1.249 | 2.14E-01 |
| PP14571 | 3.716 | 1.868 | 7.391 | 1.83E-04 |
| PPIE | 0.667 | 0.364 | 1.224 | 1.91E-01 |
| PPP1R3C | 1.672 | 0.910 | 3.073 | 9.79E-02 |
| PPP2CA | 1.061 | 0.582 | 1.936 | 8.46E-01 |
| PPP2R4 | 1.282 | 0.703 | 2.336 | 4.17E-01 |
| PRF1 | 0.642 | 0.350 | 1.179 | 1.53E-01 |
| PRKCB | 0.615 | 0.335 | 1.129 | 1.17E-01 |
| PRKCH | 0.729 | 0.399 | 1.331 | 3.03E-01 |
| PRKCQ | 0.582 | 0.315 | 1.075 | 8.38E-02 |
| PRKCZ | 1.164 | 0.639 | 2.120 | 6.21E-01 |
| PRLR | 3.075 | 1.596 | 5.923 | 7.87E-04 |
| PRNP | 0.481 | 0.258 | 0.896 | 2.12E-02 |
| PRSS23 | 1.418 | 0.776 | 2.589 | 2.56E-01 |
| PSAP | 1.083 | 0.595 | 1.970 | 7.94E-01 |
| PSEN1 | 0.666 | 0.363 | 1.222 | 1.89E-01 |
| PSMB8 | 1.018 | 0.560 | 1.851 | 9.54E-01 |
| PTAFR | 0.996 | 0.547 | 1.811 | 9.88E-01 |
| PTGER3 | 1.435 | 0.786 | 2.622 | 2.40E-01 |
| PTK2 | 0.906 | 0.497 | 1.650 | 7.46E-01 |
| PTK2B | 0.938 | 0.516 | 1.706 | 8.33E-01 |
| PTPN13 | 2.218 | 1.184 | 4.156 | 1.29E-02 |
| PTPN22 | 0.620 | 0.337 | 1.139 | 1.23E-01 |
| PTPN6 | 0.671 | 0.366 | 1.230 | 1.97E-01 |
| PTPN7 | 0.663 | 0.362 | 1.213 | 1.83E-01 |
| PTPRC | 0.589 | 0.320 | 1.083 | 8.87E-02 |
| PTPRCAP | 0.881 | 0.484 | 1.603 | 6.78E-01 |
| PTPRF | 0.777 | 0.426 | 1.419 | 4.12E-01 |
| PTX3 | 0.501 | 0.268 | 0.939 | 3.10E-02 |
| PYCARD | 1.365 | 0.747 | 2.493 | 3.12E-01 |
| QKI | 0.323 | 0.165 | 0.630 | 9.18E-04 |
| RAB14 | 1.997 | 1.067 | 3.740 | 3.06E-02 |
| RAB27A | 0.520 | 0.280 | 0.966 | 3.84E-02 |
| RAB27B | 3.610 | 1.814 | 7.182 | 2.55E-04 |
| RAB5B | 1.051 | 0.578 | 1.912 | 8.70E-01 |
| RAB5C | 1.359 | 0.744 | 2.483 | 3.19E-01 |
| RABEP1 | 2.714 | 1.428 | 5.160 | 2.32E-03 |
| RAC2 | 1.124 | 0.617 | 2.046 | 7.03E-01 |
| RAC3 | 1.059 | 0.582 | 1.927 | 8.51E-01 |
| RAD17 | 1.910 | 1.027 | 3.552 | 4.08E-02 |
| RAD50 | 2.423 | 1.288 | 4.555 | 6.02E-03 |
| RAD51C | 1.526 | 0.832 | 2.801 | 1.72E-01 |
| RAP1A | 0.508 | 0.274 | 0.945 | 3.24E-02 |
| RAP1B | 0.773 | 0.424 | 1.409 | 4.00E-01 |
| RARA | 1.727 | 0.936 | 3.187 | 8.06E-02 |
| RASA1 | 1.446 | 0.789 | 2.651 | 2.33E-01 |
| RBBP6 | 1.118 | 0.615 | 2.033 | 7.15E-01 |
| RBCK1 | 1.250 | 0.686 | 2.276 | 4.67E-01 |
| RBPJ | 0.851 | 0.467 | 1.549 | 5.97E-01 |
| RCN1 | 0.414 | 0.218 | 0.787 | 7.13E-03 |
| REPS2 | 1.113 | 0.611 | 2.026 | 7.27E-01 |
| RET | 1.880 | 1.011 | 3.494 | 4.60E-02 |
| RGS1 | 0.837 | 0.459 | 1.524 | 5.60E-01 |
| RHOA | 0.643 | 0.351 | 1.179 | 1.54E-01 |
| RHOB | 2.000 | 1.074 | 3.723 | 2.88E-02 |
| RHOC | 1.467 | 0.803 | 2.681 | 2.12E-01 |
| RHOD | 0.976 | 0.537 | 1.775 | 9.37E-01 |
| RHOF | 0.738 | 0.404 | 1.346 | 3.22E-01 |
| RHOH | 1.410 | 0.769 | 2.585 | 2.67E-01 |
| RHOQ | 0.731 | 0.400 | 1.335 | 3.08E-01 |
| RHOT1 | 1.515 | 0.825 | 2.780 | 1.80E-01 |
| RHOT2 | 1.487 | 0.813 | 2.720 | 1.98E-01 |
| RNASET2 | 0.706 | 0.386 | 1.290 | 2.58E-01 |
| RNF19B | 0.538 | 0.290 | 1.000 | 4.98E-02 |
| RNF8 | 0.531 | 0.287 | 0.980 | 4.30E-02 |
| RNGTT | 0.642 | 0.349 | 1.178 | 1.52E-01 |
| RPS6KA1 | 1.031 | 0.567 | 1.876 | 9.19E-01 |
| RPS6KA3 | 0.628 | 0.342 | 1.154 | 1.34E-01 |
| RTF1 | 1.856 | 1.000 | 3.446 | 5.01E-02 |
| RUNX3 | 0.546 | 0.295 | 1.008 | 5.32E-02 |
| S100A4 | 0.565 | 0.306 | 1.043 | 6.78E-02 |
| S100B | 1.038 | 0.570 | 1.888 | 9.04E-01 |
| S1PR1 | 0.471 | 0.253 | 0.877 | 1.77E-02 |
| SAMHD1 | 0.640 | 0.348 | 1.174 | 1.49E-01 |
| SAT1 | 0.580 | 0.314 | 1.070 | 8.15E-02 |
| SCAMP1 | 2.938 | 1.528 | 5.651 | 1.23E-03 |
| SCCPDH | 1.957 | 1.052 | 3.641 | 3.40E-02 |
| SCGB1D2 | 1.695 | 0.919 | 3.124 | 9.10E-02 |
| SCGB2A2 | 1.902 | 1.024 | 3.531 | 4.19E-02 |
| SCUBE2 | 3.437 | 1.760 | 6.715 | 3.01E-04 |
| SDC4 | 0.887 | 0.487 | 1.617 | 6.96E-01 |
| SDCBP | 0.498 | 0.268 | 0.925 | 2.73E-02 |
| SEC16A | 1.305 | 0.714 | 2.385 | 3.86E-01 |
| SELPLG | 0.781 | 0.427 | 1.426 | 4.21E-01 |
| SEMA4D | 0.440 | 0.235 | 0.826 | 1.06E-02 |
| SERHL | 1.385 | 0.758 | 2.531 | 2.89E-01 |
| SERPINA3 | 1.508 | 0.822 | 2.765 | 1.85E-01 |
| SERPINA5 | 2.300 | 1.226 | 4.313 | 9.45E-03 |
| SFI1 | 1.044 | 0.574 | 1.901 | 8.88E-01 |
| SFRP1 | 0.473 | 0.252 | 0.887 | 1.97E-02 |
| SH2B1 | 1.614 | 0.878 | 2.969 | 1.23E-01 |
| SH2B3 | 0.449 | 0.239 | 0.842 | 1.26E-02 |
| SH3BP2 | 0.605 | 0.328 | 1.115 | 1.07E-01 |
| SHC1 | 1.232 | 0.676 | 2.244 | 4.96E-01 |
| SIAH2 | 1.826 | 0.986 | 3.380 | 5.55E-02 |
| SIRPA | 0.856 | 0.470 | 1.559 | 6.11E-01 |
| SKP1 | 2.603 | 1.371 | 4.940 | 3.44E-03 |
| SLA | 0.502 | 0.270 | 0.935 | 2.98E-02 |
| SLC16A6 | 2.276 | 1.213 | 4.270 | 1.04E-02 |
| SLC27A2 | 2.254 | 1.200 | 4.232 | 1.15E-02 |
| SLC2A3 | 0.615 | 0.335 | 1.128 | 1.16E-01 |
| SLC2A5 | 0.654 | 0.355 | 1.206 | 1.74E-01 |
| SLC39A6 | 3.355 | 1.716 | 6.560 | 4.02E-04 |
| SLC44A4 | 3.523 | 1.797 | 6.907 | 2.47E-04 |
| SLC4A8 | 1.086 | 0.597 | 1.977 | 7.86E-01 |
| SLC9A3R1 | 3.543 | 1.782 | 7.047 | 3.10E-04 |
| SLPI | 0.622 | 0.338 | 1.147 | 1.29E-01 |
| SMAD1 | 1.422 | 0.778 | 2.598 | 2.53E-01 |
| SMAD3 | 1.889 | 1.016 | 3.509 | 4.43E-02 |
| SMURF2 | 0.716 | 0.391 | 1.311 | 2.79E-01 |
| SOS1 | 0.827 | 0.454 | 1.507 | 5.35E-01 |
| SOSTDC1 | 0.588 | 0.318 | 1.086 | 8.96E-02 |
| SPHK1 | 0.452 | 0.241 | 0.847 | 1.32E-02 |
| SPI1 | 0.536 | 0.289 | 0.996 | 4.85E-02 |
| SPSB1 | 0.410 | 0.216 | 0.777 | 6.27E-03 |
| SRGN | 0.665 | 0.363 | 1.217 | 1.85E-01 |
| ST6GALNAC5 | 0.649 | 0.354 | 1.190 | 1.62E-01 |
| ST8SIA1 | 0.660 | 0.360 | 1.213 | 1.81E-01 |
| STAP1 | 1.036 | 0.570 | 1.884 | 9.08E-01 |
| STAT5A | 0.772 | 0.424 | 1.407 | 3.98E-01 |
| STC2 | 1.985 | 1.067 | 3.690 | 3.03E-02 |
| STK10 | 0.682 | 0.373 | 1.246 | 2.13E-01 |
| STUB1 | 1.952 | 1.049 | 3.630 | 3.46E-02 |
| SUPT4H1 | 1.478 | 0.806 | 2.710 | 2.07E-01 |
| SYK | 0.886 | 0.487 | 1.611 | 6.92E-01 |
| SYT1 | 2.072 | 1.106 | 3.880 | 2.29E-02 |
| SYT17 | 1.226 | 0.672 | 2.233 | 5.07E-01 |
| TAF1D | 0.494 | 0.265 | 0.920 | 2.62E-02 |
| TBC1D9 | 3.624 | 1.822 | 7.208 | 2.42E-04 |
| TBX21 | 0.704 | 0.385 | 1.287 | 2.54E-01 |
| TBX3 | 1.372 | 0.751 | 2.505 | 3.03E-01 |
| TCF3 | 0.749 | 0.410 | 1.368 | 3.47E-01 |
| TFAP2A | 2.046 | 1.090 | 3.839 | 2.59E-02 |
| TFAP2B | 1.372 | 0.750 | 2.507 | 3.05E-01 |
| TFF1 | 1.532 | 0.835 | 2.810 | 1.68E-01 |
| TFF3 | 2.222 | 1.184 | 4.168 | 1.29E-02 |
| TGFBR2 | 0.972 | 0.534 | 1.770 | 9.26E-01 |
| THBS1 | 1.568 | 0.855 | 2.876 | 1.46E-01 |
| THEMIS2 | 0.473 | 0.254 | 0.879 | 1.80E-02 |
| TJP3 | 1.408 | 0.770 | 2.575 | 2.67E-01 |
| TLE3 | 1.019 | 0.560 | 1.855 | 9.51E-01 |
| TLR2 | 0.640 | 0.349 | 1.175 | 1.50E-01 |
| TMC5 | 1.306 | 0.715 | 2.384 | 3.85E-01 |
| TMED10 | 1.528 | 0.834 | 2.802 | 1.70E-01 |
| TMEM132A | 1.019 | 0.560 | 1.853 | 9.51E-01 |
| TMSB4X | 0.585 | 0.317 | 1.078 | 8.57E-02 |
| TNF | 0.556 | 0.301 | 1.027 | 6.07E-02 |
| TNFRSF1B | 0.535 | 0.288 | 0.995 | 4.80E-02 |
| TOR4A | 0.657 | 0.358 | 1.207 | 1.76E-01 |
| TOX3 | 2.737 | 1.424 | 5.260 | 2.53E-03 |
| TP53BP1 | 2.419 | 1.285 | 4.555 | 6.20E-03 |
| TRAC | 0.636 | 0.346 | 1.166 | 1.43E-01 |
| TRAK1 | 1.429 | 0.782 | 2.612 | 2.45E-01 |
| TRAPPC6A | 2.139 | 1.150 | 3.978 | 1.63E-02 |
| TRAT1 | 0.940 | 0.517 | 1.710 | 8.40E-01 |
| TRBC1 | 0.757 | 0.415 | 1.380 | 3.63E-01 |
| TRIM21 | 0.444 | 0.236 | 0.833 | 1.15E-02 |
| TRIM28 | 1.485 | 0.811 | 2.717 | 2.00E-01 |
| TRIM37 | 1.797 | 0.968 | 3.337 | 6.33E-02 |
| TSC2 | 1.553 | 0.849 | 2.840 | 1.53E-01 |
| TSPAN1 | 2.456 | 1.295 | 4.658 | 5.96E-03 |
| TTC30A | 1.290 | 0.706 | 2.355 | 4.07E-01 |
| TUBA1B | 0.528 | 0.284 | 0.981 | 4.32E-02 |
| TUBA4A | 0.507 | 0.273 | 0.943 | 3.18E-02 |
| TUBB4B | 0.959 | 0.527 | 1.745 | 8.92E-01 |
| TYROBP | 0.880 | 0.483 | 1.603 | 6.77E-01 |
| UBE2B | 1.113 | 0.612 | 2.025 | 7.26E-01 |
| UBE2E3 | 0.703 | 0.384 | 1.286 | 2.53E-01 |
| UBE2J1 | 0.591 | 0.321 | 1.089 | 9.19E-02 |
| UBE3A | 2.139 | 1.148 | 3.987 | 1.67E-02 |
| UBE3B | 1.517 | 0.829 | 2.777 | 1.77E-01 |
| UGCG | 2.006 | 1.079 | 3.728 | 2.77E-02 |
| UGP2 | 0.398 | 0.210 | 0.754 | 4.72E-03 |
| UGT8 | 1.058 | 0.581 | 1.928 | 8.53E-01 |
| USP13 | 0.488 | 0.260 | 0.914 | 2.51E-02 |
| USP7 | 1.430 | 0.781 | 2.618 | 2.47E-01 |
| VAMP2 | 1.627 | 0.887 | 2.985 | 1.16E-01 |
| VASP | 0.747 | 0.409 | 1.363 | 3.41E-01 |
| VAV1 | 0.791 | 0.434 | 1.442 | 4.43E-01 |
| VAV3 | 1.909 | 1.028 | 3.548 | 4.08E-02 |
| VCAM1 | 0.667 | 0.364 | 1.222 | 1.90E-01 |
| VNN1 | 0.771 | 0.422 | 1.409 | 3.98E-01 |
| WAS | 1.160 | 0.635 | 2.119 | 6.30E-01 |
| WASF1 | 0.873 | 0.479 | 1.592 | 6.57E-01 |
| WASF3 | 0.765 | 0.420 | 1.393 | 3.81E-01 |
| WDR19 | 1.785 | 0.967 | 3.295 | 6.38E-02 |
| WDR61 | 3.914 | 1.960 | 7.816 | 1.10E-04 |
| WFS1 | 1.808 | 0.979 | 3.337 | 5.84E-02 |
| WNT4 | 1.479 | 0.807 | 2.712 | 2.05E-01 |
| WNT6 | 0.906 | 0.498 | 1.647 | 7.46E-01 |
| WWP1 | 1.892 | 1.018 | 3.515 | 4.37E-02 |
| WWTR1 | 0.616 | 0.334 | 1.137 | 1.21E-01 |
| XBP1 | 3.436 | 1.729 | 6.827 | 4.25E-04 |
| XCL1 | 0.805 | 0.442 | 1.466 | 4.77E-01 |
| YES1 | 0.619 | 0.335 | 1.141 | 1.24E-01 |
| YWHAQ | 0.622 | 0.339 | 1.141 | 1.25E-01 |
| ZAP70 | 0.929 | 0.511 | 1.689 | 8.08E-01 |
| ZNF446 | 1.835 | 0.993 | 3.391 | 5.26E-02 |
