# Supplementary material for: Identification of a prognostic chemoresistance-related gene signature associated with immune microenvironment in breast cancer
Source: Bioengineered. 2021 Oct 18;12(1):8419–34. doi: 10.1080/21655979.2021.1977768 (PMC8806919; doi:10.1080/21655979.2021.1977768)
Supplement: Supplemental Material [file KBIE_A_1977768_SM3462.zip › supplementary/Supplementary Table 7.doc]

**Supplementary Table 7 Gene set enrichment analyses of 201 genes associated with patient disease-free survival within the blue and brown modules**

| **ID** | **Description** | **Set size** | **Enrichment score** | **NES** | ***p***-value | **q-value** |
| --- | --- | --- | --- | --- | --- | --- |
| GO:0009617 | response to bacterium | 17 | 0.616764 | 2.293226 | 0.012739 | 0.362346 |
| GO:0006955 | immune response | 68 | 0.605300 | 3.313209 | 0.025000 | 0.377407 |
| GO:0060326 | cell chemotaxis | 10 | 0.602762 | 1.859637 | 0.021277 | 0.362346 |
| GO:0002376 | immune system process | 81 | 0.573536 | 3.042991 | 0.031250 | 0.460526 |
| GO:0009607 | response to biotic stimulus | 23 | 0.565886 | 2.343787 | 0.007407 | 0.359435 |
| GO:0043207 | response to external biotic stimulus | 23 | 0.565886 | 2.343787 | 0.007407 | 0.359435 |
| GO:0051707 | response to other organism | 23 | 0.565886 | 2.343787 | 0.007407 | 0.359435 |
| GO:0002444 | myeloid leukocyte mediated immunity | 27 | 0.544683 | 2.378214 | 0.008130 | 0.359435 |
| GO:0043299 | leukocyte degranulation | 27 | 0.544683 | 2.378214 | 0.008130 | 0.359435 |
| GO:0001816 | cytokine production | 26 | 0.534876 | 2.284884 | 0.008130 | 0.359435 |
| GO:0002275 | myeloid cell activation involved in immune response | 26 | 0.528618 | 2.258152 | 0.008130 | 0.359435 |
| GO:0002250 | adaptive immune response | 19 | 0.527686 | 2.046988 | 0.006803 | 0.359435 |
| GO:0036230 | granulocyte activation | 26 | 0.527001 | 2.251242 | 0.008130 | 0.359435 |
| GO:0042119 | neutrophil activation | 26 | 0.527001 | 2.251242 | 0.008130 | 0.359435 |
| GO:0038093 | Fc receptor signaling pathway | 11 | 0.525963 | 1.660409 | 0.032407 | 0.466476 |
| GO:0006909 | phagocytosis | 16 | 0.523634 | 1.906656 | 0.017857 | 0.362346 |
| GO:0002274 | myeloid leukocyte activation | 31 | 0.511370 | 2.306860 | 0.010309 | 0.362346 |
| GO:0002283 | neutrophil activation involved in immune response | 25 | 0.509285 | 2.156833 | 0.007752 | 0.359435 |
| GO:0002446 | neutrophil mediated immunity | 25 | 0.509285 | 2.156833 | 0.007752 | 0.359435 |
| GO:0043312 | neutrophil degranulation | 25 | 0.509285 | 2.156833 | 0.007752 | 0.359435 |
| GO:0009987 | cellular process | 181 | 0.496864 | 2.356098 | 0.016667 | 0.362346 |
| GO:0001817 | regulation of cytokine production | 23 | 0.494130 | 2.046589 | 0.007407 | 0.359435 |
| GO:0070661 | leukocyte proliferation | 10 | 0.490764 | 1.514100 | 0.055319 | 0.627333 |
| GO:0045087 | innate immune response | 19 | 0.474390 | 1.840242 | 0.013605 | 0.362346 |
| GO:0002757 | immune response-activating signal transduction | 18 | 0.471104 | 1.792793 | 0.019355 | 0.362346 |
| GO:0002764 | immune response-regulating signaling pathway | 18 | 0.471104 | 1.792793 | 0.019355 | 0.362346 |
| GO:0002429 | immune response-activating cell surface receptor signaling pathway | 15 | 0.466010 | 1.635828 | 0.043210 | 0.524405 |
| GO:0002768 | immune response-regulating cell surface receptor signaling pathway | 15 | 0.466010 | 1.635828 | 0.043210 | 0.524405 |
| GO:0050851 | antigen receptor-mediated signaling pathway | 11 | 0.462839 | 1.461132 | 0.060185 | 0.642267 |
| GO:0002252 | immune effector process | 43 | 0.458395 | 2.233268 | 0.013158 | 0.362346 |
| GO:0044403 | symbiont process | 20 | 0.457891 | 1.833571 | 0.013889 | 0.362346 |
| GO:0044419 | interspecies interaction between organisms | 20 | 0.457891 | 1.833571 | 0.013889 | 0.362346 |
| GO:0002684 | positive regulation of immune system process | 27 | 0.453582 | 1.980447 | 0.016260 | 0.362346 |
| GO:0006952 | defense response | 38 | 0.450253 | 2.173415 | 0.011236 | 0.362346 |
| GO:0002443 | leukocyte mediated immunity | 34 | 0.447934 | 2.058211 | 0.020619 | 0.362346 |
| GO:0002263 | cell activation involved in immune response | 35 | 0.446578 | 2.100499 | 0.020833 | 0.362346 |
| GO:0002366 | leukocyte activation involved in immune response | 35 | 0.446578 | 2.100499 | 0.020833 | 0.362346 |
| GO:0002683 | negative regulation of immune system process | 12 | 0.440602 | 1.448255 | 0.080000 | 0.761781 |
| GO:0050776 | regulation of immune response | 28 | 0.437363 | 1.903088 | 0.017241 | 0.362346 |
| GO:0002682 | regulation of immune system process | 38 | 0.429547 | 2.073466 | 0.011236 | 0.362346 |
| GO:0006281 | DNA repair | 10 | 0.427807 | 1.319868 | 0.131915 | 0.907204 |
| GO:0006887 | exocytosis | 36 | 0.426212 | 1.989169 | 0.022727 | 0.364838 |
| GO:0016032 | viral process | 18 | 0.420991 | 1.602087 | 0.045161 | 0.537547 |
| GO:0002253 | activation of immune response | 19 | 0.419171 | 1.626038 | 0.034014 | 0.471944 |
| GO:0007159 | leukocyte cell-cell adhesion | 13 | 0.408864 | 1.401106 | 0.097297 | 0.824958 |
| GO:0051701 | interaction with host | 10 | 0.406417 | 1.253875 | 0.174468 | 0.967105 |
| GO:0032101 | regulation of response to external stimulus | 21 | 0.405342 | 1.643898 | 0.035714 | 0.471944 |
| GO:0045055 | regulated exocytosis | 34 | 0.394427 | 1.812349 | 0.020619 | 0.362346 |
| GO:0019221 | cytokine-mediated signaling pathway | 29 | 0.393022 | 1.759163 | 0.018182 | 0.362346 |
| GO:0050778 | positive regulation of immune response | 24 | 0.385560 | 1.597164 | 0.057851 | 0.639408 |
| GO:0050863 | regulation of T cell activation | 12 | 0.380319 | 1.250106 | 0.180000 | 0.967105 |
| GO:0045321 | leukocyte activation | 49 | 0.372258 | 1.877954 | 0.016393 | 0.362346 |
| GO:0031347 | regulation of defense response | 16 | 0.370499 | 1.349060 | 0.113095 | 0.883850 |
| GO:1903037 | regulation of leukocyte cell-cell adhesion | 12 | 0.368527 | 1.211347 | 0.215000 | 0.967105 |
| GO:0030162 | regulation of proteolysis | 16 | 0.367605 | 1.338523 | 0.119048 | 0.883850 |
| GO:0034097 | response to cytokine | 40 | 0.363528 | 1.752905 | 0.023810 | 0.368421 |
| GO:0001775 | cell activation | 53 | 0.363064 | 1.825080 | 0.019608 | 0.362346 |
| GO:0030097 | hemopoiesis | 18 | 0.357250 | 1.359520 | 0.109677 | 0.881618 |
| GO:0001659 | temperature homeostasis | 10 | 0.353160 | 1.089568 | 0.331915 | 0.967105 |
| GO:0002460 | adaptive immune response based on somatic recombination of immune receptors built from immunoglobulin superfamily domains | 11 | 0.349131 | 1.102168 | 0.291667 | 0.967105 |
| GO:1902105 | regulation of leukocyte differentiation | 12 | 0.345451 | 1.135494 | 0.265000 | 0.967105 |
| GO:0002697 | regulation of immune effector process | 13 | 0.343037 | 1.175526 | 0.221622 | 0.967105 |
| GO:0010959 | regulation of metal ion transport | 10 | 0.342853 | 1.057766 | 0.361702 | 0.967105 |
| GO:0071345 | cellular response to cytokine stimulus | 39 | 0.337864 | 1.643454 | 0.022989 | 0.364838 |
| GO:0002521 | leukocyte differentiation | 14 | 0.333991 | 1.156341 | 0.222222 | 0.967105 |
| GO:0050727 | regulation of inflammatory response | 13 | 0.333047 | 1.141292 | 0.248649 | 0.967105 |
| GO:0050900 | leukocyte migration | 11 | 0.326013 | 1.029188 | 0.384259 | 0.967105 |
| GO:0048871 | multicellular organismal homeostasis | 20 | 0.324461 | 1.299268 | 0.159722 | 0.967105 |
| GO:0072503 | cellular divalent inorganic cation homeostasis | 10 | 0.323780 | 0.998924 | 0.442553 | 0.967105 |
| GO:0042176 | regulation of protein catabolic process | 12 | 0.323585 | 1.063620 | 0.330000 | 0.967105 |
| GO:0031214 | biomineral tissue development | 10 | 0.320856 | 0.989901 | 0.459574 | 0.967105 |
| GO:0007015 | actin filament organization | 11 | 0.317485 | 1.002265 | 0.421296 | 0.967105 |
| GO:1903706 | regulation of hemopoiesis | 16 | 0.316298 | 1.151705 | 0.250000 | 0.967105 |
| GO:0019932 | second-messenger-mediated signaling | 13 | 0.315659 | 1.081706 | 0.335135 | 0.967105 |
| GO:0002449 | lymphocyte mediated immunity | 10 | 0.314970 | 0.971743 | 0.468085 | 0.967105 |
| GO:0048534 | hematopoietic or lymphoid organ development | 19 | 0.314744 | 1.220949 | 0.170068 | 0.967105 |
| GO:0042110 | T cell activation | 16 | 0.308200 | 1.122217 | 0.267857 | 0.967105 |
| GO:0072507 | divalent inorganic cation homeostasis | 11 | 0.305345 | 0.963940 | 0.481481 | 0.967105 |
| GO:0051129 | negative regulation of cellular component organization | 17 | 0.305034 | 1.134163 | 0.261146 | 0.967105 |
| GO:0001934 | positive regulation of protein phosphorylation | 28 | 0.301957 | 1.313899 | 0.103448 | 0.853721 |
| GO:0006875 | cellular metal ion homeostasis | 13 | 0.298362 | 1.022432 | 0.394595 | 0.967105 |
| GO:0030003 | cellular cation homeostasis | 13 | 0.298362 | 1.022432 | 0.394595 | 0.967105 |
| GO:0007187 | G protein-coupled receptor signaling pathway, coupled to cyclic nucleotide second messenger | 10 | 0.296816 | 0.915736 | 0.548936 | 0.967105 |
| GO:0032147 | activation of protein kinase activity | 11 | 0.290323 | 0.916517 | 0.550926 | 0.967105 |
| GO:0051704 | multi-organism process | 50 | 0.289822 | 1.462636 | 0.035714 | 0.471944 |
| GO:0044265 | cellular macromolecule catabolic process | 20 | 0.288106 | 1.153689 | 0.256944 | 0.967105 |
| GO:0030335 | positive regulation of cell migration | 18 | 0.287337 | 1.093465 | 0.296774 | 0.967105 |
| GO:0040017 | positive regulation of locomotion | 18 | 0.287337 | 1.093465 | 0.296774 | 0.967105 |
| GO:0051272 | positive regulation of cellular component movement | 18 | 0.287337 | 1.093465 | 0.296774 | 0.967105 |
| GO:2000147 | positive regulation of cell motility | 18 | 0.287337 | 1.093465 | 0.296774 | 0.967105 |
| GO:0055065 | metal ion homeostasis | 14 | 0.285868 | 0.989731 | 0.438596 | 0.967105 |
| GO:0055080 | cation homeostasis | 14 | 0.285868 | 0.989731 | 0.438596 | 0.967105 |
| GO:0070371 | ERK1 and ERK2 cascade | 11 | 0.283283 | 0.894294 | 0.592593 | 0.967105 |
| GO:0070372 | regulation of ERK1 and ERK2 cascade | 11 | 0.283283 | 0.894294 | 0.592593 | 0.967105 |
| GO:0002520 | immune system development | 23 | 0.282629 | 1.170594 | 0.222222 | 0.967105 |
| GO:0001503 | ossification | 12 | 0.281081 | 0.923912 | 0.525000 | 0.967105 |
| GO:0018108 | peptidyl-tyrosine phosphorylation | 12 | 0.281081 | 0.923912 | 0.525000 | 0.967105 |
| GO:0018212 | peptidyl-tyrosine modification | 12 | 0.281081 | 0.923912 | 0.525000 | 0.967105 |
| GO:0016192 | vesicle-mediated transport | 57 | 0.280403 | 1.454002 | 0.063830 | 0.666628 |
| GO:0030278 | regulation of ossification | 10 | 0.278075 | 0.857914 | 0.651064 | 0.967105 |
| GO:0050896 | response to stimulus | 150 | 0.277958 | 1.391521 | 0.090909 | 0.798246 |
| GO:0016567 | protein ubiquitination | 15 | 0.269231 | 0.945077 | 0.493827 | 0.967105 |
| GO:0032446 | protein modification by small protein conjugation | 15 | 0.269231 | 0.945077 | 0.493827 | 0.967105 |
| GO:0006897 | endocytosis | 26 | 0.267133 | 1.141140 | 0.219512 | 0.967105 |
| GO:0032940 | secretion by cell | 47 | 0.265140 | 1.329007 | 0.076923 | 0.743927 |
| GO:0002694 | regulation of leukocyte activation | 18 | 0.264807 | 1.007726 | 0.406452 | 0.967105 |
| GO:0009057 | macromolecule catabolic process | 22 | 0.263763 | 1.062564 | 0.296296 | 0.967105 |
| GO:0022407 | regulation of cell-cell adhesion | 15 | 0.262558 | 0.921654 | 0.530864 | 0.967105 |
| GO:0009605 | response to external stimulus | 54 | 0.261054 | 1.302374 | 0.136364 | 0.907547 |
| GO:0050794 | regulation of cellular process | 145 | 0.259820 | 1.425759 | 0.083333 | 0.781499 |
| GO:0051249 | regulation of lymphocyte activation | 17 | 0.258403 | 0.960781 | 0.490446 | 0.967105 |
| GO:0045860 | positive regulation of protein kinase activity | 16 | 0.254144 | 0.925388 | 0.583333 | 0.967105 |
| GO:0006954 | inflammatory response | 22 | 0.253987 | 1.023179 | 0.362963 | 0.967105 |
| GO:0050865 | regulation of cell activation | 20 | 0.253112 | 1.013556 | 0.381944 | 0.967105 |
| GO:0051603 | proteolysis involved in cellular protein catabolic process | 12 | 0.250378 | 0.822992 | 0.720000 | 0.967105 |
| GO:0051247 | positive regulation of protein metabolic process | 39 | 0.249688 | 1.214545 | 0.091954 | 0.798246 |
| GO:0031401 | positive regulation of protein modification process | 32 | 0.248020 | 1.122371 | 0.275510 | 0.967105 |
| GO:0050789 | regulation of biological process | 147 | 0.247973 | 1.275421 | 0.130435 | 0.907104 |
| GO:0070925 | organelle assembly | 13 | 0.241236 | 0.826672 | 0.718919 | 0.967105 |
| GO:0031329 | regulation of cellular catabolic process | 18 | 0.240813 | 0.916417 | 0.567742 | 0.967105 |
| GO:0030029 | actin filament-based process | 18 | 0.240223 | 0.914174 | 0.567742 | 0.967105 |
| GO:0030334 | regulation of cell migration | 22 | 0.239985 | 0.966773 | 0.444444 | 0.967105 |
| GO:0030036 | actin cytoskeleton organization | 16 | 0.238913 | 0.869930 | 0.684524 | 0.967105 |
| GO:0098657 | import into cell | 28 | 0.238798 | 1.039078 | 0.396552 | 0.967105 |
| GO:0007420 | brain development | 16 | 0.237569 | 0.865037 | 0.690476 | 0.967105 |
| GO:0060322 | head development | 16 | 0.237569 | 0.865037 | 0.690476 | 0.967105 |
| GO:0030100 | regulation of endocytosis | 12 | 0.235176 | 0.773022 | 0.785000 | 0.967105 |
| GO:0032970 | regulation of actin filament-based process | 12 | 0.232432 | 0.764004 | 0.800000 | 0.967105 |
| GO:0031346 | positive regulation of cell projection organization | 12 | 0.232241 | 0.763375 | 0.800000 | 0.967105 |
| GO:0030098 | lymphocyte differentiation | 11 | 0.231202 | 0.729881 | 0.842593 | 0.967105 |
| GO:0032956 | regulation of actin cytoskeleton organization | 11 | 0.231183 | 0.729819 | 0.842593 | 0.967105 |
| GO:0032269 | negative regulation of cellular protein metabolic process | 22 | 0.231132 | 0.931109 | 0.555556 | 0.967105 |
| GO:0032270 | positive regulation of cellular protein metabolic process | 37 | 0.228525 | 1.094230 | 0.322581 | 0.967105 |
| GO:0046649 | lymphocyte activation | 24 | 0.228141 | 0.945061 | 0.479339 | 0.967105 |
| GO:0006873 | cellular ion homeostasis | 15 | 0.228020 | 0.800414 | 0.759259 | 0.967105 |
| GO:0043410 | positive regulation of MAPK cascade | 16 | 0.227077 | 0.826833 | 0.744048 | 0.967105 |
| GO:0007166 | cell surface receptor signaling pathway | 78 | 0.226018 | 1.223454 | 0.176471 | 0.967105 |
| GO:0099536 | synaptic signaling | 10 | 0.224714 | 0.693286 | 0.876596 | 0.967105 |
| GO:0099537 | trans-synaptic signaling | 10 | 0.224714 | 0.693286 | 0.876596 | 0.967105 |
| GO:0051270 | regulation of cellular component movement | 23 | 0.223392 | 0.925243 | 0.511111 | 0.967105 |
| GO:2000145 | regulation of cell motility | 23 | 0.223392 | 0.925243 | 0.511111 | 0.967105 |
| GO:0042327 | positive regulation of phosphorylation | 31 | 0.222063 | 1.001756 | 0.463918 | 0.967105 |
| GO:0006935 | chemotaxis | 20 | 0.218244 | 0.873932 | 0.611111 | 0.967105 |
| GO:0042330 | taxis | 20 | 0.218244 | 0.873932 | 0.611111 | 0.967105 |
| GO:0008284 | positive regulation of cell proliferation | 26 | 0.217740 | 0.930141 | 0.544715 | 0.967105 |
| GO:0051248 | negative regulation of protein metabolic process | 26 | 0.217414 | 0.928747 | 0.544715 | 0.967105 |
| GO:0060627 | regulation of vesicle-mediated transport | 17 | 0.217082 | 0.807146 | 0.738854 | 0.967105 |
| GO:0051259 | protein complex oligomerization | 14 | 0.217039 | 0.751433 | 0.842105 | 0.967105 |
| GO:0065007 | biological regulation | 153 | 0.216127 | 1.130274 | 0.450000 | 0.967105 |
| GO:0048518 | positive regulation of biological process | 108 | 0.215720 | 1.170725 | 0.200000 | 0.967105 |
| GO:0010562 | positive regulation of phosphorus metabolic process | 32 | 0.215343 | 0.974497 | 0.489796 | 0.967105 |
| GO:0045937 | positive regulation of phosphate metabolic process | 32 | 0.215343 | 0.974497 | 0.489796 | 0.967105 |
| GO:0030163 | protein catabolic process | 17 | 0.214978 | 0.799323 | 0.745223 | 0.967105 |
| GO:0044087 | regulation of cellular component biogenesis | 20 | 0.214167 | 0.857608 | 0.645833 | 0.967105 |
| GO:0040012 | regulation of locomotion | 24 | 0.213447 | 0.884196 | 0.619835 | 0.967105 |
| GO:1902533 | positive regulation of intracellular signal transduction | 30 | 0.209835 | 0.932222 | 0.546392 | 0.967105 |
| GO:0044257 | cellular protein catabolic process | 13 | 0.209300 | 0.717234 | 0.859459 | 0.967105 |
| GO:0051051 | negative regulation of transport | 16 | 0.207201 | 0.754459 | 0.809524 | 0.967105 |
| GO:0007186 | G protein-coupled receptor signaling pathway | 23 | 0.205351 | 0.850523 | 0.681481 | 0.967105 |
| GO:0007155 | cell adhesion | 33 | 0.205188 | 0.935387 | 0.589474 | 0.967105 |
| GO:0022610 | biological adhesion | 33 | 0.205188 | 0.935387 | 0.589474 | 0.967105 |
| GO:0043269 | regulation of ion transport | 13 | 0.204679 | 0.701398 | 0.870270 | 0.967105 |
| GO:0043933 | protein-containing complex subunit organization | 30 | 0.203593 | 0.904488 | 0.608247 | 0.967105 |
| GO:0046903 | secretion | 51 | 0.203444 | 1.039943 | 0.379310 | 0.967105 |
| GO:0065003 | protein-containing complex assembly | 29 | 0.202381 | 0.905856 | 0.609091 | 0.967105 |
| GO:0007010 | cytoskeleton organization | 24 | 0.196532 | 0.814123 | 0.760331 | 0.967105 |
| GO:0009894 | regulation of catabolic process | 21 | 0.195069 | 0.791119 | 0.757143 | 0.967105 |
| GO:0098609 | cell-cell adhesion | 21 | 0.194430 | 0.788525 | 0.757143 | 0.967105 |
| GO:0051241 | negative regulation of multicellular organismal process | 31 | 0.191874 | 0.865570 | 0.680412 | 0.967105 |
| GO:0048584 | positive regulation of response to stimulus | 64 | 0.191510 | 1.013071 | 0.425000 | 0.967105 |
| GO:0042127 | regulation of cell proliferation | 40 | 0.190998 | 0.920978 | 0.654762 | 0.967105 |
| GO:0032268 | regulation of cellular protein metabolic process | 54 | 0.189605 | 0.945919 | 0.568182 | 0.967105 |
| GO:0043588 | skin development | 11 | 0.188172 | 0.594039 | 0.976852 | 0.967105 |
| GO:0007165 | signal transduction | 114 | 0.187944 | 0.991220 | 0.500000 | 0.967105 |
| GO:0044089 | positive regulation of cellular component biogenesis | 15 | 0.186813 | 0.655767 | 0.925926 | 0.967105 |
| GO:0010604 | positive regulation of macromolecule metabolic process | 72 | 0.185863 | 1.019996 | 0.406250 | 0.967105 |
| GO:0097435 | supramolecular fiber organization | 14 | 0.185792 | 0.643249 | 0.929825 | 0.967105 |
| GO:0051246 | regulation of protein metabolic process | 60 | 0.185178 | 0.951370 | 0.625000 | 0.967105 |
| GO:0051130 | positive regulation of cellular component organization | 38 | 0.184738 | 0.891747 | 0.651685 | 0.967105 |
| GO:0030031 | cell projection assembly | 13 | 0.182722 | 0.626157 | 0.940541 | 0.967105 |
| GO:0120031 | plasma membrane bounded cell projection assembly | 13 | 0.182722 | 0.626157 | 0.940541 | 0.967105 |
| GO:0044085 | cellular component biogenesis | 48 | 0.181208 | 0.912276 | 0.661290 | 0.967105 |
| GO:0051716 | cellular response to stimulus | 129 | 0.180959 | 0.970682 | 0.785714 | 0.967105 |
| GO:0001932 | regulation of protein phosphorylation | 36 | 0.178955 | 0.835198 | 0.761364 | 0.967105 |
| GO:0022607 | cellular component assembly | 46 | 0.178808 | 0.898165 | 0.724638 | 0.967105 |
| GO:0048870 | cell motility | 34 | 0.177994 | 0.817862 | 0.783505 | 0.967105 |
| GO:0051674 | localization of cell | 34 | 0.177994 | 0.817862 | 0.783505 | 0.967105 |
| GO:0009893 | positive regulation of metabolic process | 78 | 0.176304 | 0.954344 | 0.558824 | 0.967105 |
| GO:0045859 | regulation of protein kinase activity | 23 | 0.175441 | 0.726641 | 0.888889 | 0.967105 |
| GO:0006950 | response to stress | 78 | 0.174062 | 0.942211 | 0.558824 | 0.967105 |
| GO:0016477 | cell migration | 31 | 0.172933 | 0.780125 | 0.804124 | 0.967105 |
| GO:0007154 | cell communication | 117 | 0.170625 | 0.918423 | 0.611111 | 0.967105 |
| GO:0023052 | signaling | 117 | 0.170625 | 0.918423 | 0.611111 | 0.967105 |
| GO:0043085 | positive regulation of catalytic activity | 35 | 0.166667 | 0.783924 | 0.833333 | 0.967105 |
| GO:0040011 | locomotion | 41 | 0.165892 | 0.793117 | 0.790698 | 0.967105 |
| GO:0048522 | positive regulation of cellular process | 101 | 0.163312 | 0.893678 | 0.764706 | 0.967105 |
| GO:0051240 | positive regulation of multicellular organismal process | 46 | 0.162899 | 0.818251 | 0.840580 | 0.967105 |
| GO:0043408 | regulation of MAPK cascade | 22 | 0.158905 | 0.640146 | 0.948148 | 0.967105 |
| GO:0051347 | positive regulation of transferase activity | 23 | 0.155172 | 0.642693 | 0.962963 | 0.967105 |
| GO:0033674 | positive regulation of kinase activity | 22 | 0.154286 | 0.621536 | 0.970370 | 0.967105 |
| GO:0051239 | regulation of multicellular organismal process | 76 | 0.153617 | 0.826920 | 0.750000 | 0.967105 |
| GO:0010033 | response to organic substance | 82 | 0.152138 | 0.810199 | 0.838710 | 0.967105 |
| GO:0090407 | organophosphate biosynthetic process | 13 | 0.142386 | 0.487932 | 1.000000 | 0.967105 |
| GO:0071310 | cellular response to organic substance | 76 | 0.141628 | 0.762383 | 0.875000 | 0.967105 |
| GO:0051173 | positive regulation of nitrogen compound metabolic process | 64 | 0.140927 | 0.745494 | 0.900000 | 0.967105 |
| GO:1901575 | organic substance catabolic process | 33 | 0.129936 | 0.592339 | 0.989474 | 0.967105 |
| GO:0019538 | protein metabolic process | 85 | 0.124291 | 0.675047 | 1.000000 | 0.967105 |
| GO:0031325 | positive regulation of cellular metabolic process | 68 | 0.122154 | 0.668630 | 0.950000 | 0.967105 |
| GO:0044267 | cellular protein metabolic process | 80 | 0.111111 | 0.599204 | 1.000000 | 0.967105 |
| GO:0035556 | intracellular signal transduction | 66 | 0.109681 | 0.571336 | 1.000000 | 0.967105 |
| GO:0043170 | macromolecule metabolic process | 111 | 0.103179 | 0.550866 | 1.000000 | 0.967105 |
| GO:0044260 | cellular macromolecule metabolic process | 106 | 0.090370 | 0.501600 | 1.000000 | 0.967105 |
| GO:0006807 | nitrogen compound metabolic process | 119 | -0.100917 | -0.349764 | 1.000000 | 0.967105 |
| GO:0051171 | regulation of nitrogen compound metabolic process | 90 | -0.114004 | -0.393542 | 1.000000 | 0.967105 |
| GO:0035690 | cellular response to drug | 18 | -0.120198 | -0.336558 | 1.000000 | 0.967105 |
| GO:1901564 | organonitrogen compound metabolic process | 96 | -0.120726 | -0.418168 | 1.000000 | 0.967105 |
| GO:0051128 | regulation of cellular component organization | 58 | -0.122137 | -0.408761 | 0.995825 | 0.967105 |
| GO:0044237 | cellular metabolic process | 127 | -0.122821 | -0.424594 | 0.998986 | 0.967105 |
| GO:0016043 | cellular component organization | 97 | -0.123089 | -0.427932 | 0.998982 | 0.967105 |
| GO:0071840 | cellular component organization or biogenesis | 97 | -0.123089 | -0.427932 | 0.998982 | 0.967105 |
| GO:0006996 | organelle organization | 50 | -0.123988 | -0.407287 | 0.998943 | 0.967105 |
| GO:0006464 | cellular protein modification process | 72 | -0.125447 | -0.428874 | 0.996907 | 0.967105 |
| GO:0036211 | protein modification process | 72 | -0.125447 | -0.428874 | 0.996907 | 0.967105 |
| GO:0043412 | macromolecule modification | 72 | -0.125447 | -0.428874 | 0.996907 | 0.967105 |
| GO:0009892 | negative regulation of metabolic process | 55 | -0.126144 | -0.420565 | 0.998959 | 0.967105 |
| GO:0012501 | programmed cell death | 56 | -0.134304 | -0.448877 | 0.995833 | 0.967105 |
| GO:0050790 | regulation of catalytic activity | 51 | -0.135420 | -0.444967 | 0.996822 | 0.967105 |
| GO:0031323 | regulation of cellular metabolic process | 98 | -0.136486 | -0.474499 | 0.997967 | 0.967105 |
| GO:0060255 | regulation of macromolecule metabolic process | 94 | -0.137175 | -0.474672 | 0.996948 | 0.967105 |
| GO:0031399 | regulation of protein modification process | 43 | -0.140790 | -0.454582 | 0.995680 | 0.967105 |
| GO:0044093 | positive regulation of molecular function | 47 | -0.145114 | -0.475307 | 0.994664 | 0.967105 |
| GO:0071704 | organic substance metabolic process | 129 | -0.148953 | -0.513761 | 0.993927 | 0.967105 |
| GO:0044238 | primary metabolic process | 122 | -0.149184 | -0.514795 | 0.993896 | 0.967105 |
| GO:0031324 | negative regulation of cellular metabolic process | 50 | -0.150997 | -0.496008 | 0.990486 | 0.967105 |
| GO:0019222 | regulation of metabolic process | 100 | -0.151688 | -0.526180 | 0.994919 | 0.967105 |
| GO:1901565 | organonitrogen compound catabolic process | 22 | -0.151987 | -0.442670 | 0.996540 | 0.967105 |
| GO:0042592 | homeostatic process | 47 | -0.152436 | -0.499288 | 0.994664 | 0.967105 |
| GO:0008152 | metabolic process | 133 | -0.153944 | -0.528904 | 0.992893 | 0.967105 |
| GO:0010605 | negative regulation of macromolecule metabolic process | 51 | -0.154932 | -0.509082 | 0.992585 | 0.967105 |
| GO:0009056 | catabolic process | 39 | -0.156209 | -0.497532 | 0.985792 | 0.967105 |
| GO:0080090 | regulation of primary metabolic process | 93 | -0.157140 | -0.543004 | 0.985714 | 0.967105 |
| GO:0032501 | multicellular organismal process | 116 | -0.157605 | -0.545949 | 0.989858 | 0.967105 |
| GO:0051172 | negative regulation of nitrogen compound metabolic process | 49 | -0.159187 | -0.522853 | 0.987248 | 0.967105 |
| GO:0008219 | cell death | 58 | -0.159361 | -0.533341 | 0.988518 | 0.967105 |
| GO:0006974 | cellular response to DNA damage stimulus | 18 | -0.159899 | -0.447722 | 0.994097 | 0.967105 |
| GO:0018193 | peptidyl-amino acid modification | 18 | -0.160746 | -0.450095 | 0.994097 | 0.967105 |
| GO:0051336 | regulation of hydrolase activity | 28 | -0.160788 | -0.486908 | 0.988713 | 0.967105 |
| GO:0080134 | regulation of response to stress | 33 | -0.161588 | -0.503030 | 0.986770 | 0.967105 |
| GO:0033043 | regulation of organelle organization | 25 | -0.162512 | -0.484965 | 0.983963 | 0.967105 |
| GO:0051090 | regulation of DNA-binding transcription factor activity | 14 | -0.163934 | -0.439057 | 0.997593 | 0.967105 |
| GO:0043069 | negative regulation of programmed cell death | 28 | -0.163963 | -0.496525 | 0.988713 | 0.967105 |
| GO:0031344 | regulation of cell projection organization | 20 | -0.165762 | -0.475188 | 0.994172 | 0.967105 |
| GO:0051091 | positive regulation of DNA-binding transcription factor activity | 11 | -0.166667 | -0.418635 | 0.996183 | 0.967105 |
| GO:0048583 | regulation of response to stimulus | 92 | -0.167575 | -0.578299 | 0.981576 | 0.967105 |
| GO:0006468 | protein phosphorylation | 41 | -0.168012 | -0.536632 | 0.983624 | 0.967105 |
| GO:2000026 | regulation of multicellular organismal development | 51 | -0.168891 | -0.554949 | 0.987288 | 0.967105 |
| GO:0051345 | positive regulation of hydrolase activity | 19 | -0.170669 | -0.483131 | 0.992982 | 0.967105 |
| GO:1902531 | regulation of intracellular signal transduction | 49 | -0.170778 | -0.560925 | 0.979809 | 0.967105 |
| GO:0046483 | heterocycle metabolic process | 71 | -0.172766 | -0.589229 | 0.964803 | 0.967105 |
| GO:0006793 | phosphorus metabolic process | 55 | -0.174873 | -0.583030 | 0.975026 | 0.967105 |
| GO:0006796 | phosphate-containing compound metabolic process | 55 | -0.174873 | -0.583030 | 0.975026 | 0.967105 |
| GO:0009889 | regulation of biosynthetic process | 65 | -0.177022 | -0.599610 | 0.964840 | 0.967105 |
| GO:0044248 | cellular catabolic process | 36 | -0.177971 | -0.561246 | 0.968271 | 0.967105 |
| GO:0090304 | nucleic acid metabolic process | 64 | -0.178881 | -0.603934 | 0.965696 | 0.967105 |
| GO:0019220 | regulation of phosphate metabolic process | 41 | -0.179073 | -0.571961 | 0.973799 | 0.967105 |
| GO:0051174 | regulation of phosphorus metabolic process | 41 | -0.179073 | -0.571961 | 0.973799 | 0.967105 |
| GO:0006139 | nucleobase-containing compound metabolic process | 69 | -0.179397 | -0.612063 | 0.955487 | 0.967105 |
| GO:0043066 | negative regulation of apoptotic process | 26 | -0.180779 | -0.542210 | 0.955631 | 0.967105 |
| GO:0070887 | cellular response to chemical stimulus | 86 | -0.181120 | -0.622057 | 0.967314 | 0.967105 |
| GO:0007264 | small GTPase mediated signal transduction | 11 | -0.181400 | -0.455641 | 0.996183 | 0.967105 |
| GO:0048519 | negative regulation of biological process | 92 | -0.181893 | -0.627713 | 0.966223 | 0.967105 |
| GO:0006915 | apoptotic process | 49 | -0.182428 | -0.599187 | 0.965994 | 0.967105 |
| GO:0006259 | DNA metabolic process | 16 | -0.182518 | -0.497310 | 0.984412 | 0.967105 |
| GO:0061024 | membrane organization | 21 | -0.183225 | -0.531674 | 0.975638 | 0.967105 |
| GO:0065009 | regulation of molecular function | 69 | -0.183264 | -0.625255 | 0.951346 | 0.967105 |
| GO:0032386 | regulation of intracellular transport | 12 | -0.183385 | -0.471657 | 0.988778 | 0.967105 |
| GO:0044271 | cellular nitrogen compound biosynthetic process | 65 | -0.186600 | -0.632054 | 0.952430 | 0.967105 |
| GO:0042325 | regulation of phosphorylation | 40 | -0.186853 | -0.595622 | 0.968410 | 0.967105 |
| GO:0019219 | regulation of nucleobase-containing compound metabolic process | 60 | -0.187178 | -0.627128 | 0.957023 | 0.967105 |
| GO:0048523 | negative regulation of cellular process | 86 | -0.187545 | -0.644123 | 0.955056 | 0.967105 |
| GO:0030154 | cell differentiation | 79 | -0.187727 | -0.640339 | 0.963843 | 0.967105 |
| GO:0048869 | cellular developmental process | 79 | -0.187727 | -0.640339 | 0.963843 | 0.967105 |
| GO:0010638 | positive regulation of organelle organization | 20 | -0.190749 | -0.546819 | 0.963869 | 0.967105 |
| GO:0044092 | negative regulation of molecular function | 26 | -0.191942 | -0.575690 | 0.946530 | 0.967105 |
| GO:0045595 | regulation of cell differentiation | 43 | -0.192411 | -0.621257 | 0.957883 | 0.967105 |
| GO:0016310 | phosphorylation | 45 | -0.193478 | -0.629518 | 0.945396 | 0.967105 |
| GO:0010467 | gene expression | 66 | -0.193525 | -0.657413 | 0.933816 | 0.967105 |
| GO:0006508 | proteolysis | 28 | -0.194578 | -0.589235 | 0.950339 | 0.967105 |
| GO:0120035 | regulation of plasma membrane bounded cell projection organization | 19 | -0.194652 | -0.551021 | 0.974269 | 0.967105 |
| GO:0034641 | cellular nitrogen compound metabolic process | 76 | -0.194680 | -0.664836 | 0.940206 | 0.967105 |
| GO:0044057 | regulation of system process | 10 | -0.196264 | -0.478100 | 0.994785 | 0.967105 |
| GO:0006979 | response to oxidative stress | 12 | -0.196698 | -0.505897 | 0.982544 | 0.967105 |
| GO:0010468 | regulation of gene expression | 65 | -0.197712 | -0.669691 | 0.921406 | 0.967105 |
| GO:0043087 | regulation of GTPase activity | 13 | -0.197743 | -0.519902 | 0.985312 | 0.967105 |
| GO:0043603 | cellular amide metabolic process | 11 | -0.197991 | -0.497315 | 0.982188 | 0.967105 |
| GO:0031326 | regulation of cellular biosynthetic process | 63 | -0.198170 | -0.668052 | 0.921794 | 0.967105 |
| GO:0030030 | cell projection organization | 36 | -0.199473 | -0.629055 | 0.943107 | 0.967105 |
| GO:0043067 | regulation of programmed cell death | 43 | -0.201163 | -0.649516 | 0.944924 | 0.967105 |
| GO:0006725 | cellular aromatic compound metabolic process | 74 | -0.201560 | -0.688196 | 0.925620 | 0.967105 |
| GO:0008285 | negative regulation of cell proliferation | 17 | -0.202079 | -0.560302 | 0.971598 | 0.967105 |
| GO:0071417 | cellular response to organonitrogen compound | 19 | -0.202103 | -0.572113 | 0.957895 | 0.967105 |
| GO:1901699 | cellular response to nitrogen compound | 19 | -0.202103 | -0.572113 | 0.957895 | 0.967105 |
| GO:0022402 | cell cycle process | 16 | -0.203677 | -0.554962 | 0.971223 | 0.967105 |
| GO:0009059 | macromolecule biosynthetic process | 63 | -0.205912 | -0.694151 | 0.900938 | 0.967105 |
| GO:0008283 | cell proliferation | 46 | -0.206189 | -0.673186 | 0.911040 | 0.967105 |
| GO:0000165 | MAPK cascade | 26 | -0.206309 | -0.618781 | 0.927190 | 0.967105 |
| GO:0023014 | signal transduction by protein phosphorylation | 26 | -0.206309 | -0.618781 | 0.927190 | 0.967105 |
| GO:1902903 | regulation of supramolecular fiber organization | 11 | -0.206380 | -0.518387 | 0.972010 | 0.967105 |
| GO:0051252 | regulation of RNA metabolic process | 53 | -0.207689 | -0.687844 | 0.914826 | 0.967105 |
| GO:0016070 | RNA metabolic process | 56 | -0.210406 | -0.703227 | 0.895833 | 0.967105 |
| GO:0070848 | response to growth factor | 21 | -0.213005 | -0.618089 | 0.929234 | 0.967105 |
| GO:0071363 | cellular response to growth factor stimulus | 21 | -0.213005 | -0.618089 | 0.929234 | 0.967105 |
| GO:0048585 | negative regulation of response to stimulus | 38 | -0.213872 | -0.680929 | 0.911281 | 0.967105 |
| GO:0010556 | regulation of macromolecule biosynthetic process | 59 | -0.213980 | -0.717644 | 0.894682 | 0.967105 |
| GO:0043547 | positive regulation of GTPase activity | 12 | -0.215057 | -0.553116 | 0.968828 | 0.967105 |
| GO:1901653 | cellular response to peptide | 12 | -0.215510 | -0.554281 | 0.968828 | 0.967105 |
| GO:0042221 | response to chemical | 97 | -0.215597 | -0.749548 | 0.875764 | 0.967105 |
| GO:0009967 | positive regulation of signal transduction | 45 | -0.217236 | -0.706819 | 0.888651 | 0.967105 |
| GO:0043405 | regulation of MAP kinase activity | 11 | -0.219449 | -0.551215 | 0.965649 | 0.967105 |
| GO:0051338 | regulation of transferase activity | 29 | -0.219613 | -0.666970 | 0.895740 | 0.967105 |
| GO:0120036 | plasma membrane bounded cell projection organization | 35 | -0.219740 | -0.687827 | 0.894040 | 0.967105 |
| GO:0048878 | chemical homeostasis | 31 | -0.219791 | -0.678621 | 0.897238 | 0.967105 |
| GO:0019637 | organophosphate metabolic process | 19 | -0.220070 | -0.622974 | 0.927485 | 0.967105 |
| GO:0007417 | central nervous system development | 24 | -0.220624 | -0.657973 | 0.878547 | 0.967105 |
| GO:0010942 | positive regulation of cell death | 21 | -0.221374 | -0.642375 | 0.911833 | 0.967105 |
| GO:0001568 | blood vessel development | 20 | -0.221681 | -0.635491 | 0.918415 | 0.967105 |
| GO:0001944 | vasculature development | 20 | -0.221681 | -0.635491 | 0.918415 | 0.967105 |
| GO:0072358 | cardiovascular system development | 20 | -0.221681 | -0.635491 | 0.918415 | 0.967105 |
| GO:0018130 | heterocycle biosynthetic process | 60 | -0.222593 | -0.745783 | 0.858491 | 0.967105 |
| GO:0019438 | aromatic compound biosynthetic process | 60 | -0.222593 | -0.745783 | 0.858491 | 0.967105 |
| GO:0010243 | response to organonitrogen compound | 27 | -0.223688 | -0.674552 | 0.893060 | 0.967105 |
| GO:1901360 | organic cyclic compound metabolic process | 76 | -0.223900 | -0.764621 | 0.859794 | 0.967105 |
| GO:0045597 | positive regulation of cell differentiation | 30 | -0.225266 | -0.693206 | 0.872928 | 0.967105 |
| GO:0065008 | regulation of biological quality | 87 | -0.225337 | -0.775029 | 0.848205 | 0.967105 |
| GO:0010941 | regulation of cell death | 45 | -0.225395 | -0.733365 | 0.862955 | 0.967105 |
| GO:0043549 | regulation of kinase activity | 28 | -0.226766 | -0.686708 | 0.886005 | 0.967105 |
| GO:0098771 | inorganic ion homeostasis | 17 | -0.226800 | -0.628846 | 0.943195 | 0.967105 |
| GO:0042981 | regulation of apoptotic process | 39 | -0.226960 | -0.722877 | 0.876503 | 0.967105 |
| GO:0051052 | regulation of DNA metabolic process | 13 | -0.227498 | -0.598131 | 0.933905 | 0.967105 |
| GO:0070647 | protein modification by small protein conjugation or removal | 20 | -0.230315 | -0.660241 | 0.896270 | 0.967105 |
| GO:0008544 | epidermis development | 13 | -0.231748 | -0.609306 | 0.930233 | 0.967105 |
| GO:0034645 | cellular macromolecule biosynthetic process | 61 | -0.232179 | -0.780530 | 0.825678 | 0.967105 |
| GO:0051056 | regulation of small GTPase mediated signal transduction | 10 | -0.232409 | -0.566147 | 0.960887 | 0.967105 |
| GO:0034654 | nucleobase-containing compound biosynthetic process | 59 | -0.233451 | -0.782943 | 0.812304 | 0.967105 |
| GO:0009611 | response to wounding | 22 | -0.233523 | -0.680147 | 0.876586 | 0.967105 |
| GO:0032774 | RNA biosynthetic process | 52 | -0.234309 | -0.774814 | 0.814542 | 0.967105 |
| GO:2001141 | regulation of RNA biosynthetic process | 52 | -0.234309 | -0.774814 | 0.814542 | 0.967105 |
| GO:0006928 | movement of cell or subcellular component | 42 | -0.235834 | -0.755462 | 0.834973 | 0.967105 |
| GO:0010720 | positive regulation of cell development | 14 | -0.237876 | -0.637091 | 0.909747 | 0.967105 |
| GO:0045935 | positive regulation of nucleobase-containing compound metabolic process | 37 | -0.238074 | -0.753577 | 0.836084 | 0.967105 |
| GO:0048514 | blood vessel morphogenesis | 19 | -0.239594 | -0.678242 | 0.873684 | 0.967105 |
| GO:0010647 | positive regulation of cell communication | 49 | -0.240793 | -0.790890 | 0.797024 | 0.967105 |
| GO:0023056 | positive regulation of signaling | 49 | -0.240793 | -0.790890 | 0.797024 | 0.967105 |
| GO:2000112 | regulation of cellular macromolecule biosynthetic process | 57 | -0.240811 | -0.803878 | 0.785340 | 0.967105 |
| GO:0007178 | transmembrane receptor protein serine/threonine kinase signaling pathway | 10 | -0.241385 | -0.588014 | 0.954368 | 0.967105 |
| GO:0048513 | animal organ development | 73 | -0.241514 | -0.823116 | 0.785492 | 0.967105 |
| GO:0019725 | cellular homeostasis | 20 | -0.244698 | -0.701476 | 0.856643 | 0.967105 |
| GO:0009058 | biosynthetic process | 85 | -0.245600 | -0.841815 | 0.764344 | 0.967105 |
| GO:0050793 | regulation of developmental process | 61 | -0.247089 | -0.830654 | 0.752610 | 0.967105 |
| GO:0043086 | negative regulation of catalytic activity | 17 | -0.247663 | -0.686691 | 0.887574 | 0.967105 |
| GO:0010035 | response to inorganic substance | 19 | -0.247803 | -0.701481 | 0.861988 | 0.967105 |
| GO:0033554 | cellular response to stress | 38 | -0.248126 | -0.789985 | 0.788609 | 0.967105 |
| GO:1901362 | organic cyclic compound biosynthetic process | 62 | -0.248831 | -0.838314 | 0.746347 | 0.967105 |
| GO:0051493 | regulation of cytoskeleton organization | 13 | -0.249126 | -0.654996 | 0.903305 | 0.967105 |
| GO:1901566 | organonitrogen compound biosynthetic process | 25 | -0.249414 | -0.744295 | 0.822451 | 0.967105 |
| GO:0071407 | cellular response to organic cyclic compound | 16 | -0.249729 | -0.680443 | 0.908873 | 0.967105 |
| GO:0032502 | developmental process | 109 | -0.250304 | -0.870010 | 0.714863 | 0.967105 |
| GO:1902532 | negative regulation of intracellular signal transduction | 13 | -0.251087 | -0.660151 | 0.898409 | 0.967105 |
| GO:1901701 | cellular response to oxygen-containing compound | 34 | -0.251697 | -0.783364 | 0.786740 | 0.967105 |
| GO:0060548 | negative regulation of cell death | 32 | -0.251994 | -0.781262 | 0.774336 | 0.967105 |
| GO:1901698 | response to nitrogen compound | 28 | -0.252657 | -0.765114 | 0.805869 | 0.967105 |
| GO:0006351 | transcription, DNA-templated | 51 | -0.253075 | -0.831563 | 0.747881 | 0.967105 |
| GO:0006355 | regulation of transcription, DNA-templated | 51 | -0.253075 | -0.831563 | 0.747881 | 0.967105 |
| GO:0097659 | nucleic acid-templated transcription | 51 | -0.253075 | -0.831563 | 0.747881 | 0.967105 |
| GO:1903506 | regulation of nucleic acid-templated transcription | 51 | -0.253075 | -0.831563 | 0.747881 | 0.967105 |
| GO:0044249 | cellular biosynthetic process | 82 | -0.253468 | -0.866851 | 0.713697 | 0.967105 |
| GO:0051049 | regulation of transport | 42 | -0.254989 | -0.816822 | 0.769399 | 0.967105 |
| GO:0010629 | negative regulation of gene expression | 28 | -0.256348 | -0.776291 | 0.797968 | 0.967105 |
| GO:0051179 | localization | 115 | -0.257670 | -0.892009 | 0.682927 | 0.967105 |
| GO:0090066 | regulation of anatomical structure size | 11 | -0.258109 | -0.648320 | 0.913486 | 0.967105 |
| GO:0051254 | positive regulation of RNA metabolic process | 33 | -0.258162 | -0.803668 | 0.760750 | 0.967105 |
| GO:0051094 | positive regulation of developmental process | 40 | -0.258828 | -0.825054 | 0.754902 | 0.967105 |
| GO:0071900 | regulation of protein serine/threonine kinase activity | 13 | -0.259417 | -0.682052 | 0.876377 | 0.967105 |
| GO:0009966 | regulation of signal transduction | 72 | -0.259565 | -0.887391 | 0.689691 | 0.967105 |
| GO:0055082 | cellular chemical homeostasis | 19 | -0.261336 | -0.739792 | 0.829240 | 0.967105 |
| GO:0050769 | positive regulation of neurogenesis | 13 | -0.261445 | -0.687386 | 0.875153 | 0.967105 |
| GO:0051962 | positive regulation of nervous system development | 13 | -0.261445 | -0.687386 | 0.875153 | 0.967105 |
| GO:0051093 | negative regulation of developmental process | 26 | -0.261815 | -0.785259 | 0.778157 | 0.967105 |
| GO:1901652 | response to peptide | 13 | -0.262273 | -0.689561 | 0.871481 | 0.967105 |
| GO:0043065 | positive regulation of apoptotic process | 20 | -0.262373 | -0.752143 | 0.812354 | 0.967105 |
| GO:0043068 | positive regulation of programmed cell death | 20 | -0.262373 | -0.752143 | 0.812354 | 0.967105 |
| GO:0009891 | positive regulation of biosynthetic process | 36 | -0.263192 | -0.829996 | 0.741794 | 0.967105 |
| GO:0007049 | cell cycle | 25 | -0.263734 | -0.787027 | 0.775487 | 0.967105 |
| GO:1901576 | organic substance biosynthetic process | 80 | -0.264078 | -0.899977 | 0.661507 | 0.967105 |
| GO:0014070 | response to organic cyclic compound | 22 | -0.264825 | -0.771316 | 0.780854 | 0.967105 |
| GO:0006644 | phospholipid metabolic process | 10 | -0.267485 | -0.651593 | 0.902216 | 0.967105 |
| GO:0009890 | negative regulation of biosynthetic process | 27 | -0.268098 | -0.808474 | 0.757679 | 0.967105 |
| GO:0030155 | regulation of cell adhesion | 26 | -0.269435 | -0.808116 | 0.759954 | 0.967105 |
| GO:0006511 | ubiquitin-dependent protein catabolic process | 11 | -0.270606 | -0.679712 | 0.882952 | 0.967105 |
| GO:0019941 | modification-dependent protein catabolic process | 11 | -0.270606 | -0.679712 | 0.882952 | 0.967105 |
| GO:0043632 | modification-dependent macromolecule catabolic process | 11 | -0.270606 | -0.679712 | 0.882952 | 0.967105 |
| GO:0071495 | cellular response to endogenous stimulus | 41 | -0.270975 | -0.865496 | 0.695415 | 0.967105 |
| GO:0097190 | apoptotic signaling pathway | 17 | -0.272111 | -0.754479 | 0.800000 | 0.967105 |
| GO:0042113 | B cell activation | 10 | -0.272679 | -0.664245 | 0.890482 | 0.967105 |
| GO:0045893 | positive regulation of transcription, DNA-templated | 32 | -0.272839 | -0.845890 | 0.714602 | 0.967105 |
| GO:1902680 | positive regulation of RNA biosynthetic process | 32 | -0.272839 | -0.845890 | 0.714602 | 0.967105 |
| GO:1903508 | positive regulation of nucleic acid-templated transcription | 32 | -0.272839 | -0.845890 | 0.714602 | 0.967105 |
| GO:0010646 | regulation of cell communication | 76 | -0.274259 | -0.936598 | 0.598969 | 0.967105 |
| GO:0023051 | regulation of signaling | 76 | -0.274259 | -0.936598 | 0.598969 | 0.967105 |
| GO:0030001 | metal ion transport | 13 | -0.274464 | -0.721614 | 0.845777 | 0.967105 |
| GO:0042060 | wound healing | 20 | -0.275110 | -0.788656 | 0.766900 | 0.967105 |
| GO:0050801 | ion homeostasis | 18 | -0.275122 | -0.770349 | 0.788666 | 0.967105 |
| GO:0071396 | cellular response to lipid | 19 | -0.275691 | -0.780428 | 0.778947 | 0.967105 |
| GO:0051649 | establishment of localization in cell | 34 | -0.278877 | -0.867957 | 0.690608 | 0.967105 |
| GO:0051234 | establishment of localization | 99 | -0.279928 | -0.970899 | 0.558494 | 0.967105 |
| GO:1903530 | regulation of secretion by cell | 19 | -0.280056 | -0.792783 | 0.763743 | 0.967105 |
| GO:0034762 | regulation of transmembrane transport | 11 | -0.280399 | -0.704309 | 0.856234 | 0.967105 |
| GO:0097191 | extrinsic apoptotic signaling pathway | 11 | -0.280633 | -0.704897 | 0.853690 | 0.967105 |
| GO:0010628 | positive regulation of gene expression | 40 | -0.280999 | -0.895727 | 0.644880 | 0.967105 |
| GO:0072359 | circulatory system development | 26 | -0.281531 | -0.844395 | 0.711035 | 0.967105 |
| GO:0045596 | negative regulation of cell differentiation | 19 | -0.281617 | -0.797202 | 0.761404 | 0.967105 |
| GO:0031328 | positive regulation of cellular biosynthetic process | 35 | -0.281756 | -0.881946 | 0.674393 | 0.967105 |
| GO:0002790 | peptide secretion | 15 | -0.283132 | -0.768802 | 0.795238 | 0.967105 |
| GO:0009306 | protein secretion | 15 | -0.283132 | -0.768802 | 0.795238 | 0.967105 |
| GO:0006810 | transport | 98 | -0.283480 | -0.985527 | 0.527439 | 0.967105 |
| GO:0048856 | anatomical structure development | 102 | -0.283902 | -0.987409 | 0.534553 | 0.967105 |
| GO:0050877 | nervous system process | 16 | -0.286113 | -0.779579 | 0.787770 | 0.967105 |
| GO:0010557 | positive regulation of macromolecule biosynthetic process | 34 | -0.286207 | -0.890771 | 0.659669 | 0.967105 |
| GO:0045944 | positive regulation of transcription by RNA polymerase II | 24 | -0.286890 | -0.855599 | 0.688990 | 0.967105 |
| GO:0031327 | negative regulation of cellular biosynthetic process | 26 | -0.289001 | -0.866800 | 0.675768 | 0.967105 |
| GO:0001933 | negative regulation of protein phosphorylation | 11 | -0.289243 | -0.726523 | 0.818066 | 0.967105 |
| GO:0009628 | response to abiotic stimulus | 30 | -0.291474 | -0.896946 | 0.632044 | 0.967105 |
| GO:0015031 | protein transport | 33 | -0.292027 | -0.909089 | 0.637266 | 0.967105 |
| GO:0048731 | system development | 88 | -0.293572 | -1.011296 | 0.466189 | 0.967105 |
| GO:0009968 | negative regulation of signal transduction | 31 | -0.294251 | -0.908524 | 0.628729 | 0.967105 |
| GO:0001525 | angiogenesis | 14 | -0.295340 | -0.790993 | 0.772563 | 0.967105 |
| GO:0051346 | negative regulation of hydrolase activity | 10 | -0.296162 | -0.721451 | 0.838331 | 0.967105 |
| GO:0032879 | regulation of localization | 56 | -0.297141 | -0.993115 | 0.504167 | 0.967105 |
| GO:0033993 | response to lipid | 23 | -0.298399 | -0.869906 | 0.672434 | 0.967105 |
| GO:0046907 | intracellular transport | 29 | -0.299523 | -0.909659 | 0.615471 | 0.967105 |
| GO:0080135 | regulation of cellular response to stress | 17 | -0.299572 | -0.830618 | 0.714793 | 0.967105 |
| GO:0022603 | regulation of anatomical structure morphogenesis | 28 | -0.299748 | -0.907717 | 0.610609 | 0.967105 |
| GO:0051726 | regulation of cell cycle | 19 | -0.299871 | -0.848877 | 0.688889 | 0.967105 |
| GO:0051223 | regulation of protein transport | 18 | -0.300170 | -0.840486 | 0.708383 | 0.967105 |
| GO:0090087 | regulation of peptide transport | 18 | -0.300170 | -0.840486 | 0.708383 | 0.967105 |
| GO:0031400 | negative regulation of protein modification process | 14 | -0.300580 | -0.805027 | 0.756919 | 0.967105 |
| GO:0007267 | cell-cell signaling | 33 | -0.300920 | -0.936774 | 0.592062 | 0.967105 |
| GO:0007275 | multicellular organism development | 95 | -0.301550 | -1.042599 | 0.419125 | 0.967105 |
| GO:0010648 | negative regulation of cell communication | 33 | -0.302697 | -0.942305 | 0.585447 | 0.967105 |
| GO:0023057 | negative regulation of signaling | 33 | -0.302697 | -0.942305 | 0.585447 | 0.967105 |
| GO:0009719 | response to endogenous stimulus | 45 | -0.304000 | -0.989121 | 0.517131 | 0.967105 |
| GO:0001819 | positive regulation of cytokine production | 11 | -0.304316 | -0.764385 | 0.779898 | 0.967105 |
| GO:0007346 | regulation of mitotic cell cycle | 12 | -0.305062 | -0.784603 | 0.765586 | 0.967105 |
| GO:0046677 | response to antibiotic | 14 | -0.305156 | -0.817283 | 0.731649 | 0.967105 |
| GO:0051046 | regulation of secretion | 20 | -0.306311 | -0.878099 | 0.663170 | 0.967105 |
| GO:1901342 | regulation of vasculature development | 11 | -0.307009 | -0.771149 | 0.770992 | 0.967105 |
| GO:0015833 | peptide transport | 34 | -0.307709 | -0.957692 | 0.555801 | 0.967105 |
| GO:0010558 | negative regulation of macromolecule biosynthetic process | 25 | -0.308653 | -0.921075 | 0.593356 | 0.967105 |
| GO:0051640 | organelle localization | 14 | -0.311816 | -0.835119 | 0.708785 | 0.967105 |
| GO:0009991 | response to extracellular stimulus | 11 | -0.316671 | -0.795418 | 0.735369 | 0.967105 |
| GO:0003008 | system process | 29 | -0.318155 | -0.966246 | 0.539238 | 0.967105 |
| GO:0034622 | cellular protein-containing complex assembly | 10 | -0.318962 | -0.776991 | 0.779661 | 0.967105 |
| GO:0042886 | amide transport | 35 | -0.320566 | -1.003430 | 0.474614 | 0.967105 |
| GO:0060284 | regulation of cell development | 20 | -0.323569 | -0.927574 | 0.582751 | 0.967105 |
| GO:0009913 | epidermal cell differentiation | 11 | -0.324990 | -0.816312 | 0.720102 | 0.967105 |
| GO:0010038 | response to metal ion | 12 | -0.325136 | -0.836233 | 0.710723 | 0.967105 |
| GO:0045184 | establishment of protein localization | 35 | -0.325304 | -1.018262 | 0.450331 | 0.967105 |
| GO:0045934 | negative regulation of nucleobase-containing compound metabolic process | 26 | -0.328034 | -0.983869 | 0.494881 | 0.967105 |
| GO:0032870 | cellular response to hormone stimulus | 22 | -0.328347 | -0.956325 | 0.554787 | 0.967105 |
| GO:0000278 | mitotic cell cycle | 13 | -0.328760 | -0.864367 | 0.657283 | 0.967105 |
| GO:0048609 | multicellular organismal reproductive process | 14 | -0.330011 | -0.883852 | 0.642599 | 0.967105 |
| GO:0009653 | anatomical structure morphogenesis | 58 | -0.330136 | -1.104882 | 0.320459 | 0.967105 |
| GO:0022604 | regulation of cell morphogenesis | 12 | -0.330308 | -0.849535 | 0.697007 | 0.967105 |
| GO:0030307 | positive regulation of cell growth | 11 | -0.330319 | -0.829697 | 0.701018 | 0.967105 |
| GO:0002285 | lymphocyte activation involved in immune response | 10 | -0.332291 | -0.809461 | 0.735332 | 0.967105 |
| GO:0014065 | phosphatidylinositol 3-kinase signaling | 12 | -0.332575 | -0.855365 | 0.683292 | 0.967105 |
| GO:0048015 | phosphatidylinositol-mediated signaling | 12 | -0.332575 | -0.855365 | 0.683292 | 0.967105 |
| GO:0048017 | inositol lipid-mediated signaling | 12 | -0.332575 | -0.855365 | 0.683292 | 0.967105 |
| GO:2000113 | negative regulation of cellular macromolecule biosynthetic process | 24 | -0.332693 | -0.992199 | 0.501703 | 0.967105 |
| GO:0044283 | small molecule biosynthetic process | 12 | -0.332696 | -0.855676 | 0.683292 | 0.967105 |
| GO:0070201 | regulation of establishment of protein localization | 19 | -0.334042 | -0.945608 | 0.575439 | 0.967105 |
| GO:0001501 | skeletal system development | 10 | -0.334953 | -0.815945 | 0.727510 | 0.967105 |
| GO:0048729 | tissue morphogenesis | 22 | -0.335484 | -0.977113 | 0.527105 | 0.967105 |
| GO:0071375 | cellular response to peptide hormone stimulus | 10 | -0.335811 | -0.818034 | 0.722295 | 0.967105 |
| GO:0043687 | post-translational protein modification | 11 | -0.335841 | -0.843568 | 0.687023 | 0.967105 |
| GO:0002791 | regulation of peptide secretion | 13 | -0.336112 | -0.883697 | 0.623011 | 0.967105 |
| GO:0050708 | regulation of protein secretion | 13 | -0.336112 | -0.883697 | 0.623011 | 0.967105 |
| GO:0010563 | negative regulation of phosphorus metabolic process | 12 | -0.336586 | -0.865680 | 0.664589 | 0.967105 |
| GO:0042326 | negative regulation of phosphorylation | 12 | -0.336586 | -0.865680 | 0.664589 | 0.967105 |
| GO:0045936 | negative regulation of phosphate metabolic process | 12 | -0.336586 | -0.865680 | 0.664589 | 0.967105 |
| GO:0010975 | regulation of neuron projection development | 14 | -0.337216 | -0.903146 | 0.611312 | 0.967105 |
| GO:0042493 | response to drug | 30 | -0.338139 | -1.040548 | 0.429834 | 0.967105 |
| GO:0043491 | protein kinase B signaling | 12 | -0.339127 | -0.872216 | 0.657107 | 0.967105 |
| GO:0051896 | regulation of protein kinase B signaling | 12 | -0.339127 | -0.872216 | 0.657107 | 0.967105 |
| GO:0051641 | cellular localization | 45 | -0.340393 | -1.107533 | 0.330835 | 0.967105 |
| GO:0050767 | regulation of neurogenesis | 19 | -0.340933 | -0.965116 | 0.541520 | 0.967105 |
| GO:0031175 | neuron projection development | 21 | -0.340978 | -0.989436 | 0.504640 | 0.967105 |
| GO:0007507 | heart development | 12 | -0.342406 | -0.880650 | 0.640898 | 0.967105 |
| GO:0045666 | positive regulation of neuron differentiation | 10 | -0.342419 | -0.834132 | 0.705346 | 0.967105 |
| GO:0006357 | regulation of transcription by RNA polymerase II | 36 | -0.342757 | -1.080913 | 0.366521 | 0.967105 |
| GO:0006366 | transcription by RNA polymerase II | 36 | -0.342757 | -1.080913 | 0.366521 | 0.967105 |
| GO:1901700 | response to oxygen-containing compound | 42 | -0.343299 | -1.099712 | 0.340984 | 0.967105 |
| GO:0050878 | regulation of body fluid levels | 23 | -0.344620 | -1.004652 | 0.484429 | 0.967105 |
| GO:0071705 | nitrogen compound transport | 41 | -0.345419 | -1.103272 | 0.326419 | 0.967105 |
| GO:0007596 | blood coagulation | 12 | -0.348787 | -0.897061 | 0.610973 | 0.967105 |
| GO:0007599 | hemostasis | 12 | -0.348787 | -0.897061 | 0.610973 | 0.967105 |
| GO:0050817 | coagulation | 12 | -0.348787 | -0.897061 | 0.610973 | 0.967105 |
| GO:0048511 | rhythmic process | 11 | -0.349562 | -0.878032 | 0.638677 | 0.967105 |
| GO:0051050 | positive regulation of transport | 30 | -0.349746 | -1.076268 | 0.385635 | 0.967105 |
| GO:0036293 | response to decreased oxygen levels | 11 | -0.349870 | -0.878807 | 0.638677 | 0.967105 |
| GO:0070482 | response to oxygen levels | 11 | -0.349870 | -0.878807 | 0.638677 | 0.967105 |
| GO:0051656 | establishment of organelle localization | 13 | -0.350787 | -0.922280 | 0.565483 | 0.967105 |
| GO:0009636 | response to toxic substance | 16 | -0.352130 | -0.959458 | 0.525180 | 0.967105 |
| GO:0048646 | anatomical structure formation involved in morphogenesis | 26 | -0.353936 | -1.061558 | 0.401593 | 0.967105 |
| GO:0045765 | regulation of angiogenesis | 10 | -0.353998 | -0.862340 | 0.664928 | 0.967105 |
| GO:0051960 | regulation of nervous system development | 20 | -0.355941 | -1.020375 | 0.448718 | 0.967105 |
| GO:0051897 | positive regulation of protein kinase B signaling | 10 | -0.356023 | -0.867271 | 0.658409 | 0.967105 |
| GO:0001666 | response to hypoxia | 10 | -0.357695 | -0.871345 | 0.646675 | 0.967105 |
| GO:0008104 | protein localization | 46 | -0.360680 | -1.177585 | 0.229368 | 0.967105 |
| GO:0000902 | cell morphogenesis | 23 | -0.363652 | -1.060135 | 0.405998 | 0.967105 |
| GO:0032989 | cellular component morphogenesis | 23 | -0.363652 | -1.060135 | 0.405998 | 0.967105 |
| GO:0007399 | nervous system development | 45 | -0.364174 | -1.184909 | 0.231263 | 0.967105 |
| GO:1901135 | carbohydrate derivative metabolic process | 12 | -0.364199 | -0.936700 | 0.558603 | 0.967105 |
| GO:0090287 | regulation of cellular response to growth factor stimulus | 10 | -0.365003 | -0.889148 | 0.627119 | 0.967105 |
| GO:0009888 | tissue development | 49 | -0.367160 | -1.205943 | 0.189160 | 0.967105 |
| GO:0022008 | neurogenesis | 33 | -0.367203 | -1.143114 | 0.288864 | 0.967105 |
| GO:0048699 | generation of neurons | 33 | -0.367203 | -1.143114 | 0.288864 | 0.967105 |
| GO:0006886 | intracellular protein transport | 12 | -0.367686 | -0.945670 | 0.546135 | 0.967105 |
| GO:0035239 | tube morphogenesis | 30 | -0.368073 | -1.132663 | 0.312707 | 0.967105 |
| GO:0035295 | tube development | 30 | -0.368073 | -1.132663 | 0.312707 | 0.967105 |
| GO:0002696 | positive regulation of leukocyte activation | 11 | -0.368241 | -0.924951 | 0.577608 | 0.967105 |
| GO:0050867 | positive regulation of cell activation | 11 | -0.368241 | -0.924951 | 0.577608 | 0.967105 |
| GO:0051251 | positive regulation of lymphocyte activation | 11 | -0.368241 | -0.924951 | 0.577608 | 0.967105 |
| GO:0032504 | multicellular organism reproduction | 15 | -0.370920 | -1.007178 | 0.466667 | 0.967105 |
| GO:0048468 | cell development | 43 | -0.373687 | -1.206559 | 0.193305 | 0.967105 |
| GO:0045786 | negative regulation of cell cycle | 11 | -0.374895 | -0.941665 | 0.553435 | 0.967105 |
| GO:0043434 | response to peptide hormone | 11 | -0.375691 | -0.943665 | 0.548346 | 0.967105 |
| GO:0060341 | regulation of cellular localization | 21 | -0.377049 | -1.094104 | 0.361949 | 0.967105 |
| GO:0033036 | macromolecule localization | 53 | -0.377858 | -1.251425 | 0.147213 | 0.967105 |
| GO:0001558 | regulation of cell growth | 14 | -0.378347 | -1.013307 | 0.448857 | 0.967105 |
| GO:0016049 | cell growth | 14 | -0.378347 | -1.013307 | 0.448857 | 0.967105 |
| GO:0055085 | transmembrane transport | 19 | -0.381908 | -1.081108 | 0.368421 | 0.967105 |
| GO:0002009 | morphogenesis of an epithelium | 20 | -0.384529 | -1.102326 | 0.355478 | 0.967105 |
| GO:0071702 | organic substance transport | 46 | -0.385659 | -1.259136 | 0.135048 | 0.907547 |
| GO:0009725 | response to hormone | 25 | -0.387826 | -1.157342 | 0.288660 | 0.967105 |
| GO:0051253 | negative regulation of RNA metabolic process | 19 | -0.387906 | -1.098086 | 0.341520 | 0.967105 |
| GO:0030182 | neuron differentiation | 31 | -0.396208 | -1.223323 | 0.190055 | 0.967105 |
| GO:0034220 | ion transmembrane transport | 13 | -0.397803 | -1.045895 | 0.397797 | 0.967105 |
| GO:0048667 | cell morphogenesis involved in neuron differentiation | 13 | -0.400287 | -1.052424 | 0.394125 | 0.967105 |
| GO:0007167 | enzyme linked receptor protein signaling pathway | 33 | -0.400845 | -1.247843 | 0.155458 | 0.967105 |
| GO:0045785 | positive regulation of cell adhesion | 17 | -0.401328 | -1.112755 | 0.334911 | 0.967105 |
| GO:0000122 | negative regulation of transcription by RNA polymerase II | 16 | -0.401367 | -1.093613 | 0.339329 | 0.967105 |
| GO:0070997 | neuron death | 11 | -0.402002 | -1.009752 | 0.470738 | 0.967105 |
| GO:1901214 | regulation of neuron death | 11 | -0.402002 | -1.009752 | 0.470738 | 0.967105 |
| GO:0000904 | cell morphogenesis involved in differentiation | 17 | -0.402405 | -1.115741 | 0.328994 | 0.967105 |
| GO:0006812 | cation transport | 17 | -0.405652 | -1.124746 | 0.317160 | 0.967105 |
| GO:0030855 | epithelial cell differentiation | 29 | -0.406239 | -1.233758 | 0.190583 | 0.967105 |
| GO:0023061 | signal release | 14 | -0.408888 | -1.095102 | 0.351384 | 0.967105 |
| GO:0040007 | growth | 27 | -0.410551 | -1.238055 | 0.191126 | 0.967105 |
| GO:0032880 | regulation of protein localization | 26 | -0.415213 | -1.245347 | 0.188851 | 0.967105 |
| GO:0009914 | hormone transport | 11 | -0.415264 | -1.043065 | 0.428753 | 0.967105 |
| GO:0046879 | hormone secretion | 11 | -0.415264 | -1.043065 | 0.428753 | 0.967105 |
| GO:0032990 | cell part morphogenesis | 16 | -0.415926 | -1.133283 | 0.294964 | 0.967105 |
| GO:0048812 | neuron projection morphogenesis | 16 | -0.415926 | -1.133283 | 0.294964 | 0.967105 |
| GO:0048858 | cell projection morphogenesis | 16 | -0.415926 | -1.133283 | 0.294964 | 0.967105 |
| GO:0120039 | plasma membrane bounded cell projection morphogenesis | 16 | -0.415926 | -1.133283 | 0.294964 | 0.967105 |
| GO:0006629 | lipid metabolic process | 24 | -0.415981 | -1.240591 | 0.203178 | 0.967105 |
| GO:0045664 | regulation of neuron differentiation | 16 | -0.417471 | -1.137492 | 0.290168 | 0.967105 |
| GO:0017144 | drug metabolic process | 11 | -0.417928 | -1.049755 | 0.419847 | 0.967105 |
| GO:0048666 | neuron development | 25 | -0.418863 | -1.249960 | 0.195876 | 0.967105 |
| GO:0007409 | axonogenesis | 10 | -0.421930 | -1.027821 | 0.443286 | 0.967105 |
| GO:0061564 | axon development | 10 | -0.421930 | -1.027821 | 0.443286 | 0.967105 |
| GO:0044281 | small molecule metabolic process | 27 | -0.422497 | -1.274081 | 0.161547 | 0.967105 |
| GO:0048568 | embryonic organ development | 11 | -0.423483 | -1.063709 | 0.396947 | 0.967105 |
| GO:0007169 | transmembrane receptor protein tyrosine kinase signaling pathway | 25 | -0.425241 | -1.268994 | 0.178694 | 0.967105 |
| GO:0045892 | negative regulation of transcription, DNA-templated | 18 | -0.427141 | -1.196008 | 0.233766 | 0.967105 |
| GO:1902679 | negative regulation of RNA biosynthetic process | 18 | -0.427141 | -1.196008 | 0.233766 | 0.967105 |
| GO:1903507 | negative regulation of nucleic acid-templated transcription | 18 | -0.427141 | -1.196008 | 0.233766 | 0.967105 |
| GO:0044255 | cellular lipid metabolic process | 21 | -0.427224 | -1.239700 | 0.194896 | 0.967105 |
| GO:0097305 | response to alcohol | 11 | -0.431074 | -1.082775 | 0.371501 | 0.967105 |
| GO:0048589 | developmental growth | 20 | -0.431268 | -1.236313 | 0.198135 | 0.967105 |
| GO:0044703 | multi-organism reproductive process | 17 | -0.433034 | -1.200666 | 0.229586 | 0.967105 |
| GO:0060429 | epithelium development | 38 | -0.436610 | -1.390084 | 0.064622 | 0.666628 |
| GO:0006811 | ion transport | 24 | -0.436830 | -1.302769 | 0.148695 | 0.967105 |
| GO:0045927 | positive regulation of growth | 13 | -0.443080 | -1.164936 | 0.279070 | 0.967105 |
| GO:0002064 | epithelial cell development | 12 | -0.444500 | -1.143230 | 0.291771 | 0.967105 |
| GO:0010817 | regulation of hormone levels | 15 | -0.445017 | -1.208377 | 0.219048 | 0.967105 |
| GO:0033365 | protein localization to organelle | 11 | -0.448414 | -1.126331 | 0.321883 | 0.967105 |
| GO:0016055 | Wnt signaling pathway | 13 | -0.452982 | -1.190968 | 0.257038 | 0.967105 |
| GO:0198738 | cell-cell signaling by wnt | 13 | -0.452982 | -1.190968 | 0.257038 | 0.967105 |
| GO:0071214 | cellular response to abiotic stimulus | 11 | -0.456544 | -1.146752 | 0.298982 | 0.967105 |
| GO:0104004 | cellular response to environmental stimulus | 11 | -0.456544 | -1.146752 | 0.298982 | 0.967105 |
| GO:0008610 | lipid biosynthetic process | 15 | -0.458513 | -1.245023 | 0.184524 | 0.967105 |
| GO:0000003 | reproduction | 29 | -0.460417 | -1.398297 | 0.073991 | 0.726929 |
| GO:0022414 | reproductive process | 29 | -0.460417 | -1.398297 | 0.073991 | 0.726929 |
| GO:0051047 | positive regulation of secretion | 10 | -0.460830 | -1.122580 | 0.327249 | 0.967105 |
| GO:1903532 | positive regulation of secretion by cell | 10 | -0.460830 | -1.122580 | 0.327249 | 0.967105 |
| GO:0040008 | regulation of growth | 21 | -0.461343 | -1.338706 | 0.102088 | 0.853721 |
| GO:1905114 | cell surface receptor signaling pathway involved in cell-cell signaling | 14 | -0.463161 | -1.240458 | 0.193742 | 0.967105 |
| GO:0003013 | circulatory system process | 11 | -0.465638 | -1.169594 | 0.273537 | 0.967105 |
| GO:0008015 | blood circulation | 11 | -0.465638 | -1.169594 | 0.273537 | 0.967105 |
| GO:0009790 | embryo development | 19 | -0.465998 | -1.319150 | 0.122807 | 0.883850 |
| GO:1903827 | regulation of cellular protein localization | 14 | -0.469657 | -1.257859 | 0.185319 | 0.967105 |
| GO:0048545 | response to steroid hormone | 10 | -0.471368 | -1.148252 | 0.301173 | 0.967105 |
| GO:0006325 | chromatin organization | 10 | -0.471712 | -1.149090 | 0.301173 | 0.967105 |
| GO:0034613 | cellular protein localization | 27 | -0.473448 | -1.427728 | 0.055745 | 0.627333 |
| GO:0070727 | cellular macromolecule localization | 27 | -0.473448 | -1.427728 | 0.055745 | 0.627333 |
| GO:0019752 | carboxylic acid metabolic process | 11 | -0.474274 | -1.191285 | 0.250636 | 0.967105 |
| GO:0019953 | sexual reproduction | 10 | -0.477526 | -1.163254 | 0.279009 | 0.967105 |
| GO:0051222 | positive regulation of protein transport | 11 | -0.483555 | -1.214599 | 0.226463 | 0.967105 |
| GO:1904951 | positive regulation of establishment of protein localization | 11 | -0.483555 | -1.214599 | 0.226463 | 0.967105 |
| GO:0006082 | organic acid metabolic process | 12 | -0.484219 | -1.245386 | 0.205736 | 0.967105 |
| GO:0043436 | oxoacid metabolic process | 12 | -0.484219 | -1.245386 | 0.205736 | 0.967105 |
| GO:0072657 | protein localization to membrane | 13 | -0.486217 | -1.278349 | 0.171359 | 0.967105 |
| GO:0009887 | animal organ morphogenesis | 21 | -0.486929 | -1.412949 | 0.069606 | 0.706265 |
| GO:0003006 | developmental process involved in reproduction | 21 | -0.493108 | -1.430879 | 0.059165 | 0.642267 |
| GO:0060562 | epithelial tube morphogenesis | 14 | -0.496064 | -1.328582 | 0.129964 | 0.907104 |
| GO:0048732 | gland development | 17 | -0.496139 | -1.375638 | 0.088757 | 0.798246 |
| GO:0001655 | urogenital system development | 10 | -0.496458 | -1.209371 | 0.241199 | 0.967105 |
| GO:0007548 | sex differentiation | 14 | -0.499531 | -1.337867 | 0.121540 | 0.883850 |
| GO:0008406 | gonad development | 14 | -0.499531 | -1.337867 | 0.121540 | 0.883850 |
| GO:0045137 | development of primary sexual characteristics | 14 | -0.499531 | -1.337867 | 0.121540 | 0.883850 |
| GO:0009792 | embryo development ending in birth or egg hatching | 12 | -0.500765 | -1.287941 | 0.165835 | 0.967105 |
| GO:0043009 | chordate embryonic development | 12 | -0.500765 | -1.287941 | 0.165835 | 0.967105 |
| GO:1903829 | positive regulation of cellular protein localization | 10 | -0.505286 | -1.230878 | 0.207301 | 0.967105 |
| GO:0051276 | chromosome organization | 15 | -0.512540 | -1.391726 | 0.092857 | 0.798246 |
| GO:0050673 | epithelial cell proliferation | 12 | -0.514047 | -1.322101 | 0.135910 | 0.907547 |
| GO:0009314 | response to radiation | 12 | -0.519286 | -1.335578 | 0.124688 | 0.887075 |
| GO:0048638 | regulation of developmental growth | 13 | -0.525750 | -1.382289 | 0.105263 | 0.857268 |
| GO:0007017 | microtubule-based process | 12 | -0.526904 | -1.355170 | 0.114713 | 0.883850 |
| GO:0001654 | eye development | 10 | -0.530550 | -1.292419 | 0.153846 | 0.967105 |
| GO:0150063 | visual system development | 10 | -0.530550 | -1.292419 | 0.153846 | 0.967105 |
| GO:0048608 | reproductive structure development | 18 | -0.534062 | -1.495391 | 0.036600 | 0.471944 |
| GO:0061458 | reproductive system development | 18 | -0.534062 | -1.495391 | 0.036600 | 0.471944 |
| GO:0048880 | sensory system development | 11 | -0.538454 | -1.352494 | 0.113232 | 0.883850 |
| GO:0007423 | sensory organ development | 12 | -0.545817 | -1.403814 | 0.084788 | 0.783274 |
| GO:0055114 | oxidation-reduction process | 10 | -0.548409 | -1.335924 | 0.117340 | 0.883850 |
| GO:0048598 | embryonic morphogenesis | 14 | -0.558994 | -1.497123 | 0.042118 | 0.524405 |
| GO:0005975 | carbohydrate metabolic process | 10 | -0.567684 | -1.382878 | 0.091265 | 0.798246 |
| GO:0006820 | anion transport | 14 | -0.581552 | -1.557540 | 0.021661 | 0.362346 |
