# Supplementary material for: Identification of a prognostic chemoresistance-related gene signature associated with immune microenvironment in breast cancer
Source: Bioengineered. 2021 Oct 18;12(1):8419–34. doi: 10.1080/21655979.2021.1977768 (PMC8806919; doi:10.1080/21655979.2021.1977768)
Supplement: Supplemental Material [file KBIE_A_1977768_SM3462.zip › supplementary/Supplementary Table 8.doc]

| **Supplementary Table 8 The significant prognostic genes related breast cancer chemoresistance revealed by LASSO-penalized Cox regression** |
| --- |
|  |

| **Genes** | **Multivariate Cox regression analysis** | | | | |  | **LASSO analysis** |
| --- | --- | --- | --- | --- | --- | --- | --- |
|  | **HR**  **(95% CI )** | **Low 95% CI** | **High**  **95% CI** | ***p*-value** | **Coefficients** |  | **Coefficients** |
| C4A | 0.83 | 0.66 | 1.03 | 8.43E-02 | -0.1921 |  | -0.0236 |
| KDM7A | 1.94 | 1.18 | 3.17 | 8.57E-03 | 0.6618 |  | 0.1520 |
| MAPT | 0.68 | 0.50 | 0.93 | 1.68E-02 | -0.3804 |  | -0.1929 |
| PP14571 | 0.85 | 0.72 | 1.00 | 4.98E-02 | -0.1625 |  | -0.0352 |
