# Supplementary material for: Identification of a prognostic chemoresistance-related gene signature associated with immune microenvironment in breast cancer
Source: Bioengineered. 2021 Oct 18;12(1):8419–34. doi: 10.1080/21655979.2021.1977768 (PMC8806919; doi:10.1080/21655979.2021.1977768)
Supplement: Supplemental Material [file KBIE_A_1977768_SM3462.zip › supplementary/Supplementary Table 9.doc]

**Supplementary Table 9 T-test differences between 22 immune cells in the high- and low-risk groups**

| **Cell type** | ***P-*value** |
| --- | --- |
| Mast cells resting | 1.12E-07* |
| Dendritic cells resting | 5.13E-04* |
| T cells CD4 memory activated | 1.24E-03* |
| Dendritic cells activated | 2.91E-03* |
| T cells regulatory (Tregs) | 3.26E-03* |
| T cells follicular helper | 5.20E-02 |
| T cells CD8 | 1.02E-01 |
| Macrophages M1 | 1.42E-01 |
| T cells CD4 memory resting | 1.77E-01 |
| B cells memory | 1.92E-01 |
| NK cells resting | 1.95E-01 |
| Plasma cells | 2.27E-01 |
| Neutrophils | 2.28E-01 |
| NK cells activated | 2.46E-01 |
| Mast cells activated | 3.24E-01 |
| Monocytes | 4.40E-01 |
| Macrophages M2 | 6.87E-01 |
| Eosinophils | 7.31E-01 |
| T cells CD4 naive | 7.44E-01 |
| B cells naive | 7.98E-01 |
| T cells gamma delta | 8.45E-01 |
| Macrophages M0 | 9.97E-01 |
